# A Genetically Encoded Biosensor for Characterizing Transport and Metabolism of Glutarate

**DOI:** 10.1002/advs.202507046

**Published:** 2025-08-20

**Authors:** Kaiyu Gao, Hui Zhang, Yidong Liu, Xianzhi Xu, Wei Liu, Zhaoqi Kang, Rong Xu, Shuang Hou, Ping Han, Chuanjuan Lü, Cuiqing Ma, Ping Xu, Chao Gao

**Affiliations:** ^1^ State Key Laboratory of Microbial Technology Shandong University Qingdao 266237 P. R. China; ^2^ State Key Laboratory of Microbial Metabolism Joint International Research Laboratory of Metabolic & Developmental Sciences, and School of Life Sciences & Biotechnology Shanghai Jiao Tong University Shanghai 200240 P. R. China

**Keywords:** biosensors, glutarate, live‐cell detection, metabolism, transcriptional regulator, transport of glutarate

## Abstract

Glutarate is a platform chemical with diversified applications. It is also an endogenous metabolite involved in various physiological processes. Deficiency in glutaryl‐CoA dehydrogenase (GcdH) for glutarate catabolism induces the inherited metabolic disorder glutaric aciduria. In this study, a genetically encoded glutarate fluorescent biosensor Glusor is constructed and optimized based on transcriptional regulator CsiR and circularly permuted yellow fluorescent protein. Glusor can quantify glutarate in human body fluids and bacteria fermentation broth with good accuracy and precision, supporting the convenient diagnosis of glutaric aciduria and glutarate production monitoring. Then, the glutarate transport is characterized independent of radioactive substrate by using Glusor expressed in *Escherichia coli*. The functions of transporters KgtP and YnfM in the uptake and efflux of glutarate in *E. coli* are identified. Glusor is also used to reveal the catabolism of glutarate in bacteria and HEK293FT cells. The role of glutarate hydroxylase in glutarate catabolism of *E. coli* is identified through Glusor‐supported in situ glutarate detection. Spatially resolved in vivo analysis of glutarate in HEK293FT cells is realized and GcdH inhibition and hypoxia‐induced glutarate accumulation are elucidated by using Glusor. Overall, Glusor is a versatile tool for the detection of glutarate both in vitro and in vivo.

## Graphical abstract

Here, a genetically encoded glutarate biosensor, Glusor, is developed based on transcriptional regulator CsiR. Glusor can quantify glutarate concentrations with good accuracy and precision. Then, the role of KgtP and YnfM are identified and characterized by using Glusor. Glusor also allows glutarate spatiotemporal resolution in live cells, facilitating the elucidation of GcdH inhibition and hypoxia‐induced glutarate accumulation.

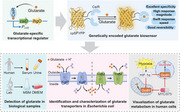

## 1 Introduction

Glutarate is an important building block for the production of polyesters and polyamides.^[^ ^1^, ^2^ ^]^ Developing efficient biotechnological processes for industrial production of glutarate from different precursors has attracted increasing attention.^[^ ^3^, ^4^, ^5^, ^6^ ^]^ Enhancing product efflux and blocking product uptake is an effective strategy for increasing the yield of chemicals produced through microbial fermentation. Overexpressing glutarate exporter YnfM in *Corynebacterium glutamicum* can enhance the fermentative glutarate production to 105.3 g L^−1^.^[^ ^4^ ^]^ However, the transporter responsible for the uptake of glutarate in microorganisms has never been reported. Glutarate is also an endogenous metabolite that participates in diverse physiological processes.^[^ ^7^ ^]^ Deficiency in mitochondrial glutaryl‐CoA dehydrogenase (GcdH) may block glutarate catabolism and induce an inherited metabolic disorder called glutaric aciduria.^[^ ^8^, ^9^, ^10^ ^]^ Glutarate also plays significant roles in immune processes involving T cell regulation.^[^ ^11^, ^12^ ^]^ It can directly regulate T cell metabolism through glutarylation of the E2 subunit of the pyruvate dehydrogenase complex, and modulate T cell anti‐tumor function through inhibiting.^[^ ^11^ ^]^

Nowadays, the detection of glutarate predominantly relies on techniques such as high‐performance liquid chromatography (HPLC),^[^ ^13^ ^]^ liquid chromatography‐tandem mass spectrometry (LC‐MS/MS),^[^ ^14^ ^]^ and gas chromatography‐tandem mass spectrometry (GC‐MS/MS).^[^ ^15^ ^]^ These methods are time‐consuming, expensive to perform, unsuitable for high‐throughput analysis, and incompatible with real‐time monitoring of the intracellular fluctuations of glutarate. The group of Keasling developed two transcription factor‐based glutarate biosensing systems based on the transcriptional regulatory factors CsiR and GcdR, their cognate promoters, and red fluorescent protein. The biosensors introduced in *Pseudomonas putida* KT2440 can specifically respond to exogenous glutarate and may be used in the screening of microorganisms for glutarate production.^[^ ^16^ ^]^ However, the signal output of these two sensors is dependent on the transcription and expression of the red fluorescent protein, and is unable to support real‐time monitoring and spatially resolved in vivo analysis of glutarate.

Genetically encoded fluorescent biosensors can transform chemical signals into luminescent readouts in real‐time.^[^ ^17^, ^18^, ^19^, ^20^, ^21^, ^22^, ^23^ ^]^ Here, we constructed a genetically encoded fluorescent glutarate biosensor based on circularly permuted fluorescent protein (cpFP) and glutarate‐specific transcriptional regulator CsiR. The developed biosensor Glusor achieved previously unavailable precise real‐time detection of glutarate. Then, we used Glusor for quantification of glutarate in different biological samples and identified the glutarate producing activities of different derivative strains of *P. putida* KT2440. In addition, we employed Glusor to reveal the transport and catabolism of glutarate in the model industrial strain *Escherichia coli*. KgtP and YnfM, the transporters responsible for uptake and efflux of glutarate in *E. coli*, were ascertained and characterized independent of radioactive substrate by using Glusor. The role of glutarate hydroxylase in glutarate catabolism was also identified in *E. coli*. Finally, we ascertained the suitability of Glusor for monitoring glutarate metabolism in HEK293FT cells and identified the role of GcdH in glutarate degradation. The distribution of glutarate across different cellular compartments was also elucidated based on the Glusor‐supported spatiotemporal glutarate assay.

## 2 Results

### 2.1 Identification of CsiR as a Recognition Element for Glutarate Biosensor Construction

**Figure 1:**
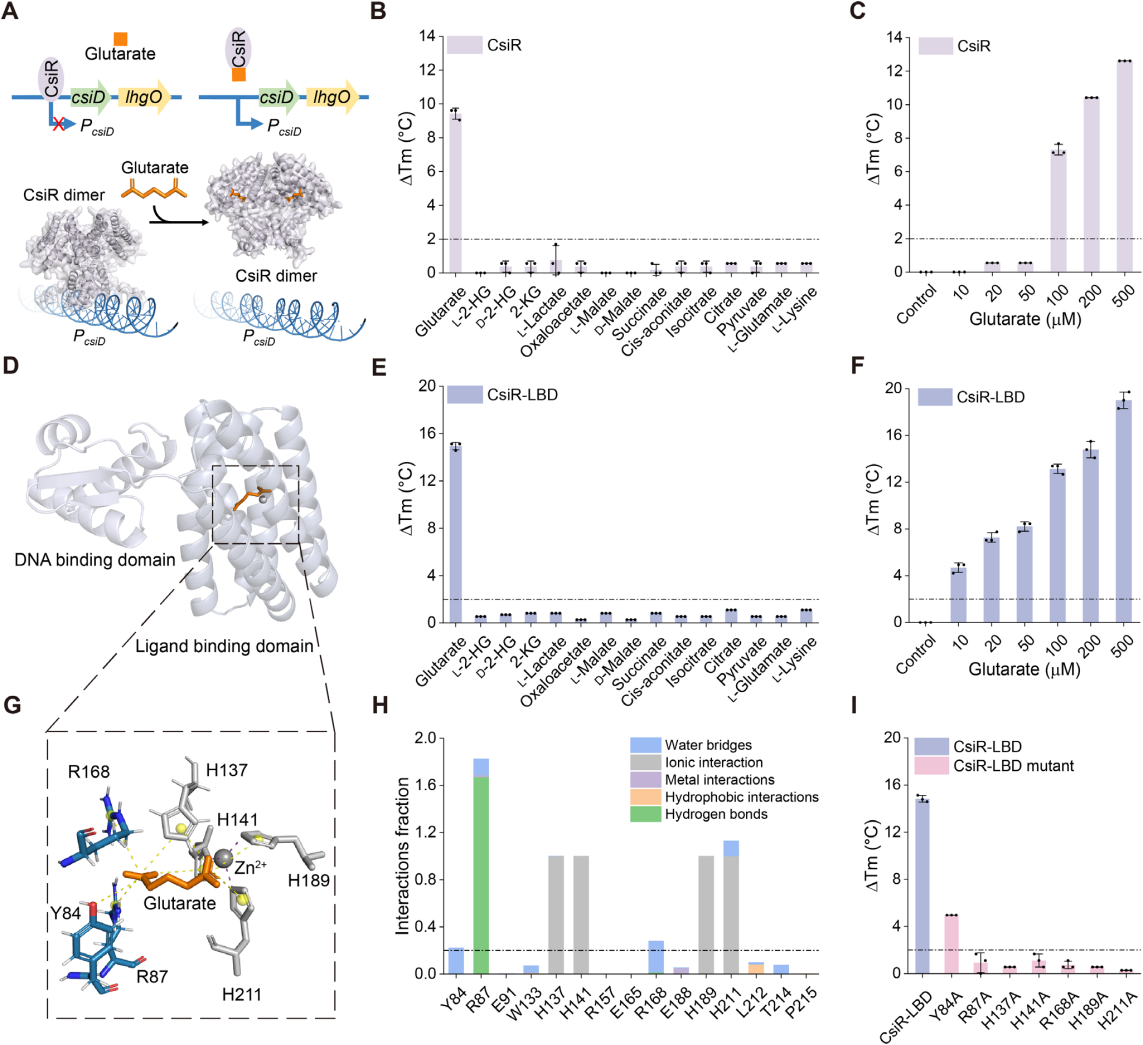
CsiR specifically binds to glutarate. A) Schematic representation of the regulation of the glutarate hydroxylation pathway by CsiR. *P*_csiD_, the promoter of the *csiD‐lhgO* operon. B) Identification of the ligand of CsiR by FTS assays. The ΔTm values indicate the changes in Tm compared to control (without ligand). The concentration of tested compounds was 100 µm. C) FTS analysis of CsiR binding to glutarate at varying concentrations. D) Molecular docking result of CsiR with glutarate. CsiR‐DBD and CsiR‐LBD are shown in white and cyan, respectively. E) Ligand identification of CsiR‐LBD by FTS assays. F) FTS analysis of CsiR‐LBD binding to glutarate at varying concentrations. G) Residues interacting with glutarate in CsiR‐LBD. Residues involved in ionic interactions are displayed in grey, other interacting residues are displayed in blue, and glutarate is shown in orange. H) Protein‐ligand contacts during molecular dynamics simulations. The interaction fraction exceeding 1.0 is due to multiple contacts on one single residue. I) FTS assays of CsiR‐LBD and its mutants to glutarate. All data shown are means ± standard deviations (s.d.) (*n* = 3 independent experiments).

We previously identified the functions of the glutarate hydroxylation pathway and the glutaryl‐CoA dehydrogenation pathway in glutarate degradation of *P. putida* KT2440.^[^ ^24^ ^]^ Glutarate hydroxylase CsiD and l‐2‐hydroxyglutarate (l‐2‐HG) oxidase LhgO are key enzymes in the glutarate hydroxylation pathway.^[^ ^24^, ^25^, ^26^ ^]^ The GntR family transcriptional regulator CsiR represses the expression of CsiD and LhgO, while glutarate can induce the transcription of *csiD* and *lhgO* (**Figure** **1**A).^[^ ^16^, ^27^ ^]^ In this study, we overexpressed His_6_‐tagged CsiR in *E. coli* BL21(DE3) and purified the transcriptional regulator through affinity chromatography (Figure S1, Supporting Information). Subsequently, we performed fluorescence‐based thermal shift (FTS) assays to ascertain the possible ligand that interacts with CsiR. Among the 15 tested compounds, only glutarate induced a significant increase in the melting temperature (Tm) of CsiR (Figure 1B). In addition, the elevation of the concentration of glutarate led to increased ΔTm value of CsiR, further supporting the specific interaction between CsiR and glutarate (Figure 1C).

We used Alphafold2 to predict the protein structure of CsiR.^[^ ^28^ ^]^ Like other transcriptional regulators of the GntR family, CsiR is also comprised of two domains, a DNA‐binding domain (DBD) and a ligand‐binding domain (LBD). Molecular docking performed through the Glide docking module of Schrödinger indicated that glutarate and a structural zinc ion are localized in the LBD of CsiR (Figure 1D; Figure S2A, Supporting Information).^[^ ^29^ ^]^ Subsequent molecular dynamics simulations indicated that glutarate binding obviously altered the conformation of helix α‐1 to α‐10 in CsiR. The RMSD of superimposition between glutarate‐free and glutarate‐bound states reached 2.25 Å (Figure S2B, Supporting Information). We also overexpressed and purified the LBD of CsiR (CsiR‐LBD) (Figure S1, Supporting Information), and identified the specific response of CsiR‐LBD to glutarate by FTS assays (Figure 1E,F). Considering their specific response to glutarate, CsiR or CsiR‐LBD can thus be employed as a recognition element for the development of a glutarate biosensor.

We further identified the possible residues involved in ligands (glutarate and structural zinc ion) binding of CsiR‐LBD (Figure 1G). The simulation spanning 500 ns for CsiR monomer in complex with glutarate and the structural zinc ion was performed (Figure S3, Supporting Information).^[^ ^30^ ^]^ The statistical evaluation of the amino acid interactions over the course of simulation revealed the identical glutarate binding sites. Residues Y84 and R168 may interact with glutarate predominantly through water bridges, while R87 may interact with glutarate via direct hydrogen bonds. Mutations of residues Y84, R87, and R168 to alanine resulted in decreased binding of CsiR‐LBD to glutarate. Among the three obtained mutants, only CsiR‐LBD^Y84A^ still retained an obviously decreased response to glutarate. These results suggested that the residues mentioned above played crucial roles in the binding of CsiR to glutarate. Residues H137, H141, H189, and H211 may only participate in ionic interactions with the zinc ion (Figure 1H). Mutations of residues H137, H141, H189, and H211 to alanine also abolished the binding between CsiR‐LBD and glutarate, indicating the role of the structural zinc ion in protein stability and glutarate binding.

### 2.2 Design and Optimization of the Glutarate Biosensor Glusor

**Figure 2:**
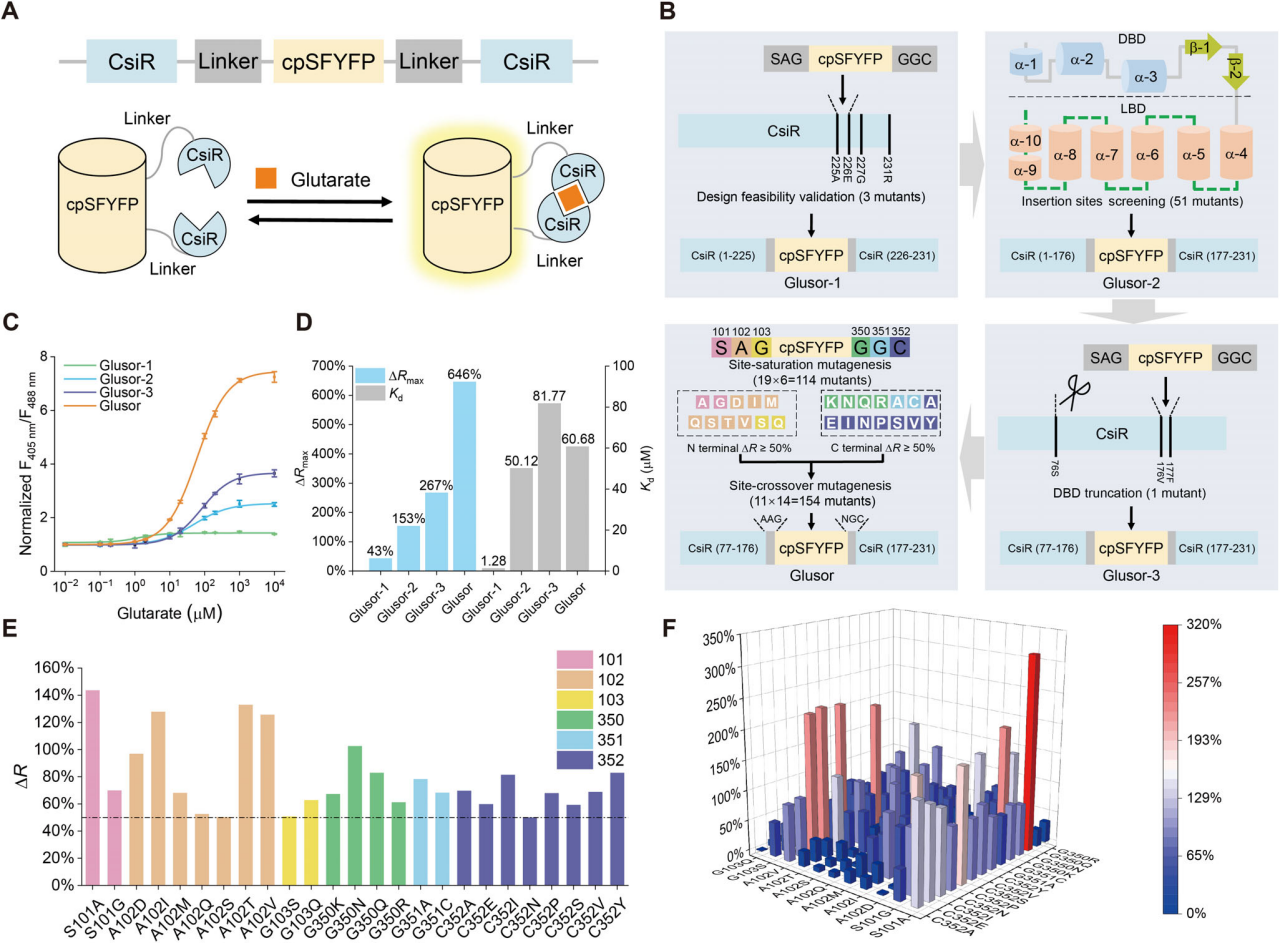
Design and optimization of Glusor. A) Schematic representation of the working principle of the CsiR‐based glutarate biosensor Glusor. B) Schematic representation of the process for the development of Glusor. The number of the insertion site in CsiR corresponds to the sequence of the original full‐length CsiR. The number of the linker site corresponds to the sequence of the full‐length biosensor. C) Dose‐response curves of Glusor‐1, Glusor‐2, Glusor‐3, and Glusor to glutarate. Data were normalized to the initial ratio. D) Comparison of the Δ*R*_max_ and *K*_d_ values of Glusor‐1, Glusor‐2, Glusor‐3, and Glusor to glutarate. E) Variants of Glusor‐3 with Δ*R* exceeding 50% for 1 mm glutarate. F) Δ*R* of different Glusor‐3 variants with site‐crossover mutagenesis for 1 mm glutarate. All data shown are means ± standard deviations (s.d.) (*n* = 3 independent experiments).

Then, we designed a glutarate biosensor composed of CsiR and circularly permuted yellow fluorescent protein with four superfolder sites (cpSFYFP).^[^ ^31^ ^]^ Conformational changes of CsiR induced by glutarate binding may alter the local environment of cpSFYFP and result in higher fluorescence (**Figure** **2**A). The construction and optimization of the glutarate biosensor were carried out through a four‐step workflow (Figure 2B). In Step 1, cpSFYFP was inserted in three separate sites within α‐10 (Figure S4, Supporting Information) at the C‐terminal of CsiR. The variant Glusor‐1 obtained by inserting cpSFYFP at position 225A/226E of CsiR exhibited a dose‐dependent response to glutarate. It exhibited a maximum ratio change (Δ*R*_max_) of 43% (Figure 2C) and an apparent dissociation constant (*K*_d_) of 1.28 µm toward glutarate (Figure 2D), indicating the feasibility of constructing a glutarate sensor based on the transcriptional regulator CsiR. In Step 2, cpSFYFP was inserted between amino acid residues located at the loops from α‐4 to α‐10 in the LBD of CsiR. Among the 51 obtained variants (Figure S5, Supporting Information), Glusor‐2 with cpSFYFP inserted between position 176 V/177F of CsiR exhibited a maximum Δ*R*_max_ of 153% (Figure 2C) and a *K*_d_ of 50.12 µm toward glutarate (Figure 2D). In Step 3, DBD of CsiR in Glusor‐2 was truncated (Figure 2B) and the obtained biosensor Glusor‐3 exhibited a Δ*R*_max_ of 267% (Figure 2C) and a *K*_d_ of 81.77 µm toward glutarate (Figure 2D).

In Step 4, the linkers between CsiR‐LBD and cpSFYFP were optimized. The saturation mutagenesis of the amino acids in linkers upstream (positions 101–103) and downstream (positions 350–352) of cpSFYFP was performed (Figure 2B). The responses of the resulting 114 variants to 1 mm glutarate were evaluated using the crude extracts of *E. coli* BL21(DE3) (Figure S6A, Supporting Information). Then, a combinatorial mutagenesis of amino acids in the upstream linker (11 variants with Δ*R* exceeding 50%) and downstream linker (14 variants with Δ*R* exceeding 50%) was conducted (Figure 2E). Among the 154 combinatorially mutated variants, the mutant with 101A and 350N substitutions exhibited the highest response to glutarate (Δ*R*_max_ = 646%) and an appropriate *K*_d_ of 60.68 µm (Figure 2F; Figure S6B, Supporting Information). This variant was designated as the optimal glutarate biosensor Glusor and its amino acid sequence can be found in Figure S7 (Supporting Information). Like the situation of CsiR and CsiR‐LBD, Glusor existed as dimeric forms (Figure S8, Supporting Information). Molecular dynamics simulations indicated that the glutarate binding significantly changed the conformation of helix α‐2, α‐3, and α‐5 in CsiR‐LBD of Glusor. The RMSD of superimposition between glutarate‐free and glutarate‐bound states of CsiR‐LBD reached 2.33 Å (Figure S9A,B, Supporting Information). In addition, the binding of glutarate also significantly changed the interactions of the fluorophore with surrounding residues in the cpSFYFP domain (Figure S9C, Supporting Information), which may partially account for glutarate‐induced fluorescence changes of Glusor.

### 2.3 Characterization of the Glutarate Biosensor Glusor

**Figure 3:**
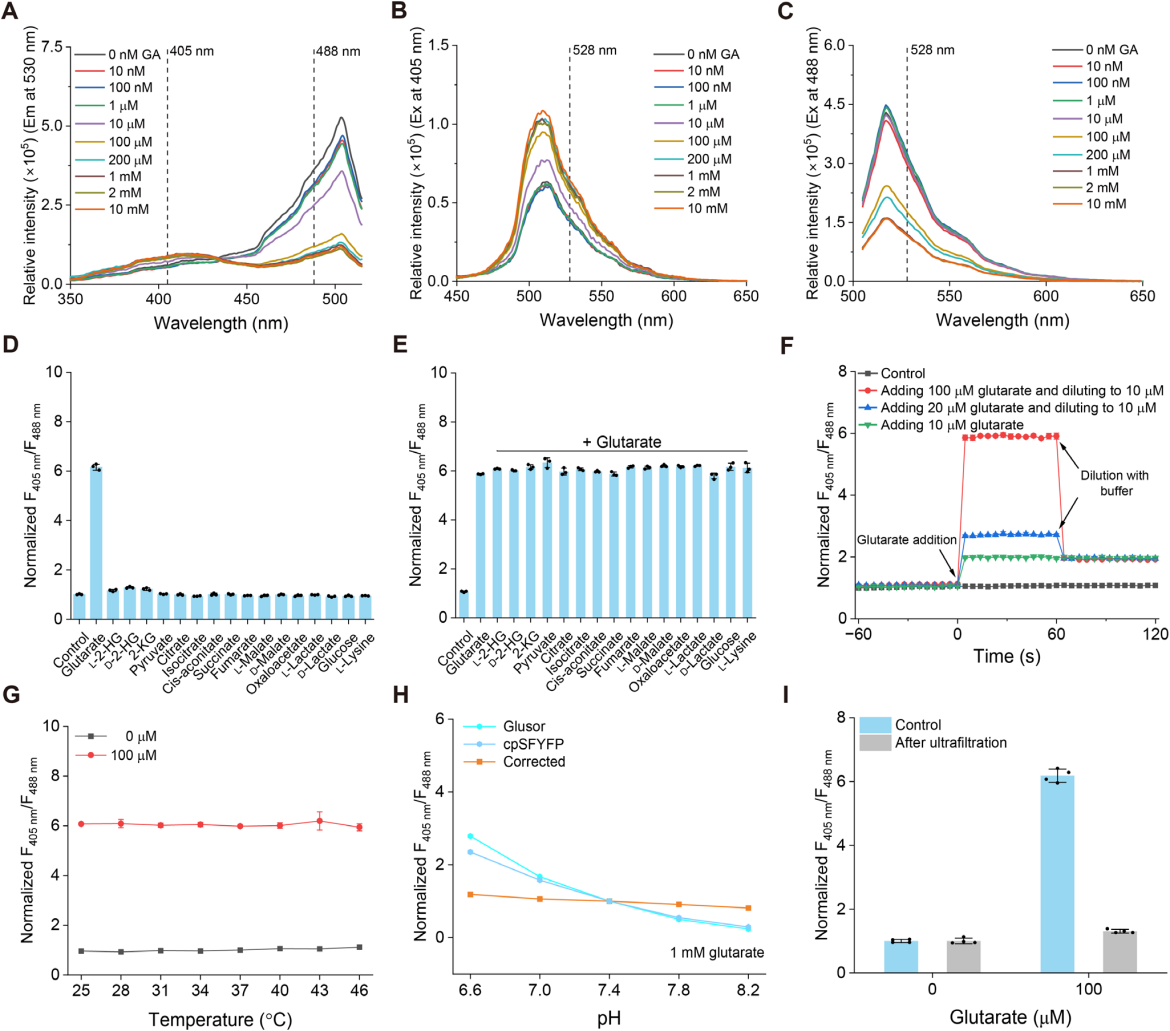
Characterization of Glusor in vitro. A) Fluorescence excitation spectra (emission at 530 nm) of Glusor at the indicated glutarate concentrations. B,C) Fluorescence emission spectra of Glusor with indicated glutarate concentrations at 405 (B) or 488 nm (C) excitation. D) Specificity of Glusor. The fluorescence ratios of Glusor were determined in the presence of 100 µm indicated metabolites. Data were normalized to the control without any metabolite. E) Selectivity of Glusor for glutarate in the presence of different metabolites. The fluorescence ratios of Glusor were measured in the absence of any metabolites (control), in the presence of glutarate alone (glutarate), or in the presence of glutarate and 100 µm indicated metabolite. Data were normalized to the control. F) Dynamic response of Glusor to glutarate. Glutarate was added in the detection system to the indicated concentrations at time point 0 s, and 50 mm Tris‐HCl buffer with suitable volume was added at time point 60 s to dilute the concentration of glutarate to 10 µm. Data were normalized to the initial ratio without glutarate. G) Temperature‐stability of Glusor. Data were normalized to the fluorescence ratio without glutarate at 37 °C. H) pH‐correction of the fluorescence ratios of Glusor in response to 1 mm glutarate by cpSFYFP. Data were normalized to the fluorescence ratio at pH 7.4. I) Reversibility analysis of Glusor. The fluorescence ratios of Glusor after adding and removing glutarate were measured. Data were normalized to the control without glutarate. All data shown are means ± standard deviations (s.d.) (*n* ≥ 3 independent experiments).

Glusor was overexpressed in *E. coli* BL21(DE3) and then purified by a Ni‐chelating chromatographic column for characterization (Figure S10, Supporting Information). Glusor has typical excitation and emission spectra of other cpFP‐based biosensors.^[^ ^32^, ^33^, ^34^ ^]^ It exhibited two excitation peaks at wavelengths of ≈405 and 488 nm, with a single emission peak at ≈528 nm. The presence of glutarate led to a dose‐dependent increase in fluorescence intensity upon excitation at 405 nm and a corresponding dose‐dependent decrease in fluorescence intensity when excited at 488 nm (**Figure** **3**A–C), resulting in a large change in the ratio between F_405 nm_ and F_488 nm_. No significant changes in F_405 nm_/F_488 nm_ of purified cpSFYFP in the presence of glutarate were detected, and thus we can conclude that the changes in Glusor fluorescence were not due to chromophore perturbation (Figure S11, Supporting Information). Then, 100 µm glutarate or its structural analogs, including l‐2‐HG, d‐2‐hydroxyglutarate (d‐2‐HG), 2‐KG, as well as compounds from the TCA cycle, l‐lactate, d‐lactate, l‐lysine, and glucose, were individually mixed with the purified Glusor to evaluate its specificity. As shown in Figure 3D, only glutarate significantly increased the fluorescence ratio of Glusor. In addition, the detection of glutarate by Glusor was unaffected by the presence of these chemicals (Figure 3E). These results demonstrated that Glusor has excellent specificity for glutarate. Mutations of residues related to glutarate binding resulted in an obviously decreased response of Glusor to glutarate. Actually, only the Glusor variant with the Y84A mutation still can response to glutarate (Figure S12A, Supporting Information). Compared with Glusor which has a *K*_d_ of 60.74 µm toward glutarate, the mutant exhibits an increased *K*_d_ value of 2.62 mm (Figure S12B, Supporting Information).

The dynamic response of Glusor to glutarate was monitored by using a fluorescence microplate reader. As shown in Figure 3F, the addition of glutarate elicited an immediate increase of the fluorescence ratios of Glusor. Diluting glutarate in the detection system with 50 mm Tris‐HCl buffer induced a rapid decrease of fluorescence ratios of Glusor to the level corresponding to the target glutarate concentration. The temperature and pH stability of Glusor for glutarate detection were also investigated. Glusor remains unaffected within the temperature range of 25 to 46 °C (Figure 3G). Consistent with other cpFP‐based biosensors,^[^ ^31^, ^35^ ^]^ the response of Glusor was susceptible to pH variations (Figure S13A, Supporting Information). However, detection of Glusor and cpSFYFP fluorescence at pH values ranging from 6.6 to 8.2 indicated that these two proteins displayed very similar sensitivities to pH in the presence of 1 mm glutarate (Figure S13B,C, Supporting Information). Thus, the glutarate detection by Glusor can be pH‐stabilized after correction with cpSFYFP (Figure 3H). The detection reversibility of Glusor was also evaluated by measuring the changes of fluorescence ratio after the addition and subsequent removal of glutarate in the detection system through ultrafiltration. As shown in Figure 3I, the increase in fluorescence ratio of Glusor induced by the addition of glutarate returned to baseline levels after the removal of glutarate, supporting its reversible glutarate binding for real‐time measurement.

### 2.4 In Vitro Detection of Glutarate in Biological Samples with Glusor

**Figure 4:**
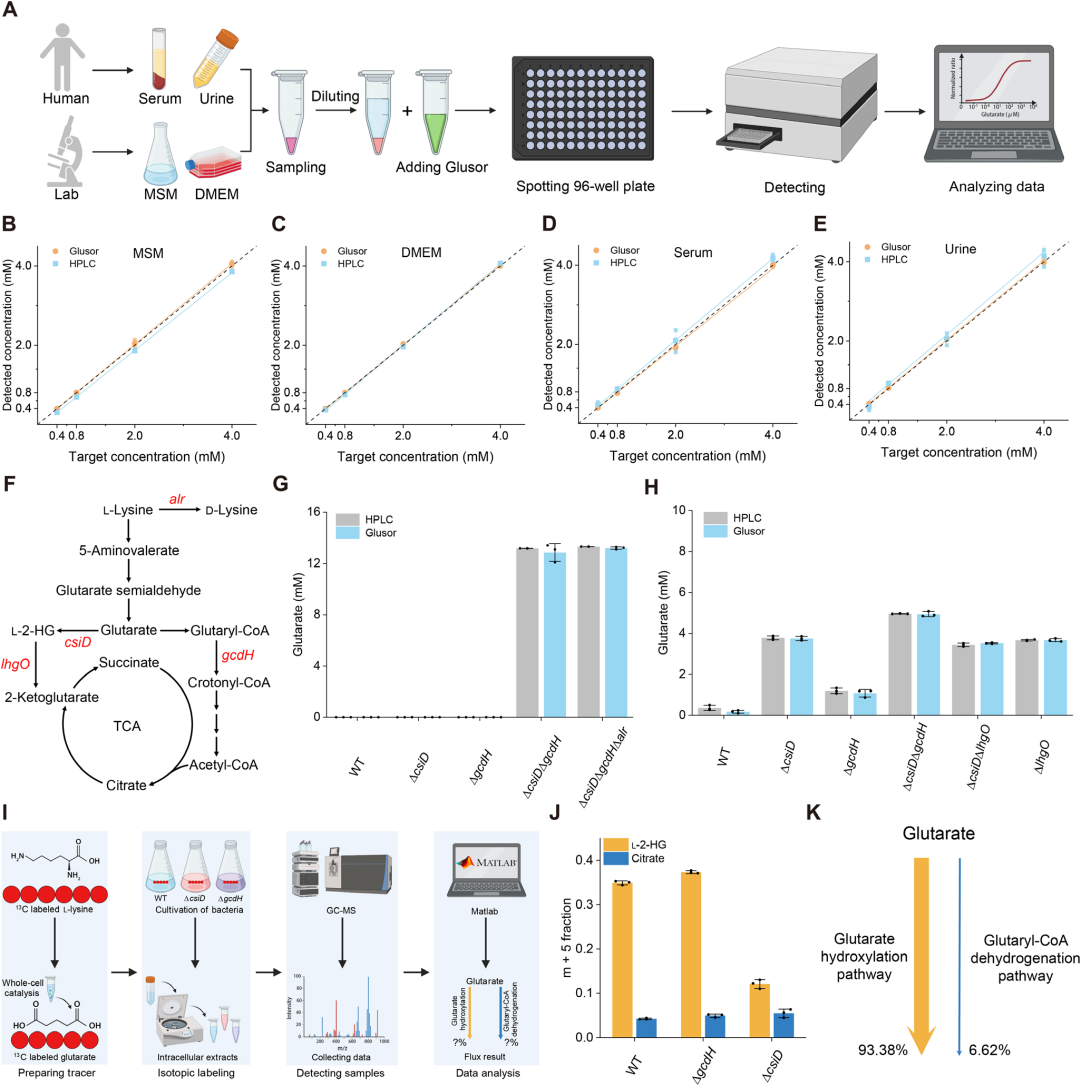
Detection of glutarate in various biological samples by using Glusor. A) Schematic representation of the workflow of Glusor‐based glutarate determination in various biological samples. The figure was generated using BioRender. B–E) Comparison of the quantification results of glutarate in MSM (B), DMEM (C), serum (D), and urine (E) obtained by Glusor and HPLC. The orange circles and cyan squares denote the quantitative results of Glusor and HPLC, respectively. The colored curves represent the fitting curves derived from corresponding quantitative data, with the black dashed line as the reference line. F) Anabolic and catabolic pathways of glutarate in *P. putida* KT2440. *alr*, alanine racemase. G) Detection of extracellular glutarate accumulation of *P. putida* KT2440 and its derivative strains using Glusor and HPLC. *P. putida* KT2440 and its derivative strains were cultured in MSM containing 5 g L^−1^ l‐lysine and 5 g L^−1^ glucose. H) Consumption of extracellular glutarate by *P. putida* KT2440 and its derivative strains. *P. putida* KT2440 and its derivative strains were cultured in MSM containing 20 mm glucose and 5 mm glutarate. The concentrations of glutarate at 6 h were detected by Glusor and HPLC. I) Schematic representation of the metabolic flux analysis procedure for glutarate catabolism. The figure was generated using BioRender. J) Fraction of the *m* + 5 isotopolog of the intracellular ^13^C‐glutarate‐derived l‐2‐HG and citrate fragment in *P. putida* KT2440, *P. putida* KT2440 (Δ*csiD*) and *P. putida* KT2440 (Δ*gcdH*). K) Flux distribution of glutarate catabolized through glutarate hydroxylation pathway and glutaryl‐CoA dehydrogenation pathway. All data shown are means ± standard deviations (s.d.) (*n* = 3 independent experiments).

The detection of glutarate in different biological samples with Glusor could be conducted in 96‐ or 384‐well plates for high‐throughput detection (**Figure** **4**A). Dose‐response curves for quantitation of glutarate in bacterial minimal salt medium (MSM), high‐glucose Dulbecco's modified Eagle medium (DMEM), commercially available human serum, and urine sample from healthy volunteers were constructed (Figure S14A–D, Supporting Information). The limits of detection (LODs) of Glusor for the detection of glutarate in MSM, DMEM, human serum, and urine were 0.23, 1.40, 0.21, and 1.62 µm, respectively. Then, glutarate at a range of concentrations (ranging from 0 to 2 mm) was added into these biological samples, and quantified by Glusor and HPLC. The results from Glusor showed close agreement with the results of HPLC (Figure 4B–E), and possessed good accuracy and precision (Table S1, Supporting Information).

Glutarate is an important dicarboxylic acid with diverse applications.^[^ ^5^, ^36^ ^]^ *P. putida* KT2440 can transform l‐lysine into glutarate and then catabolize glutarate through the glutarate hydroxylation pathway and the glutaryl‐CoA dehydrogenation pathway (Figure 4F).^[^ ^24^, ^26^ ^]^ *P. putida* KT2440, *P. putida* KT2440 (Δ*csiD*), *P. putida* KT2440 (Δ*gcdH*), *P. putida* KT2440 (Δ*csiD*Δ*gcdH*) and *P. putida* KT2440 (Δ*csiD*Δ*gcdH*Δ*alr*) were cultured in medium with 5 g L^−1^ glucose and 5 g L^−1^ l‐lysine. As shown in Figure 4G, the concentrations of glutarate accumulated by different recombinant strains could be readily determined by Glusor and the results were highly concordant with those of HPLC, indicating that Glusor can accurately detect the concentration of glutarate in pending test fermentation samples. *P. putida* KT2440, *P. putida* KT2440 (Δ*csiD*), and *P. putida* KT2440 (Δ*gcdH*) can use glutarate as the carbon source for growth.^[^ ^24^ ^]^ There was no accumulation of glutarate in the medium of these three strains. *P. putida* KT2440 (Δ*csiD*Δ*gcdH*) and *P. putida* KT2440 (Δ*csiD*Δ*gcdH*Δ*alr*) with deleted glutarate hydroxylation pathway and glutaryl‐CoA dehydrogenation pathway have been constructed to produce glutarate from l‐lysine.^[^ ^24^ ^]^ As expected, *P. putida* KT2440 (Δ*csiD*Δ*gcdH*) and *P. putida* KT2440 (Δ*csiD*Δ*gcdH*Δ*alr*) with blocked glutarate consumption could efficiently accumulate glutarate from l‐lysine (Figure 4G).

Glusor can also accurately monitor the consumption of glutarate during growth of *P. putida* KT2440 and its derivative strains in medium containing both glucose and glutarate as the carbon sources (Figure S15A–C, Supporting Information; Figure 4H). Interestingly, *P. putida* KT2440 (Δ*lhgO*) consumed 5 mm glutarate and accumulated 4.85 mm l‐2‐HG within 20 h (Figure S16D, Supporting Information), implying that glutarate may be predominantly catabolized via the hydroxylation pathway. Then, we prepared ^13^C‐labeled glutarate from ^13^C_6_‐l‐lysine using the whole cells of *P. putida* KT2440 (Δ*csiD*Δ*gcdH*Δ*alr*) as the biocatalyst and then conducted metabolic flux analysis as depicted in Figure 4I. We calculated the relative contributions of two glutarate catabolic pathways by assessing the ratio of five labeled carbons (*m* + 5) isotopolog of l‐2‐HG and citrate in *P. putida* KT2440, *P. putida* KT2440 (Δ*csiD*), and *P. putida* KT2440 (Δ*gcdH*). As shown in Figure 4J, the glutarate hydroxylation pathway mediated by CsiD contributed 93.38% of overall flux, while the glutaryl‐CoA dehydrogenation pathway involving GcdH contributed merely 6.62% (Figure 4K). This result was consisted with our previous observation that blocking the glutarate hydroxylation pathway would induce a more severe decrease in the growth rate of *P. putida* KT2440 with glutarate.^[^ ^24^ ^]^

### 2.5 Characterizing Glutarate Transport in Living Bacteria by using Glusor

**Figure 5:**
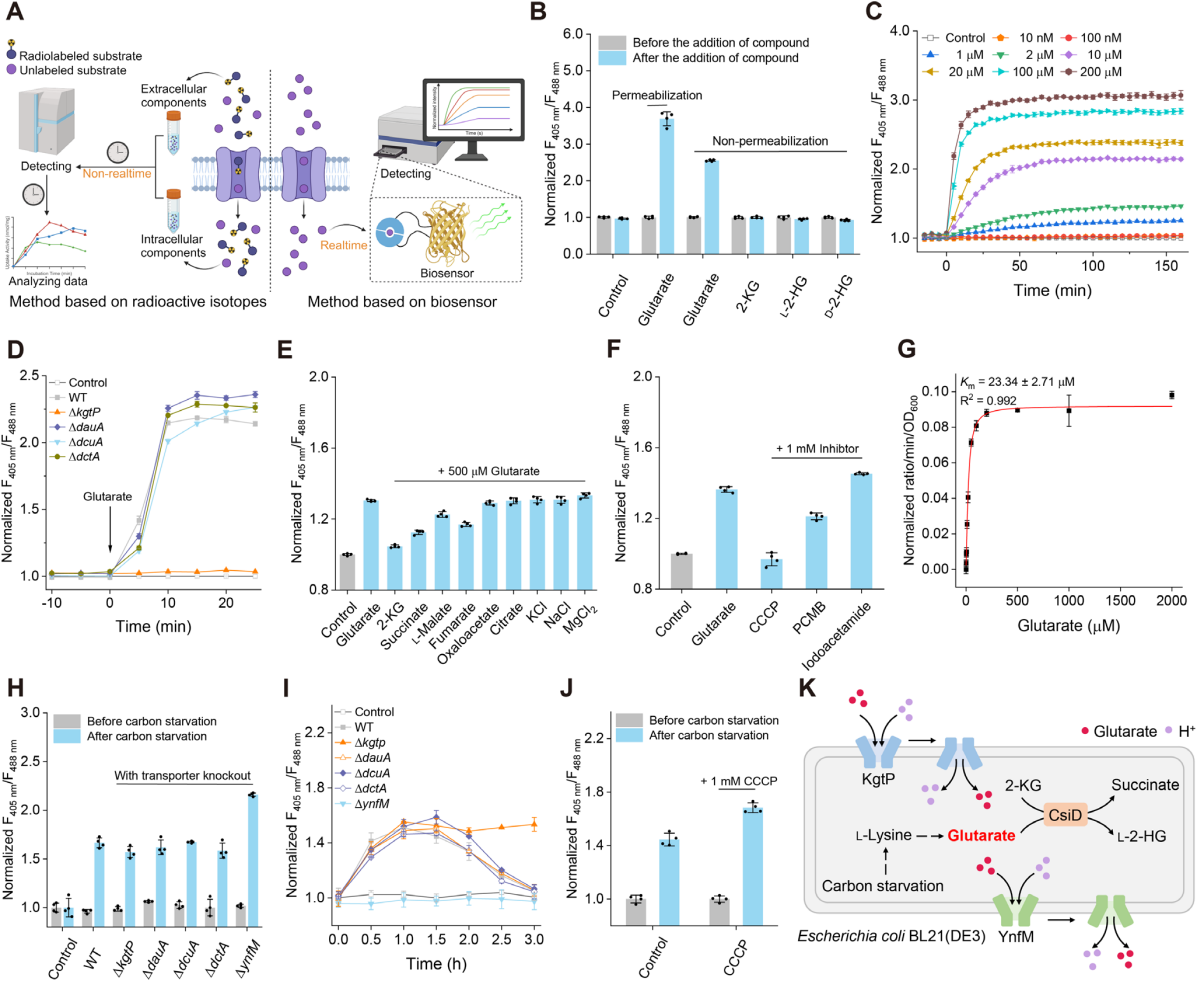
Characterizing glutarate transport in living bacteria using Glusor. A) Schematic representation of methods based on radioactive substrates or genetically encoded fluorescent biosensors for characterizing metabolites transport. The figure was generated using BioRender. B) Fluorescence ratio changes of Glusor expressed in *E. coli* BL21(DE3) before and after the addition of 200 µm glutarate, 2‐KG, l‐2‐HG, or d‐2‐HG. Data were corrected by cpSFYFP and normalized to the control (the ratio without any compounds). C) Time‐course analysis of fluorescence ratio changes of Glusor expressed in *E. coli* BL21(DE3) in response to exogenous glutarate addition. Glutarate at the indicated concentrations was added at time point zero. Data were corrected by cpSFYFP and normalized to the control without glutarate. D) Identification of the transporter related to glutarate uptake in *E. coli* BL21(DE3) by using Glusor. Data were corrected by cpSFYFP and normalized to the control (the ratio in the absence of glutarate). E) Substrate spectrum analysis of KgtP in *E. coli* BL21(DE3) using Glusor. Data were corrected by cpSFYFP and normalized to the control (without any compounds). F) Investigation of the transport type of KgtP using Glusor. Data were corrected by cpSFYFP and normalized to the control (ratio in the absence of glutarate and without any inhibitors). G) Kinetic analysis of KgtP‐mediated glutarate uptake. H) Identification of the transporter related to glutarate efflux in *E. coli* BL21(DE3) by using Glusor. Data were corrected by cpSFYFP and normalized to the control (ratio in MSM with 10 mm glucose). I) Investigation of functions of YnfM and KgtP by detecting extracellular glutarate using Glusor. Data were corrected by cpSFYFP and normalized to the control (ratio in MSM with 10 mM glucose). J) Investigation of the transport type of YnfM using Glusor. Data were corrected by cpSFYFP and normalized to the control (ratio in MSM with 10 mm glucose and without any inhibitors). K) Transport and metabolic mechanism of glutarate in *E. coli* BL21(DE3). All data shown are means ± standard deviations (s.d.) (*n* ≥ 3 independent experiments).

Transporters mediate metabolites exchange across cells and the extracellular environments. Metabolites transport is traditionally characterized through inconvenient and non‐realtime processes depending on radioactive substrates. Genetically encoded fluorescent biosensors can immediately and reversibly respond to the changes of metabolites in cells (**Figure** **5**A). In this study, we tentatively used Glusor as an example to investigate metabolites transport with genetically encoded fluorescent biosensors. As shown in Figure 5B, Glusor in permeabilized cells of *E. coli* BL21(DE3) exhibited significant responses to glutarate addition, while no detectable response to other tested compounds was observed. In addition, the response of Glusor in *E. coli* BL21(DE3) to exogenous glutarate is dose‐dependent (Figure 5C), with a *K*_d_ value of 10.22 ± 0.89 µm (Figure S16, Supporting Information).

Endogenous l‐lysine is utilized with glutarate as the key intermediate during carbon starvation of *E. coli*.^[^ ^25^, ^37^ ^]^ CsiD, the key enzyme in the glutarate hydroxylation pathway, is also present in *E. coli* BL21(DE3).^[^ ^25^, ^37^ ^]^ As expected, an increase in fluorescence emission ratio of Glusor was detected at the beginning of carbon starvation, and then gradually decreased due to CsiD mediated glutarate catabolism (Figure S17, Supporting Information). N‐oxalylglycine (NOG) is a reported inhibitor of 2‐KG‐dependent dioxygenases.^[^ ^38^ ^]^ Just like the situation of *csiD* deletion, NOG addition also obviously decreased the degradation of intracellular glutarate accumulated under carbon starvation (Figure S18, Supporting Information).

After verifying the feasibility of Glusor to monitor glutarate fluctuation in living bacteria, we applied Glusor in studying the transport of glutarate in *E. coli* BL21(DE3). As shown in Figure 5B, the Glusor in non‐permeabilized cells also respond to exogenous glutarate addition, indicating the existence of a transporter responsible for uptake of glutarate in *E. coli* BL21(DE3). The encoding genes of four dicarboxylate transporters including 2‐ketoglutarate permease (KgtP), dicarboxylic acid uptake system A (DauA), C_4_‐dicarboxylate transporter DcuA (DcuA), C_4_‐dicarboxylate transporter DctA (DctA) in *E. coli* were separately knocked out. As shown in Figure 5D, Glusor expressed in *E. coli* BL21(DE3) (Δ*kgtP*) did not respond to the exogenous addition of glutarate, confirming the role of KgtP in the uptake of glutarate.

Then, the uptake of glutarate by KgtP was detailedly characterized by using Glusor. As shown in Figure 5E, 2‐KG, succinate, l‐malate, and fumarate inhibited the transport of glutarate, indicating that these dicarboxylic acids may also be the substrate of KgtP. Among the proton gradient uncoupler carbonyl cyanide m‐chlorophenyl hydrazine (CCCP), the thiol inhibitor p‐Chloromercuribenzoate (PCMB), and the metabolic inhibitor iodoacetamide, only CCCP completely suppressed the glutarate uptake by KgtP (Figure 5F). This result suggested that KgtP may function as a proton‐coupled transporter. Kinetic parameter of KgtP was also determined by Glusor, and the estimated *K*_m_ value of KgtP for glutarate was calculated to be 23.34 µm (Figure S19, Supporting Information; Figure 5G).

We also assessed the feasibility of using a genetically encoded fluorescent biosensor to investigate metabolites efflux. Glusor was applied to detect intracellular and extracellular glutarate level induced by carbon starvation in different dicarboxylate transporter gene knockout strains. As expected, only Glusor expressed in *E. coli* BL21(DE3) (Δ*ynfM*) exhibited an obviously increased fluorescence ratio (Figure 5H). Extracellular accumulation of glutarate was undetectable during the carbon starvation of *E. coli* BL21(DE3) (Δ*ynfM*), indicating that YnfM may be the only functional glutarate exporter in *E. coli* BL21(DE3) (Figure 5I). YnfM belongs to the Major Facilitator Superfamily (MFS). CCCP addition increased intracellular glutarate accumulation of *E. coli* BL21(DE3) during carbon starvation (Figure 5J), suggesting that YnfM may also function as a proton‐coupled transporter. Finally, we propose a model of the glutarate transport and metabolism in *E. coli* BL21(DE3) via Glusor supported in vivo glutarate detection (Figure 5K).

### 2.6 Visualization of Glutarate Metabolism in HEK293FT Cells by using Glusor

**Figure 6:**
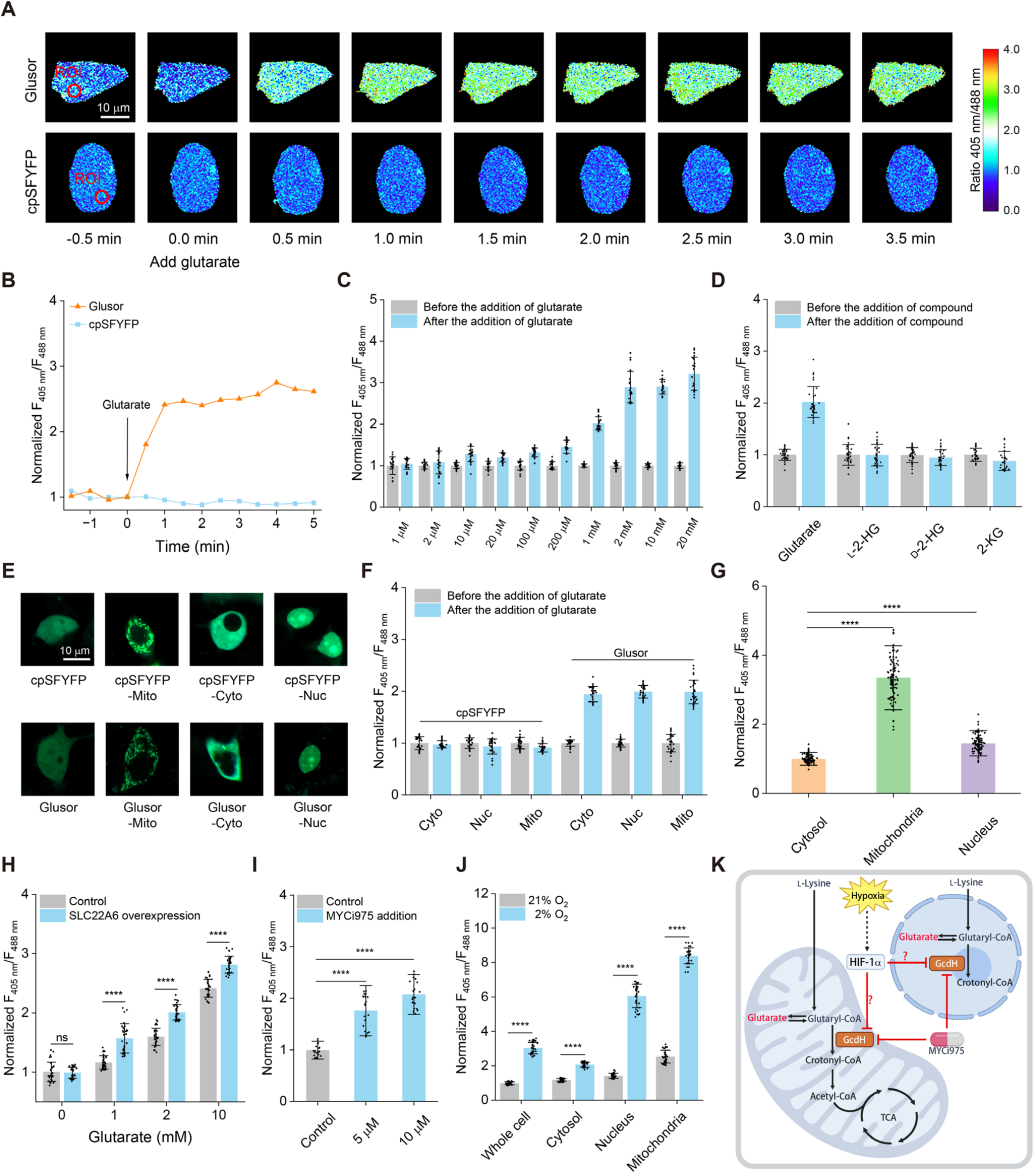
Visualization of glutarate fluctuations in living HEK293FT cells using Glusor. AB) Sequential images (A) and quantitative data (B) of Glusor and cpSFYFP expressed in HEK293FT cells in response to 2 mm glutarate addition. Scale bar, 10 µm. C) Dose‐dependent response of Glusor for increasing concentrations of glutarate (*n* = 20 cells). D) Specificity analysis of Glusor expressed in HEK293FT cells. The fluorescence ratios of Glusor before (grey) and after (blue) addition of 1 mm indicated metabolites were recorded (*n* = 30 cells). E) Fluorescence imaging of a single HEK293FT cell expressing subcellular localized cpSFYFP or Glusor. Scale bar, 10 µm. F) Response of different subcellular localized Glusor or cpSFYFP to exogenous glutarate addition. The ratios of Glusor and cpSFYFP before (grey) and after (blue) addition of glutarate were recorded (*n* = 30 cells). G) Subcellular distribution of glutarate determined by Glusor (*n* = 95 cells). H) Effect of SLC22A6 overexpression on glutarate uptake of HEK293FT cells. The ratios of Glusor in control cells (grey) and SLC22A6 overexpressed cells (blue) after addition of the indicated concentrations of glutarate were recorded (*n* = 60 cells). I) Effect of MYCi975 on glutarate accumulation in HEK293FT cells (*n* = 20 cells). J) Detection of hypoxia‐induced glutarate accumulation in HEK293FT cells. The fluorescence ratios were recorded after culturing Glusor‐expressing HEK293FT cells under normoxic (21% O_2_) or hypoxic (2% O_2_) conditions (*n* = 30 cells). K) Schematic representation of the role of GcdH in glutarate metabolism in HEK293FT cells. The figure was generated using BioRender. For (C), (D), and (F), data were normalized to the initial ratio (C), the ratio without any metabolites (D), and the ratio before glutarate addition (F), respectively. For (G‐J), data were corrected by cpSFYFP and normalized to the ratio of Glusor‐Cyto (G), the ratio of control in the absence of glutarate (H), the ratio of control (I), and the ratio of Glusor expressed in HEK293FT cells under normoxic conditions (J), respectively. All data shown are means ± standard deviations (s.d.) (*n* ≥ 3 independent experiments). ^*^ *p* < 0.05; ^**^ *p* < 0.01; ^***^ *p* < 0.001; ^****^ *p* < 0.0001; ns, no significant difference (*p* ≥ 0.05) in the two‐tailed *t*‐test.

Finally, we used Glusor for detecting intracellular glutarate in human cells. We overexpressed Glusor and cpSFYFP in HEK293FT cells and added 80 µm digitonin to permeabilize the HEK293FT cell membrane and eliminate endogenous glutarate. The addition of 2 mm glutarate in the imaging medium containing digitonin‐permeabilized HEK293FT cells induced an increased fluorescence ratio of Glusor, while cpSFYFP responded negligibly to the addition of glutarate (**Figure** **6**A,B). The Glusor expressed within HEK293FT cells also exhibited a dose‐dependent response to glutarate (Figure 6C; Figure S20, Supporting Information) and its *K*_d_ for glutarate was calculated to be 1.20 ± 0.53 mm (Figure S21, Supporting Information). Addition of structural analogs of glutarate, including l‐2‐HG, d‐2‐HG, and 2‐KG, had no effect on the response of Glusor to glutarate, confirming the specificity of Glusor in human cells (Figure 6D; Figure S22, Supporting Information).

The Glusor without localization sequences was uniformly distributed throughout the HEK293FT cells (Figure 6E). We fused the sequences for cytosolic localization, mitochondrial localization, and nuclear localization to the N‐, N‐, and C‐terminus of Glusor to facilitate its subcellular positioning in HEK293FT cells. As shown in Figure 6E, the fluorescence of Glusor was effectively confined to the mitochondria, cytosol, and nucleus by fusion with appropriate localization sequences. Subsequently, 1 mm glutarate was added to the imaging medium of digitonin‐permeabilized HEK293FT cells and an identical response of Glusor in various subcellular compartments was observed (Figure 6F). GcdH, the key enzyme required glutarate catabolism in human cells, is localized in mitochondria and the nucleus.^[^ ^39^, ^40^ ^]^ We also investigated the subcellular distribution of glutarate by comparing the fluorescence ratios of differently localized Glusor in HEK293FT cells. Higher concentrations of glutarate were observed in mitochondria and the nucleus (Figure 6G), which is consistent with the primary localization of GcdH in these compartments. Organic anion transporter 1 (OAT1), also known as SLC22A6, was reported to function as a transporter for the uptake of glutarate in human cells.^[^ ^41^, ^42^, ^43^ ^]^ We overexpressed both SLC22A6 and Glusor in HEK293FT cells and assayed the response of the constructed cells to exogenous glutarate. Due to the glutarate transport capability of SLC22A6, its overexpression significantly increased the response of Glusor in HEK293FT cells to the addition of glutarate (Figure 6H).

Genomic sequencing analysis revealed the relation between mutations in the *gcdH* gene and the occurrence of glutaric aciduria type I (GAI).^[^ ^44^ ^]^ MYCi975 is a small molecule inhibitor suppressing the expression of GcdH.^[^ ^39^, ^45^ ^]^ As expected, addition of MYCi975 may decrease the glutarate catabolism through GcdH and resulted in elevated levels of glutarate within HEK293FT cells (Figure 6I). Hypoxia is known to repress the degradation of hypoxia‐inducible factor 1α (HIF‐1α).^[^ ^46^ ^]^ HIF‐1α may decrease the expression of GcdH and then affect glutarate catabolism.^[^ ^11^, ^12^ ^]^ The HEK293FT cells with overexpressed Glusor or cpSFYFP were cultured under normoxic or hypoxic conditions. As illustrated in Figure 6J, an increase of the emission ratio of Glusor in various subcellular compartments of HEK293FT cells was observed under hypoxic condition. Thus, GcdH may be the crucial enzyme in glutarate catabolism of HEK293FT cells. Decrease of GcdH expression induced by MYCi975 addition or hypoxia impaired the catabolism of glutarate and resulted in accumulation of intracellular glutarate (Figure 6K).

## 3 Discussion

Glutaric aciduria is an autosomal recessive inherited metabolic disorder.^[^ ^9^ ^]^ Early diagnosis through implementing newborn screening (NBS) is essential for the management of this disease.^[^ ^47^ ^]^ Glutarate concentration in body fluids is a diagnostic marker with sensitivity greater than 95% for NBS of glutaric aciduria.^[^ ^48^ ^]^ The glutarate detection based on time‐consuming MS‐based methods is rather expensive for high‐throughput NBS and long‐term management of glutaric aciduria. The LODs of Glusor for glutarate in urine and serum are 1.62 and 0.21 µm, respectively (Figure 4D–E; Figure S14C,D, Supporting Information). The concentrations of glutarate in urine of the patients with glutaric aciduria often exceed 100 µm (normal range: 0–8.44 µm).^[^ ^49^ ^]^ Thus, Glusor is rather suitable for the measurement of glutarate in body fluids of patients with glutaric aciduria. Compared with the time‐consuming and costly MS‐based detection, the quantification of glutarate through Glusor is more cost‐effective and Glusor may serve as a potential choice for the quick NBS of glutaric aciduria (Table S2, Supporting Information). Wearable biosensors for biochemical markers monitoring can assist better management of metabolic diseases.^[^ ^50^, ^51^, ^52^ ^]^ For example, many microneedle‐based wearable glucose biosensors have been developed for personalized diabetes management.^[^ ^53^, ^54^, ^55^, ^56^ ^]^ Glusor may also be integrated into a microneedle‐based fluorometric sensing system for improved management of glutaric aciduria.

Glutarate is also an important dicarboxylate with diverse applications.^[^ ^27^ ^]^ Based on the glutarate detection by Glusor and metabolic flux analysis, we found that the glutarate hydroxylation pathway involved with CsiD dominates the glutarate catabolism in *P. putida* KT2440 (Figure 4I–K). However, blocking both the hydroxylation pathway and glutaryl‐CoA dehydrogenation pathway is required for efficient accumulation of glutarate.^[^ ^24^ ^]^ Although the glutaryl‐CoA dehydrogenation pathway is absent, the glutarate hydroxylation pathway is present in *E. coli*.^[^ ^25^ ^]^ Inactivation of the hydroxylation pathway might be a useful strategy to enhance glutarate production by recombinant *E. coli*. During the analysis of the binding pattern between CsiR and glutarate, we observed that CsiR‐LBD^Y84A^ exhibited an obviously decreased response to glutarate (Figure 1I). Interestingly, the Glusor variant with the same mutation has an increased *K*_d_ value of 2.62 mm (Figure S12B, Supporting Information). This Glusor variant may also be employed in the screening of high‐glutarate‐producing strains or associated enzymes.

Enhancing product efflux and blocking product uptake may reduce the feedback inhibition and increase the yield of the target chemical. However, the transporter for the uptake of glutarate has never been characterized in microorganisms. Substrates labeled with radioactive isotopes are often required for studying the function of metabolites transporters.^[^ ^57^ ^]^ In this study, we identified the role of KgtP and YnfM in glutarate transport of *E. coli* and characterized KgtP through Glusor‐enabled in vivo glutarate detection. Homologs of KgtP are distributed in many microorganisms utilized for glutarate production, like *P. putida* and *C. glutamicum* (Figure S23, Supporting Information). The *K*_m_ value for glutarate, the substrate spectrum, and transport mechanism of KgtP in *E. coli* can be easily affirmed by using Glusor. Besides Glusor, many genetically encoded metabolites biosensors which allow the rapid, specific, and real‐time monitor of metabolite dynamics in living cells have been developed.^[^ ^20^, ^35^ ^]^ These biosensors may also be used in studying the transporters for different metabolites.

Homologs of CsiD could only be found in bacteria,^[^ ^24^ ^]^ and thus the glutaryl‐CoA dehydrogenation pathway mediated by GcdH is indispensable for glutarate catabolism in human cells. GcdH is localized in mitochondria and the nucleus,^[^ ^39^ ^]^ but the compartmental distribution of glutarate is previously unclear. In this study, we investigated the subcellular distribution of glutarate by using differently localized Glusor, and detected higher concentrations of glutarate in mitochondria and the nucleus of HEK293FT cells (Figure 6G). Mutant in GcdH blocked glutarate catabolism and resulted in high concentrations of glutarate in body fluids and tissues of patients with GAI.^[^ ^58^ ^]^ Glutarate may be secreted into body fluids through organic anion transporter 4 (OAT4) and taken up from the plasma by OAT1 and sodium‐dicarboxylate cotransporter (NaDC3).^[^ ^42^, ^43^ ^]^ We verified the glutarate uptaking function of OAT1 by using Glusor (Figure 6H). The transporters involved in glutarate efflux or uptaking may be potential therapeutic targets for alleviating glutarate metabolism disorders. Glusor can be utilized to validate the functions of other glutarate transporters and screen candidate drugs toward glutarate transport.

Besides a toxic chemical inducing glutaric aciduria, glutarate is also an important metabolite involved in anti‐tumor immunity. For example, glutarate can regulate the metabolism of T cell and modulate its anti‐tumor function.^[^ ^12^ ^]^ Inhibiting the expressing of GcdH by using MYCi975 or under hypoxic condition will help T cells to generate more glutarate, alter the differentiation of T cells, and increase T cells cytotoxicity.^[^ ^12^ ^]^ We identified the potential of Glusor in real‐time monitoring of intracellular glutarate, and verified MYCi975 and hypoxia induced glutarate accumulation in HEK293FT cells. These results are consistent with those obtained using complex methods like LC‐tandem.^[^ ^12^ ^]^ Glusor may also be introduced into other cells and help to better understand the metabolism mechanisms and functions of glutarate in different cell types.

In summary, we constructed a high performance genetically encoded glutarate biosensor Glusor with excellent specificity, high response magnitude, swift response speed, and good reversibility. A fast, accurate, and low‐cost procedure for detecting glutarate in different biological samples was developed. The potential of Glusor for real‐time and in situ assays of intracellular glutarate was also demonstrated by using *E. coli* BL21(DE3) and HEK293FT cells. In addition, we used Glusor to identify and characterize the transporter responsible for glutarate uptake and efflux in *E. coli* BL21(DE3), expanding the applications of genetically encoded biosensors. We anticipate that Glusor may serve as a valuable tool in the diagnosis of glutarate‐related diseases, the construction of efficient glutarate producing strains, and illuminating the metabolic mechanisms and physiological functions of glutarate.

## 4 Experimental Section

### Chemicals and Reagents

Glutarate, 2‐KG, l‐2‐HG, d‐2‐HG, succinate, fumarate, l‐lactate, d‐lactate, oxaloacetate, l‐malate, d‐malate, cis‐aconitate, isocitrate, citrate, pyruvate, l‐glutamate, l‐lysine, glucose, SYPRO Orange, bovine serum albumin (BSA), and NOG were purchased from Sigma–Aldrich (USA) in this study. ^13^C_6_‐l‐lysine was purchased from Cambridge Isotope Laboratories, Inc (USA). Human serum was purchased from Beijing Solarbio Science and Technology Co., Ltd (China). Urine samples were collected from a healthy volunteer. MYCi975, CCCP, PCMB, and iodoacetamide were purchased from Shanghai Macklin Biochemical Technology Co., Ltd (China).

### Bacterial Strains and Culture Conditions

The bacterial strains relevant to this study are listed in Table S3 (Supporting Information). *E. coli* BL21(DE3) and its derivatives were cultivated in Luria‐Bertani (LB) medium at 37 °C and 180 rpm. *P. putida* KT2440 and its derivatives were cultivated in minimal salt medium (MSM) containing various carbon sources at 30 °C and 200 rpm. Antibiotics were used at the following concentrations: ampicillin at 100 µg mL^−1^, kanamycin at 50 µg mL^−1^, spectinomycin at 50 µg mL^−1^.

### Cell Lines and Culture Conditions

HEK293FT cells were cultured in high‐glucose Dulbecco's Modified Eagle Medium (DMEM, ThermoFisher, USA) supplemented with 10% (vol/vol) fetal bovine serum (FBS, Biological Industries, Israel) and 1% (vol/vol) penicillin‐streptomycin (ThermoFisher, USA) at 37 °C and 5% CO_2_. For hypoxic experiments, HEK293FT cells were cultured in a BioSpherix ProOx C21 subchamber controller (BioSpherix, USA) at 2% O_2_.^[^ ^31^ ^]^

### Expression and Purification of CsiR, CsiR‐LBD, and Mutants of CsiR‐LBD

For expression and purification of CsiR, the *csiR* gene was amplified using primer pair CsiR‐F2/R2 and cloned into pETDuet‐1 plasmid using T5 exonuclease DNA assembly (TEDA) technique to construct pETDuet‐CsiR.^[^ ^59^ ^]^ *E. coli* BL21(DE3) strain carrying pETDuet‐CsiR plasmid was cultivated in LB medium at 37 °C and 180 rpm until the OD_600_ (optical density at 600 nm) reached 0.6. Then, 1 mm isopropyl β‐d‐1‐thiogalactopyranoside (IPTG) was added to induce CsiR expression for 12 h at 16 °C and 160 rpm. The cultures were collected by centrifugation at 4000 × *g* for 10 min at 4 °C, washed twice with phosphate‐buffered saline (PBS), resuspended in Buffer A (20 mm sodium phosphate, 500 mm NaCl, and 20 mm imidazole, pH 7.4) containing 1 mm phenylmethylsulfonyl fluoride (PMSF) and 10% (vol/vol) glycerol, and lysed using an AH‐BASIC high‐pressure homogenizer (Antuos Nanotechnology, China) at 4 °C. The lysate was centrifuged at 13000 × *g* for 50 min at 4 °C, and the obtained supernatant was then loaded onto a 5 mL HisTrap HP column (GE Healthcare, USA) pre‐equilibrated with Buffer A. The CsiR protein was eluted using a gradient of Buffer B (20 mm sodium phosphate, 500 mm NaCl, and 500 mm imidazole, pH 7.4). Purified CsiR protein was analyzed by 13% sodium dodecyl sulfate‐polyacrylamide gel electrophoresis (SDS‐PAGE) and quantified using the Bradford protein assay kit (Sangon, China). Expression and purification of CsiR‐LBD and its mutants were performed using the same procedure.

### Fluorescence‐Based Thermal Shift (FTS) Assay

For FTS assays, 10 µm purified CsiR or CsiR‐LBD, 100 µm tested compounds, and 5× SYPRO Orange were mixed in 50 µL FTS buffer (20 mm sodium phosphate and 150 mm NaCl, pH 7.4). 25 µL of the mixture was then transferred into a 96‐well white PCR plate (Roche, Switzerland), and ligand binding‐induced conformational changes in CsiR or CsiR‐LBD were analyzed using a LightCycler 480 system (Roche, Switzerland) with the following instrument settings: temperature from 25 to 95 °C at 0.07 °C s^−1^, excitation at 465 nm, and emission at 580 nm. The Tm value was determined based on the derivative melt curves generated from raw fluorescence data at each temperature. Compounds with ΔTm values above 2 °C were considered to possess the ability to bind to CsiR or CsiR‐LBD.

### Gel Filtration Chromatography

Gel filtration chromatography was conducted with a Superdex 200 10/300 GL column (GE Healthcare, USA). The column was equilibrated with 50 mm sodium phosphate buffer (pH 7.2) containing 150 mm NaCl. The standards used were aprotinin (6.5 kDa), ovalbumin (43 kDa), conalbumin (75 kDa), aldolase (158 kDa), and ferritin (440 kDa).

### Structure Prediction and Molecular Docking

Structure prediction of CsiR monomer or dimeric CsiR and Glusor were performed using Alphafold2 or Alphafold3, respectively. The molecular docking of CsiR and Glusor in complex with glutarate were performed using Schrödinger Glide software (Schrödinger Release 2022‐1, USA) with default settings for all parameters. PyMOL software (DeLano Scientific LLC, USA) and PLIP online service (https://plip‐tool.biotec.tu‐dresden.de/plip‐web) were used to analyze the complex structures of CsiR or Glusor with glutarate, along with the related hydrogen bonds, hydrophobic interactions, and crucial amino acid residues.

### Molecular Dynamics Simulation

The glutarate‐free and glutarate‐bound structures of CsiR and Glusor obtained from molecular docking were loaded into Maestro (Schrödinger Release 2022‐1, USA) and optimized using the Protein Preparation Wizard module. The cubic box was solvated with the TIP3P water model, and the dimensions were set to 25 Å. The force field was OPLS2005 and 0.15 m NaCl was added into the system to simulate physiological ionic strength. Then, molecular dynamics simulations were performed in a workstation equipped with RTX3090. The interactions during the simulation process were evaluated using the Simulation Interactions Diagram module (Schrödinger Release 2022‐1, USA). Upon completion of the simulations, trajectory data were extracted and plotted using OriginPro 2023 software (OriginLab, USA).

### Construction and Optimization of Glutarate Biosensors—Insertion Sites Screening

The plasmids and primers used in this study are listed in Tables S3 and S4 (Supporting Information), respectively. To construct Glusor‐1 (a variant with cpSFYFP inserted between residues 225A/226E of CsiR), the pETDuet‐CsiR plasmid was linearized by inverse PCR using primer pair SF‐2‐225A/226E‐F/R, and the DNA fragment of cpSFYFP was PCR‐amplified using primer pair cpSFYFP‐F/R. The obtained cpSFYFP fragment and linearized pETDuet‐CsiR plasmid were then assembled using the TEDA method, and introduced into *E. coli* BL21(DE3) for further protein expression and purification. The construction of Glusor‐2 and other insertion mutants was performed using the same procedure.

### Construction and Optimization of Glutarate Biosensors—DNA‐Binding Domain Truncation

To construct Glusor‐3, the pETDuet‐Glusor‐2 plasmid was linearized by inverse PCR using primer pair SF‐15‐nodbd‐F1/R1, and the DNA fragment of Glusor‐3 was PCR‐amplified using primer pair SF‐15‐nodbd‐F2/R2 with pETDuet‐Glusor‐2 as the template. The obtained Glusor‐3 fragment and linearized pETDuet‐Glusor‐2 plasmid were then assembled using the TEDA method, and introduced into *E. coli* BL21(DE3) for further protein expression and purification.

### Construction and Optimization of Glutarate Biosensors—Linker Random Mutagenesis

To introduce a single‐site saturating mutation in the linker of Glusor‐3, the pETDuet‐Glusor‐3 plasmid was linearized by inverse PCR using primer pair pETDuet‐muta‐F1/R1, and the DNA fragment of cpSFYFP containing the N‐ and C‐terminal linkers was PCR‐amplified using primer pair which were designed to introduce the mutation (e.g., primer pair F2_S101_A/R2_GGC was used to introduce an alanine mutation at the first amino acid residue of the N‐terminal kinker). The obtained cpSFYFP fragment and linearized plasmid were then assembled using the TEDA method, and introduced into *E. coli* BL21(DE3). Crude extracts of different Glusor‐3 variants were prepared by lysis using a SCIENTZ‐48TD multi‐channel ultrasonic disrupter (Scientz Biotechnology, China) on ice and centrifugation at 13000 × *g* for 40 min at 4 °C. The prepared crude extracts were mixed with 1 mm glutarate in 96‐well plates and the response magnitude of different variants to glutarate was analyzed using an EnSight microplate reader (PerkinElmer, USA). Then, combinatorial mutations were performed using the same procedure for amino acids dominant in the N‐terminal linker and C‐terminal linker (response magnitude higher than 50%), and subjected to the next round of screening. The mutant with the highest response magnitude was finally purified and its dose‐response curve for glutarate was determined.

### Construction and Expression of Glusor Mutants Related to Glutarate Binding

To introduce a glutarate binding related mutation in Glusor, the mutant of pETDuet‐CsiR‐LBD plasmid was linearized by inverse PCR using primer pair Glusor‐muta‐F1/R1 (e.g., plasmid pETDuet‐CsiR‐LBD^Y84A^ was linearized to introduce an alanine mutation at the site of Y84), and the DNA fragment of cpSFYFP containing the N‐ and C‐terminal linkers of Glusor was PCR‐amplified using primer pair Glusor‐muta‐F2/R2. Expression and purification of Glusor mutants were performed using the same procedure.

### Characterization of Glusor In Vitro—Dose‐Response Curve Determination

Purified Glusor was diluted to 1.33 µm using 50 mm Tris‐HCl (pH 7.4) and mixed with increasing concentrations of glutarate in a black 96‐well plate at a volume ratio of 3:1 (total 100 µL per well). Changes in fluorescence intensities of Glusor were measured using an EnSight microplate reader (PerkinElmer, USA) with the following instrument settings: excitation at 405 or 488 nm, emission at 528 nm. The dose‐response curve was fitted using OriginPro 2023 software (OriginLab, USA) with the following equation:

(1) $$R=R_{\max}+\frac{R_{\min}-R_{\max}}{1+{\left(\left[\mathrm{Glutarate}\right]/K_{d}\right)}^{p}}$$

where *R*, *R*_min_, *R*_max_, [Glutarate], *K*_d_, and *p* represented experimental fluorescence ratio (F_405 nm_/F_488 nm_), minimum F_405 nm_/F_488 nm_, maximum F_405 nm_/F_488 nm_, glutarate concentration, apparent dissociation constant, and Hill slope, respectively. The maximum ratio change (Δ*R*_max_) was calculated using the following equation:

(2) $$\Delta R_{\max}=\left(R_{\max}-R_{\min}\right)/R_{\min}$$

### Characterization of Glusor In Vitro—Spectral Properties Analysis

Fluorescence spectrum of Glusor were analyzed using an EnSight microplate reader. For excitation spectrum, 530 nm emission was excited from 350 to 515 nm in steps of 1 nm. For the emission spectrum, 505 to 650 nm emission was detected in steps of 1 nm at 405 or 488 nm excitation.

### Characterization of Glusor In Vitro—Specificity Analysis

Purified Glusor was mixed with 100 µm different glutarate analogs (l‐2‐HG, d‐2‐HG, 2‐KG), compounds from the TCA cycle, l‐lactate, d‐lactate, l‐lysine, and glucose, respectively, and changes in fluorescence ratio were measured immediately. For the anti‐interference experiment, fluorescence responses of Glusor to 100 µm glutarate were detected in the presence of 100 µm different compounds.

### Characterization of Glusor In Vitro—Dynamic Response Analysis

Glusor at a concentration of 1 µm was added to a 96‐well plate with a volume of 27 µL per well at the beginning of the assay. Glutarate solution with the concentration of 1 mm, 200 µm, or 100 µm was added to per well with a volume of 3 µL to make the final glutarate concentrations to 100, 20, or 10 µm. The response of Glusor to added glutarate was continuously recorded by the fluorescence microplate reader for 60 s. Then, 50 mM Tris‐HCl buffer with a suitable volume was added in specific wells to dilute glutarate in the detection system to 10 µm, and the response of Glusor to glutarate dilution was continuously recorded by the fluorescence microplate for another 60 s.

### Characterization of Glusor In Vitro—Stability Analysis

For temperature‐stability analysis, changes in fluorescence ratio of Glusor to 0 or 100 µm glutarate were measured at a gradient of temperature (25–46 °C). For pH‐stability analysis, dose‐response curves of Glusor for increasing concentrations of glutarate were determined at a gradient of pH values (6.6–8.2). pH correction of Glusor by cpSFYFP was realized by dividing the fluorescence ratio of Glusor over the fluorescence ratio of cpSFYFP.

### Characterization of Glusor In Vitro—Reversibility Analysis

Purified Glusor was first mixed with 0 or 100 µm glutarate and then centrifuged at 3500 × *g* for 20 min at 4 °C using 10 kDa ultrafiltration centrifuge tubes to elute glutarate. The reversibility of Glusor was analyzed by detecting the changes in fluorescence ratio before and after glutarate elution.

### Determination of Glutarate in Different Biological Samples using Glusor

Different concentrations of glutarate were added into DMEM, MSM, human serum, and urine, respectively, and filtered through a 0.22 µm filter. Prepared samples were then mixed with diluted Glusor in a black 384‐well plate at a volume ratio of 3:1 (total 20 µL per well). Changes in fluorescence ratios were measured using an EnSight microplate reader. Absolute quantification of glutarate in these samples was achieved by substituting the measured Glusor fluorescence ratio into the formula of the dose response curve.

### Determination of Glutarate in Different Biological Samples using HPLC

For the determination of glutarate in DMEM, MSM, human serum, and urine using HPLC, the samples were heated at 105 °C for 15 min, centrifuged at 13 000 × *g* for 15 min to remove protein, filtered through a 0.22 µm filter, and then analyzed using an HPLC system (LC‐2050 series, Shimadzu, Japan) equipped with an Aminex HPX‐87H column (300 × 7.8 mm, Bio‐Rad, USA) and a refractive index detector. The mobile phase was 10 mm H_2_SO_4_ at a flow rate of 0.4 mL min^−1^. The column temperature was 55 °C, and the analysis duration was 35 min.^[^ ^24^ ^]^ The absolute concentrations of glutarate in the samples were calculated from a standard curve of glutarate.

### Metabolic Flux Analysis

Conversion of ^13^C_6_‐l‐lysine into ^13^C‐labeled glutarate was carried out by using whole cells of *P. putida* KT2440 (Δ*csiD*Δ*gcdH*Δ*alr*). *P. putida* KT2440, *P. putida* KT2440 (Δ*csiD*), and *P. putida* KT2440 (Δ*gcdH*) were cultured in MSM containing 2.5 g L^−1 13^C‐labeled glutarate and 2.5 g L^−1^ unlabeled glutarate to mid‐log phase (OD_600_ = 2.5). Then, the cultures were collected by centrifugation, quickly frozen in liquid nitrogen, and stored at −80 °C. Intracellular metabolites were extracted using −80 °C pre‐cooled methanol, dried using nitrogen gas, derivatized with *N*‐methyl‐*N*‐(*tert*‐ butyldimethylsilyl)‐trifluoroacetamide, and then subjected to GC‐MS detection. The mass isotopomer distributions (MIDs) of integral and fragmented metabolites were determined by analyzing the peak areas obtained by mass spectrometry and corrected for naturally occurring stable isotopes.^[^ ^60^ ^]^ The mass distribution vectors (MDV) of different pathway metabolites (l‐2‐HG and citrate) were chosen as the computational data. The least‐squares fitting was employed to determine the flux ratios of different pathways. All calculations were completed by using MATLAB 7.8.0 (MathWorks, USA).

### Construction of *E. coli* BL21(DE3) Mutants

Knock‐out of genes in *E. coli* BL21(DE3) were performed using the pEcCas‐pEcgRNA system‐mediated recombination.^[^ ^61^ ^]^ The sgRNA was designed using an online sever (http://chopchop.cbu.uib.no/) and expressed by the pEcgRNA plasmid. The knockout fragment was generated by PCR recombination of upstream and downstream fragments of the target gene. 1 µg knockout fragment and 250 ng pEcgRNA plasmid were transferred into *E. coli* BL21(DE3) expressing the pEcCas plasmid by electroporation. The pEcgRNA and pEcCas plasmids were then eliminated by successive cultivation in LB liquid with 10 mm rhamnose and LB plate with 10 g L^−1^ sucrose.

### Characterization of Glusor in Living Bacteria

*E. coli* BL21(DE3) harboring pETDuet‐Glusor or pETDuet‐cpSFYFP was cultivated in LB medium at 37 °C until OD_600_ reached 0.6. 1 mm IPTG was added to induce protein expression overnight at 16 °C. The cultures were harvested by centrifugation at 6000 × *g* for 5 min, washed twice, and resuspended to an OD_600_ of 5.0 by carbon starvation medium (MSM without any carbon source). Bacterial cells requiring permeabilization were harvested by centrifugation (6000 × *g*, 5 min, 4 °C) and subsequently resuspended in permeabilization buffer (carbon starvation medium supplemented with 0.1% [vol/vol] toluene) to an OD_600_ of 5.0. To analyze the specificity and gradient response of Glusor expressed in *E. coli* BL21 (DE3) to glutarate, 99 µL cell suspensions following 2 h carbon starvation were mixed with 1 µL increasing concentrations of glutarate or different glutarate analogs in a black 96‐well plate. Changes in fluorescence intensity were then measured every 5 min using an EnSight microplate reader with continuous shaking at 30 °C.

### Monitoring Carbon Starvation‐Induced Glutarate Accumulation

*E. coli* BL21(DE3) and *E. coli* BL21(DE3) (Δ*csiD*) expressing Glusor or cpSFYFP were suspended to an OD_600_ of 5.0 by carbon starvation medium (MSM without any carbon source) or glucose medium (MSM containing 10 mm glucose). 100 µL cell suspensions were then transferred into a black 96‐well plate, and changes in fluorescence intensity were measured every 5 min using an EnSight microplate reader with continuous shaking at 30 °C. To characterize the role of CsiD in glutarate catabolism during carbon starvation, the inhibitor of CsiD, NOG, was added to the detection system at a concentration of 10 µm at the beginning of the assay.

### Analysis of the Transport Mechanism of Glutarate in E. coli BL21 (DE3)

*E. coli* BL21(DE3), *E. coli* BL21(DE3) (Δ*kgtP*), *E. coli* BL21(DE3) (Δ*dctA*), *E. coli* BL21(DE3) (Δ*dauA*), *E. coli* BL21(DE3) (Δ*dcuA*) and *E. coli* BL21(DE3) (Δ*ynfM*) expressing Glusor or cpSFYFP were collected by centrifugation at 8000 × *g* for 1 min, washed twice, and then resuspended to an OD_600_ of 5.0 in carbon starvation medium. For analysis of kinetic parameters, substrate spectrum, and transport type of KgtP, the time interval of the assay was set to 1 min, and 1 µL of different glutarate analogs or different inhibitors were additionally added into the detection system. 99 µL cell suspensions following 2 h carbon starvation were mixed with 1 µL increasing concentrations of glutarate in a black 96‐well plate. Changes in fluorescence intensity were then measured every 5 min using an EnSight microplate reader with continuous shaking at 30 °C. For analysis of the function and transport type of YnfM, the time interval of detecting intracellular glutarate level was set to 20 min. For analysis of extracellular glutarate level, strains tested were collected by centrifugation at 8000 × *g* for 1 min, and resuspended immediately to an OD_600_ of 30 in carbon starvation medium with 100 µm purified Glusor or cpsfYFP.

### Expression of Glusor in HEK293FT Cells

The mammalian codon‐optimized sequence of Glusor and cpSFYFP were synthesized by General Biosystems Co., Ltd (China) and cloned into pcDNA3.1^(+)^ plasmid prefixed with Kozak sequence (GCCACC). Transfection of Glusor in HEK293FT cells were carried out using the HieffTrans lipid‐based transfection reagent (Yeasen, China) according to the manufacturer's protocol. Briefly, an appropriate number of HEK293FT cells were plated in poly‐l‐lysine‐pretreated 35‐mm glass‐bottom dishes to achieve 70–90% confluence after 24 h. 1.5 µL HieffTrans lipid‐based transfection reagent (Yeasen, China) and 1 µg pcDNA3.1^(+)^ plasmid encoding Glusor or cpSFYFP were mixed in 50 µL Opti‐MEM Reduced Serum Medium (ThermoFisher, USA), incubated at room temperature for 15 min, and then added into the cultures. To facilitate proper folding of Glusor in HEK293FT cells, the culture temperature after transfection was adjusted to 30 °C. Imaging was typically conducted 24 h after transfection.

### Imaging of Glusor in HEK293FT Cells

Imaging of Glusor in HEK293FT Cells were performed using a Zeiss 900 confocal microscope (Zeiss, Germany) equipped with a 405 nm diode laser, a 488 nm argon laser, 20×/0.8 and 40×/0.95 objective lens, and a full‐spectrum fluorescence detector. To analyze the dose‐response curves and specificity of Glusor in HEK293FT cells, the transfected cells were first washed twice with HHBSS buffer (1× Hank's Balanced Salt Solution supplemented with 20 mm HEPES, ThermoFisher, USA). 80 µm digitonin was added to permeabilize cell membranes.^[^ ^36^ ^]^ Then, gradient concentrations of glutarate or different analogs were added separately and changes in fluorescence ratio were imaged by the following procedure: excitation at 405 and 488 nm, emission at 497–617 nm, 750 gain, 1024 × 1024 frame size, 8‐bit depth, and time intervals of 30 s. All data in the same experiment were collected using consistent imaging parameters and laser power settings. ZEN 3.4 software was used for random images acquisition. ImageJ software was used for ratiometric image analysis.

### Subcellular localization of Glusor

For mitochondrial localization of Glusor, a mitochondrial targeting sequence derived from yeast cytochrome c oxidase subunit IV (MLSLRQSIRFFKPATRTLCSSRYLL) was fused to the N‐terminus of Glusor. For cytosolic localization of Glusor, a tandem repeat of the MAPKK nuclear export signal (MALQKKLEELELDEQRKRLEDL)_2_ was fused to the N‐terminus of Glusor. For nuclear localization of Glusor, three repeats of the SV40 large T antigen nuclear localization signal (DPKKKRKV)_3_ were fused to the C‐terminus of Glusor. Expression and characterization of different subcellular localized Glusor in HEK293FT cells were achieved using consistent methods as described above.

### Application of Glusor for Glutarate Detection in HEK293FT Cells

For analysis of the function of SLC22A6 in glutarate transport, HEK293FT cells transfected with Glusor or co‐transfected with Glusor and SLC22A6 were treated with increasing concentrations of glutarate (0, 1, 2, and 10 mm) 2 h after transfection. Changes in the fluorescence ratio of Glusor were imaged 22 h after glutarate addition. For analysis of the function of GcdH in glutarate catabolism, HEK293FT cells transfected with Glusor were treated 2 h after transfection with 5 or 10 µm GcdH inhibitor, MYCi975, and changes in the fluorescence ratio of Glusor were imaged 22 h after MYCi975 addition. For analysis of hypoxia‐induced glutarate accumulation, HEK293FT cells expressing Glusor or different subcellular localized Glusor were cultured sequentially under normoxic conditions for 24 h and hypoxic conditions for 24 h, and then changes in the fluorescence ratio of Glusor were measured.

### Statistical Analysis and Reproducibility

Statistical analysis of initial data was carried out by Microsoft Excel 2019. Further data processing and analysis were executed using OriginPro 2023 (OriginLab, USA) and GraphPad Prism 9 (GraphPad, USA). The data obtained from the FTS assay were processed with LightCycler 480 software (Roche, USA). SDS‐PAGE gels were collected using CanoScan 9000 F MarKII scanner (Canon, Japan). Fluorescence intensity data detected by the Ensight microplate reader were obtained by Kaleido 3.0 (PerkinElmer, USA). The acquisition and processing of imaging data were achieved by Zen 3.4 (Zeiss, Germany) and ImageJ 1.5 (National Institutes of Health, USA), respectively. All data shown are means ± standard deviations. Significance difference analysis were conducted using unpaired two‐tailed *t*‐test where appropriate; ^*^ *p* < 0.05; ^**^ *p* < 0.01; ^***^ *p* < 0.001; ^****^ *p* < 0.0001; ns, not significant (*p* ≥ 0.05).

## Conflict of Interest

C.G., K.G., C.L., C.M., and P.X. are inventors of a pending patent application (Chinese patent application no. 2025109957238). The patent was submitted by Shandong University. Other authors declare no relevant conflicts of interest.

## Author Contributions

C.G., C.M., C.L., and P.X. designed the research. K.G., H.Z., Y.L., X.X., W.L., Z.K., R.X., S.H., and P.H. performed the research. K.G. and C.G. analyzed the data. K.G., C.G., C.M., and P.X. wrote the manuscript.

## Supporting information



Supporting Information

## Data Availability

The data that support the findings of this study are available from the corresponding author upon reasonable request.
