# Supplementary material for: A Genetically Encoded Biosensor for Characterizing Transport and Metabolism of Glutarate
Source: Adv Sci (Weinh). 2025 Aug 20;12(42):e07046. doi: 10.1002/advs.202507046 (PMC12622518; doi:10.1002/advs.202507046)
Supplement: Supplementary file 1 — Supporting Information [file ADVS-12-e07046-s001.docx]

**Supporting Information**

**A Genetically Encoded Biosensor for Characterizing Transport and Metabolism of Glutarate**

*Kaiyu Gao,^[a]^ Hui Zhang,^[a]^ Yidong Liu,^[a]^ Xianzhi Xu,^[a]^ Wei Liu,^[a]^ Zhaoqi Kang,^[a]^ Rong Xu,^[a]^ Shuang Hou,^[a]^ Ping Han,^[a]^ Chuanjuan Lü,^[a]^ Cuiqing Ma,^[a]^ Ping Xu,^[b]^ and Chao Gao^[a],^**

[a] K. Gao, H. Zhang, Y. Liu, X. Xu, W. Liu, Z. Kang, R. Xu, S. Hou, P. Han, C. Lü, C. Ma, C. Gao

State Key Laboratory of Microbial Technology, Shandong University, Qingdao, People’s Republic of China

*Corresponding author: C. Gao, E-mail: jieerbu@sdu.edu.cn

[b] P. Xu

State Key Laboratory of Microbial Metabolism, Joint International Research Laboratory of Metabolic & Developmental Sciences, and School of Life Sciences & Biotechnology, Shanghai Jiao Tong University, Shanghai, People’s Republic of China

E-mail: pingxu@sjtu.edu.cn


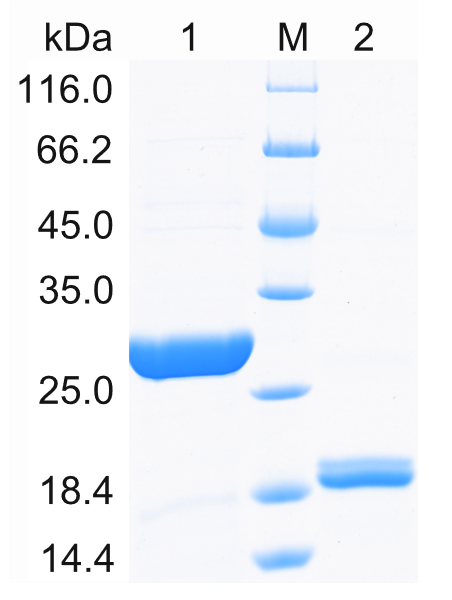


**Figure S1 SDS-PAGE analysis of the purification of CsiR and CsiR-LBD.** Lane M, molecular weight markers; lane 1, purified His_6_-tagged CsiR (27.5 kDa) using a HisTrap column; lane 2, purified His_6_-tagged CsiR-LBD (19.1 kDa) using a HisTrap column.


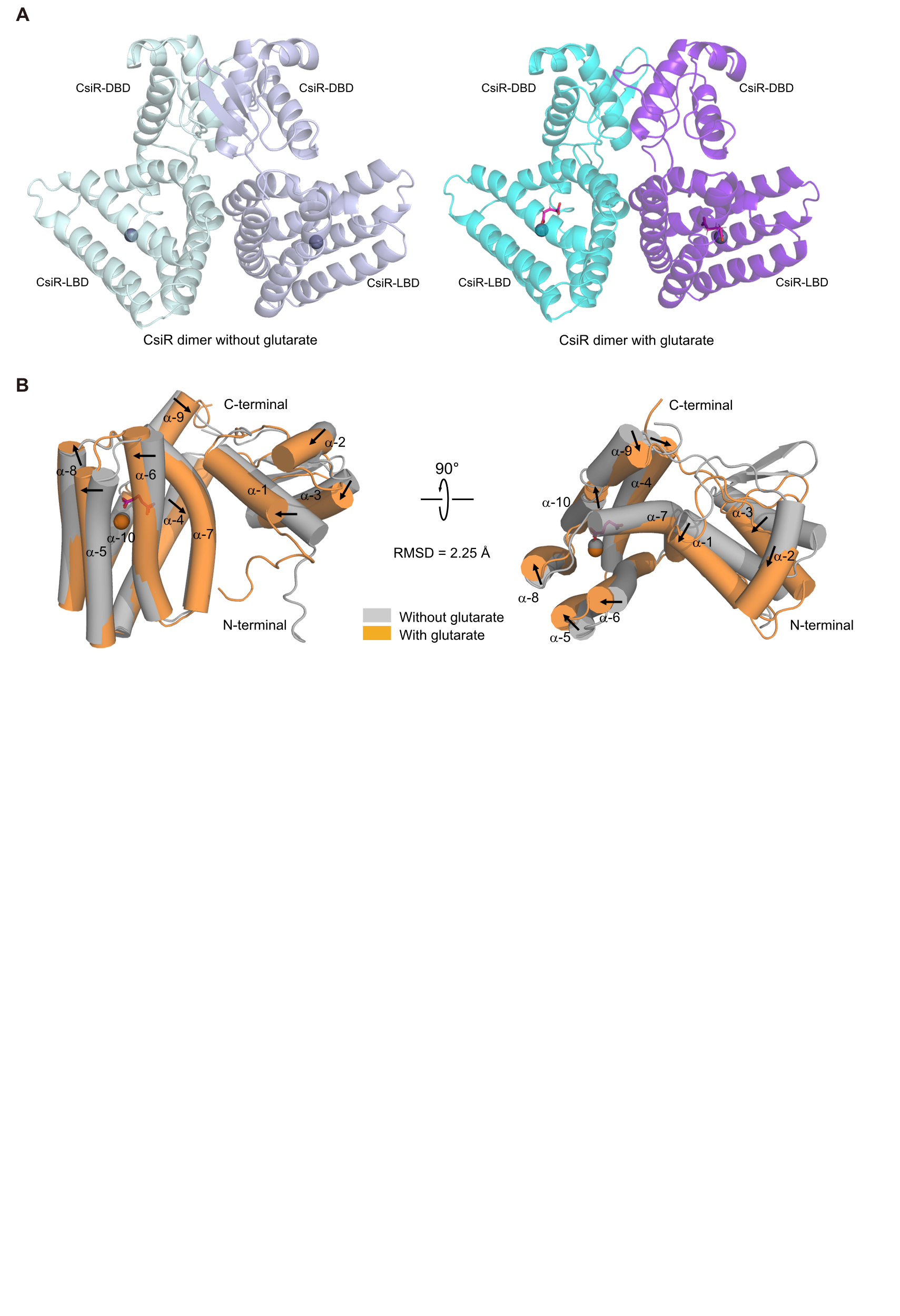


**Figure S2** **Molecular dynamics simulations of CsiR.** **(A)** Predicted structure of dimeric glutarate-free or glutarate-bound CsiR by molecular dynamics simulation. Glutarate was represented as magenta sticks. **(B)** Superposition of the dimeric CsiR in glutarate-free and glutarate-bound states. All helices are depicted as cylinders and clearly labeled. Regions with significant differences are marked with black arrows.


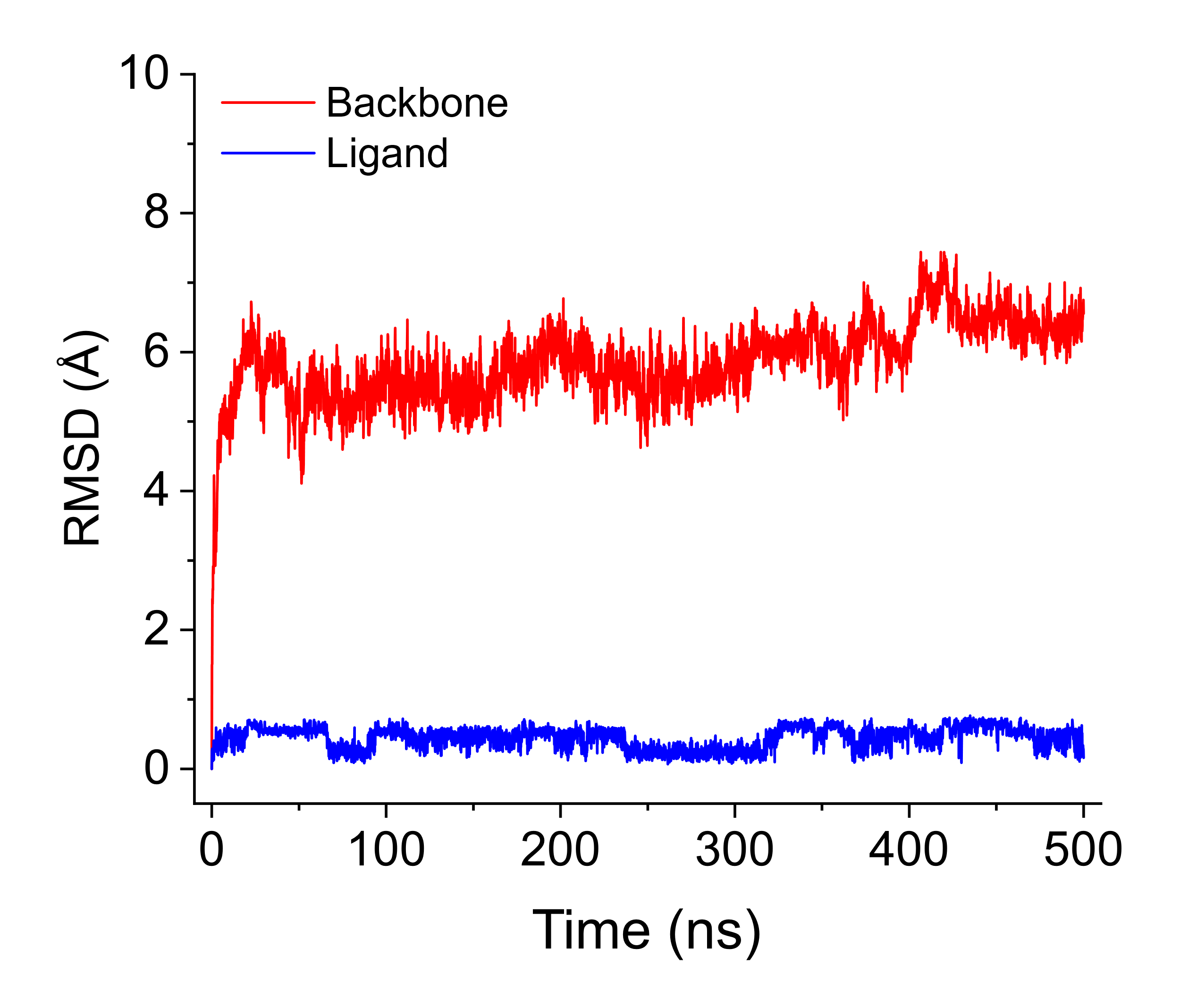


**Figure S3 Molecular dynamics simulations of CsiR with glutarate.** Red line, RMSD (root-mean-square deviation) changes of CsiR backbone carbon atoms during the simulation process. Blue line, RMSD changes of ligand (glutarate) atoms during the simulation process.


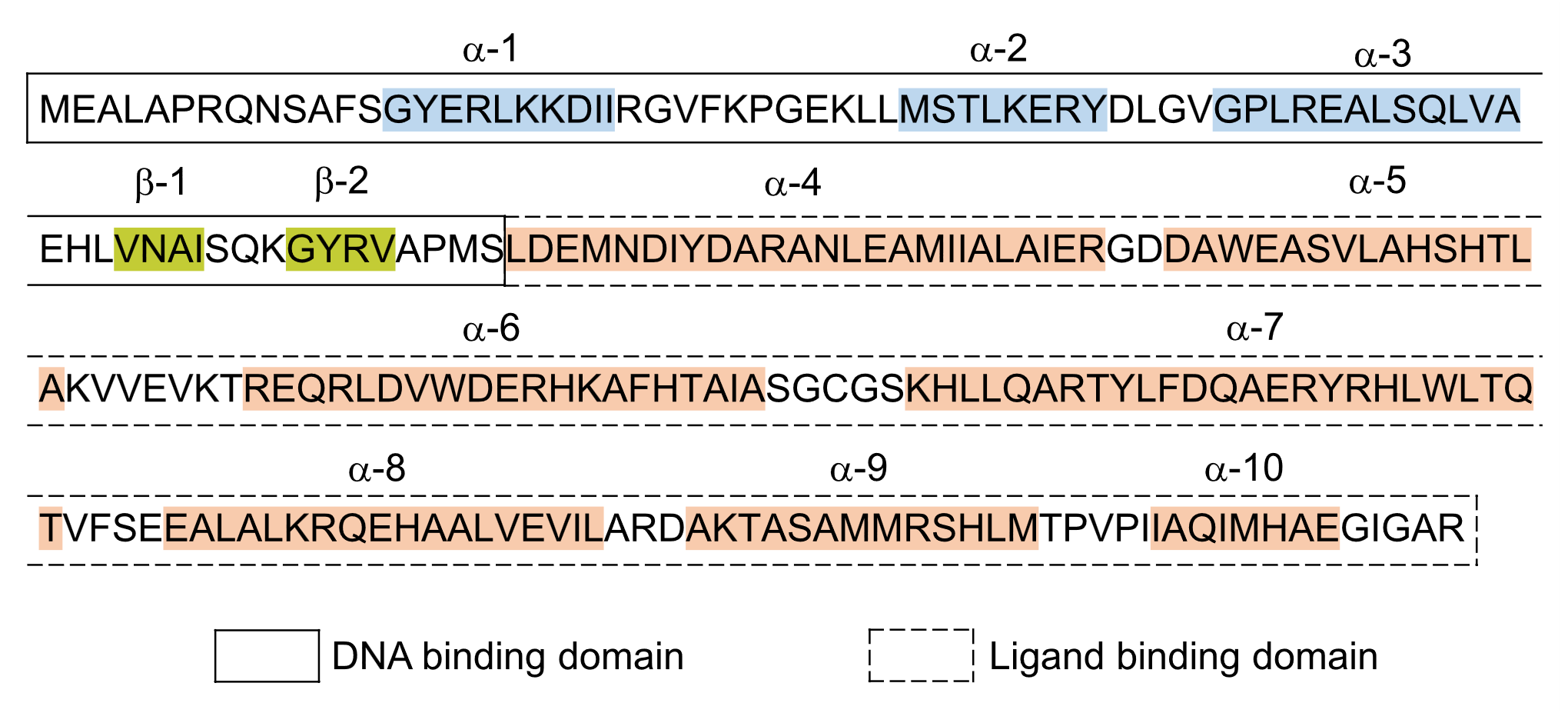


**Figure S4 Predicted secondary structure of CsiR.** CsiR-DBD and CsiR-LBD were indicated with solid and dashed boxes, respectively. The α-helices and β-sheets of CsiR were indicated with highlights and the corresponding designations were listed above the sequence.


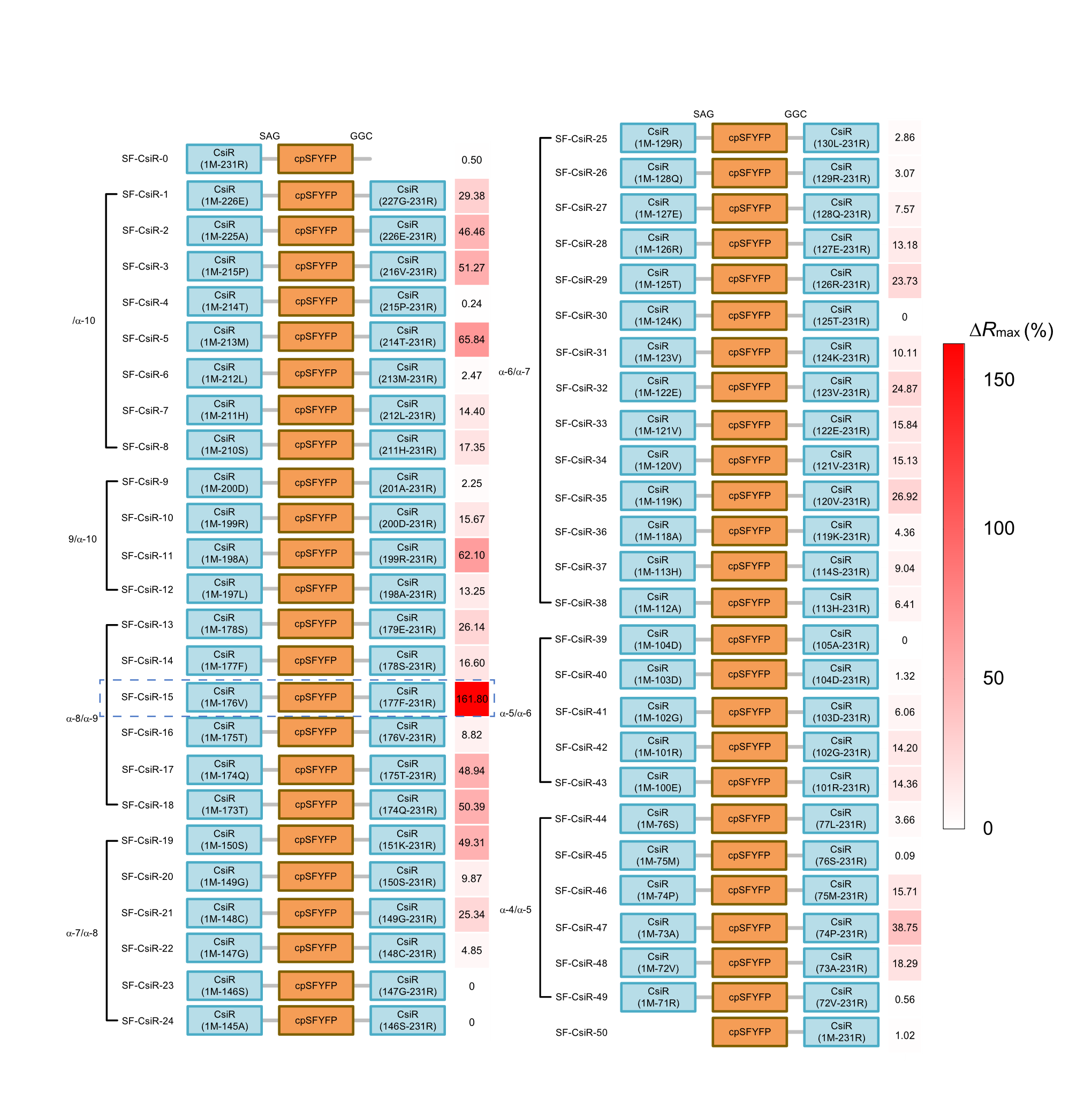


**Figure S5** **Schematic representation of the construction of the glutarate biosensor based on insertion sites screening.** The Δ*R*_max_ of different biosensor variants to glutarate were presented in a heatmap, where the colour depth represented the magnitude of Δ*R*_max_. Taking SF-CsiR-15 (Glusor-2, corresponding to 176V/177F) as an example, α-8/α-9 referred to the insertion site of cpSFYFP located between the predicted 8th and 9th α-helix of CsiR, and 176V/177F referred to the insertion site of cpSFYFP located between the residues 176 (Val) and 177 (Phe) of CsiR.


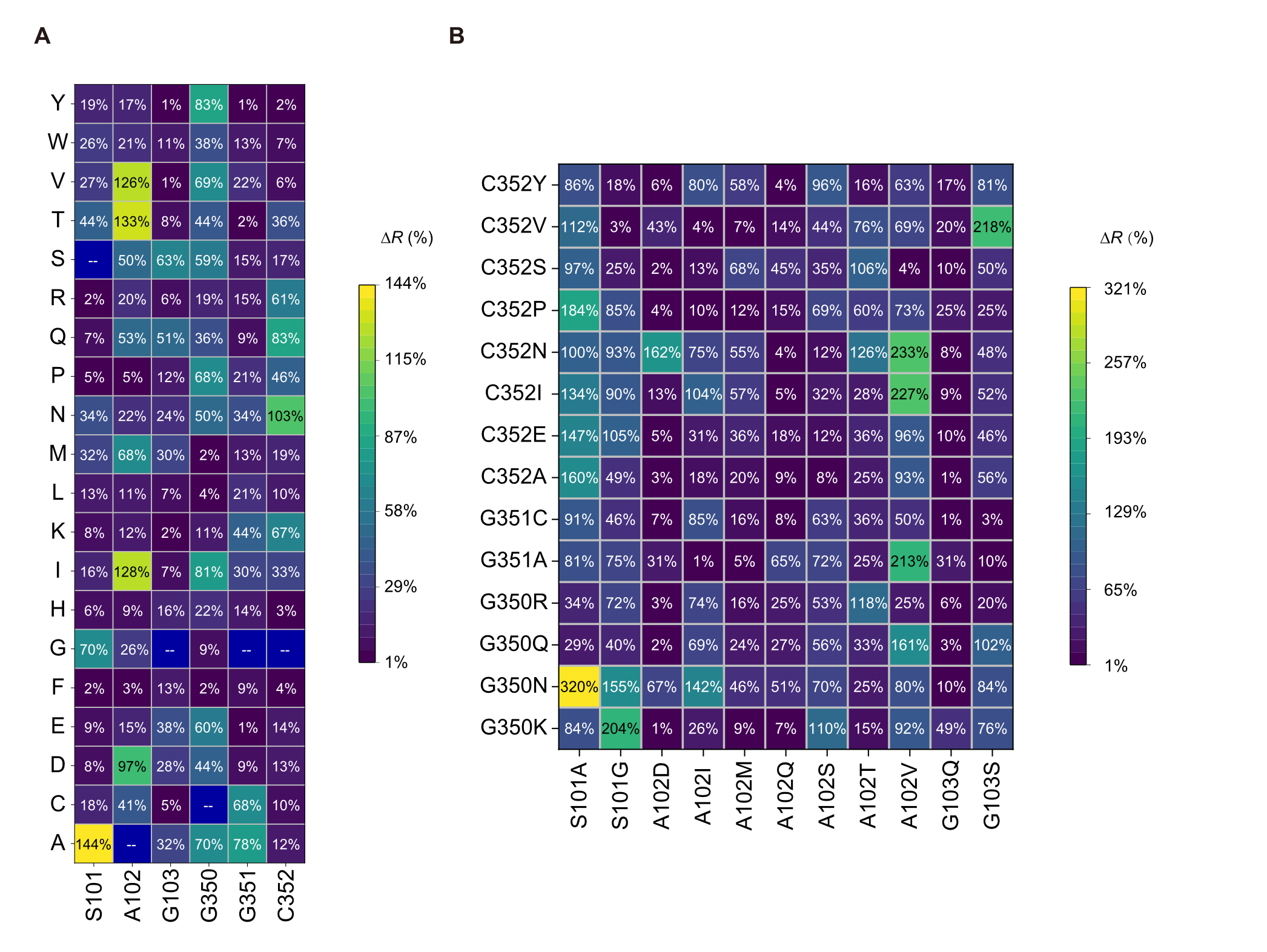


**Figure S6 Optimization of the linkers in Glusor-3.** **(A)** Site-saturation mutagenesis of the linkers in Glusor-3. The upstream linker (S101, A102, G103) and downstream linker (G350, G351, C352) were substituted with 20 different amino acids (listed in the left label). The Δ*R*_max_ of different biosensor variants to glutarate were presented in a heatmap, where the colour depth represented the magnitude of Δ*R*_max_. **(B)** Site-crossover mutagenesis of the linkers in Glusor-3. Amino acid substitution variants with Δ*R* values exceeding 50% in the site-saturation mutagenesis were selected to perform crossover mutagenesis assays. The left and bottom labels represent mutants in the upstream and downstream linkers of cpSFYFP, respectively. The Δ*R*_max_ of different biosensor variants to glutarate were presented in a heatmap, where the colour depth represented the magnitude of Δ*R*_max_.


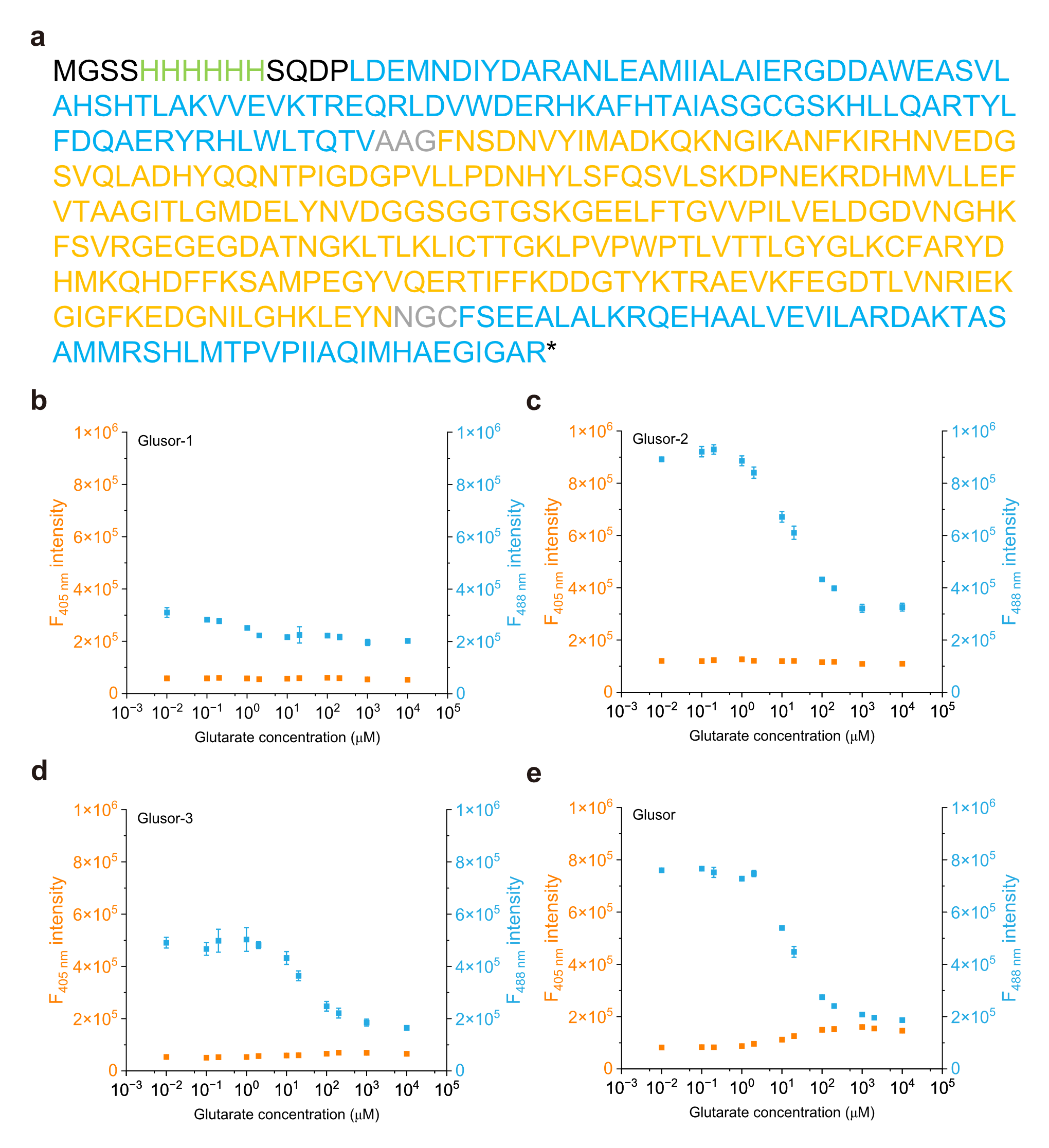


**Figure S7 Full protein sequence of Glusor.** The sequences of His_6_-tag, CsiR-LBD, linker, and cpSFYFP were indicated in green, blue, grey, and yellow, respectively.


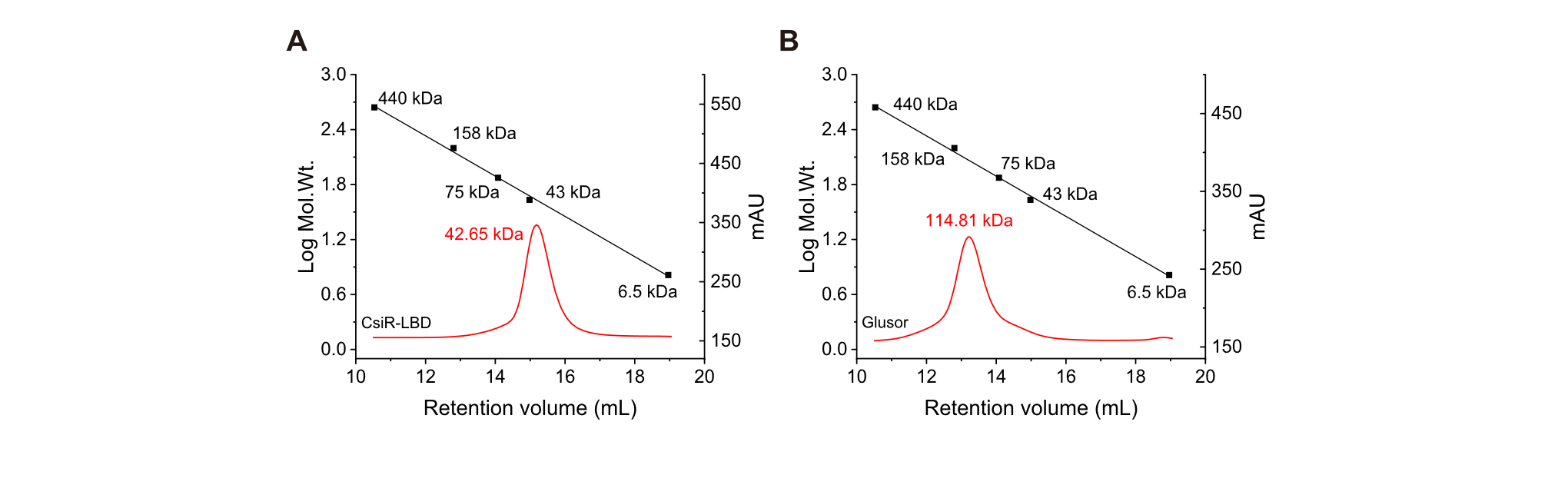


**Figure S8** **Analysis of oligomeric states of purified CsiR-LBD and Glusor. (A, B)** Gel filtration chromatography of purified CsiR-LBD **(A)**, and Glusor **(B)**. The theoretical molecular weight of the CsiR-LBD and Glusor monomer is 19.1 kDa and 46.8 kDa, respectively.


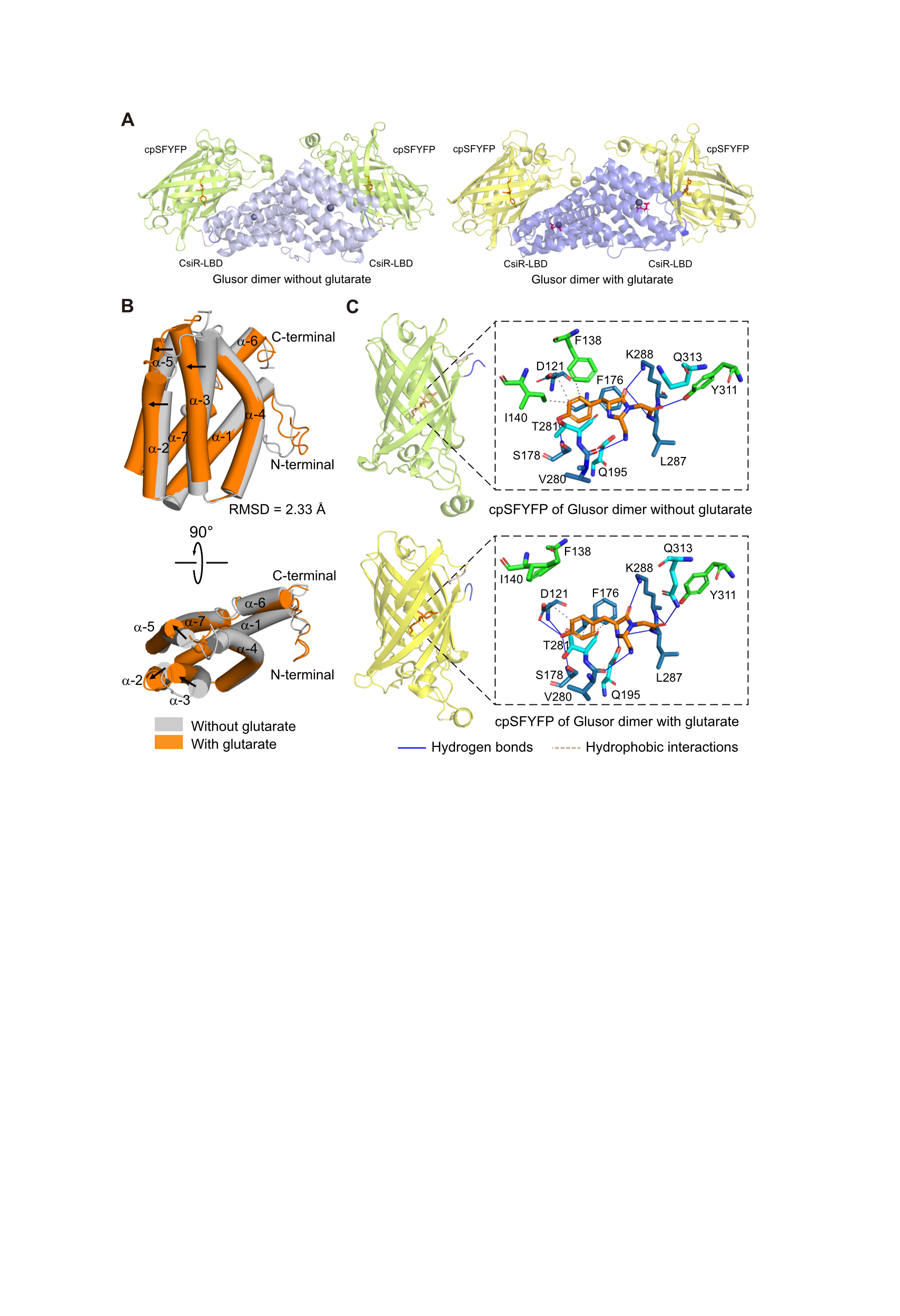


**Figure S9** **Molecular dynamics simulations of Glusor.** **(A)** Structure prediction of dimeric glutarate-free or glutarate-bound Glusor by molecular dynamics simulation. N-linkers and C-linkers of cpSFYFP are shown in blue and pink, respectively. Glutarate and fluorophore are represented as magenta and orange sticks, respectively. **(B)** Superposition of the CsiR-LBD domain of dimeric Glusor in glutarate-free and glutarate-bound states. All helices are depicted as cylinders and clearly labeled. Regions with significant differences are marked with black arrows. **(C)** Residues interacting with fluorophore in cpSFYFP domain of dimeric Glusor in glutarate-free or glutarate-bound states. Residues interacting with fluorophore only in glutarate-free state, in glutarate-bound state, or in both states are displayed in green, cyan, and blue, respectively. Fluorophores are shown in orange sticks.


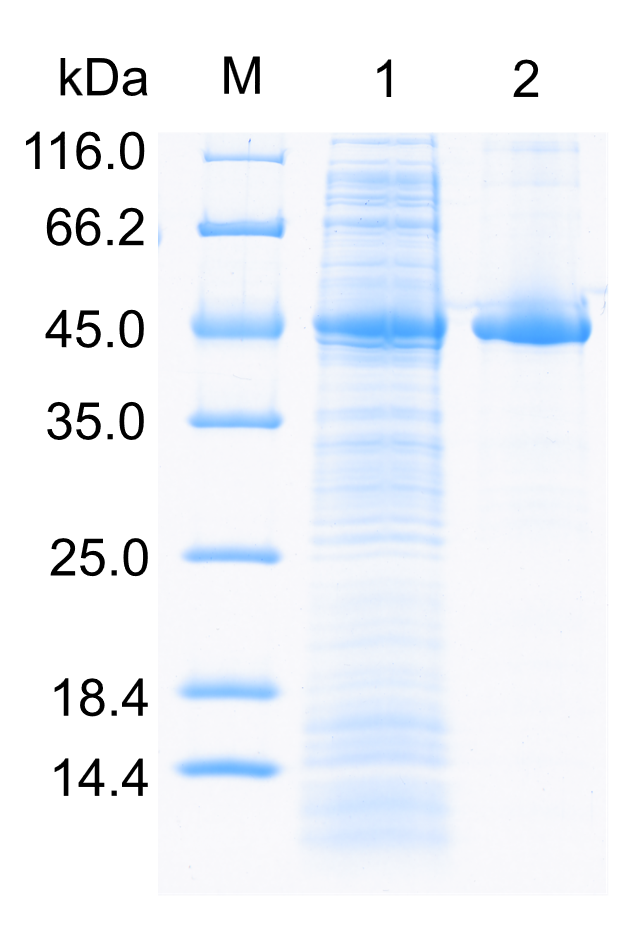


**Figure S10 SDS-PAGE analysis of the purification of Glusor.** Lane M, molecular weight markers; lane 1, crude extract of *E. coli* BL21(DE3) harboring pETDuet-Glusor; lane 2, purified His_6_-tagged Glusor using a HisTrap column.


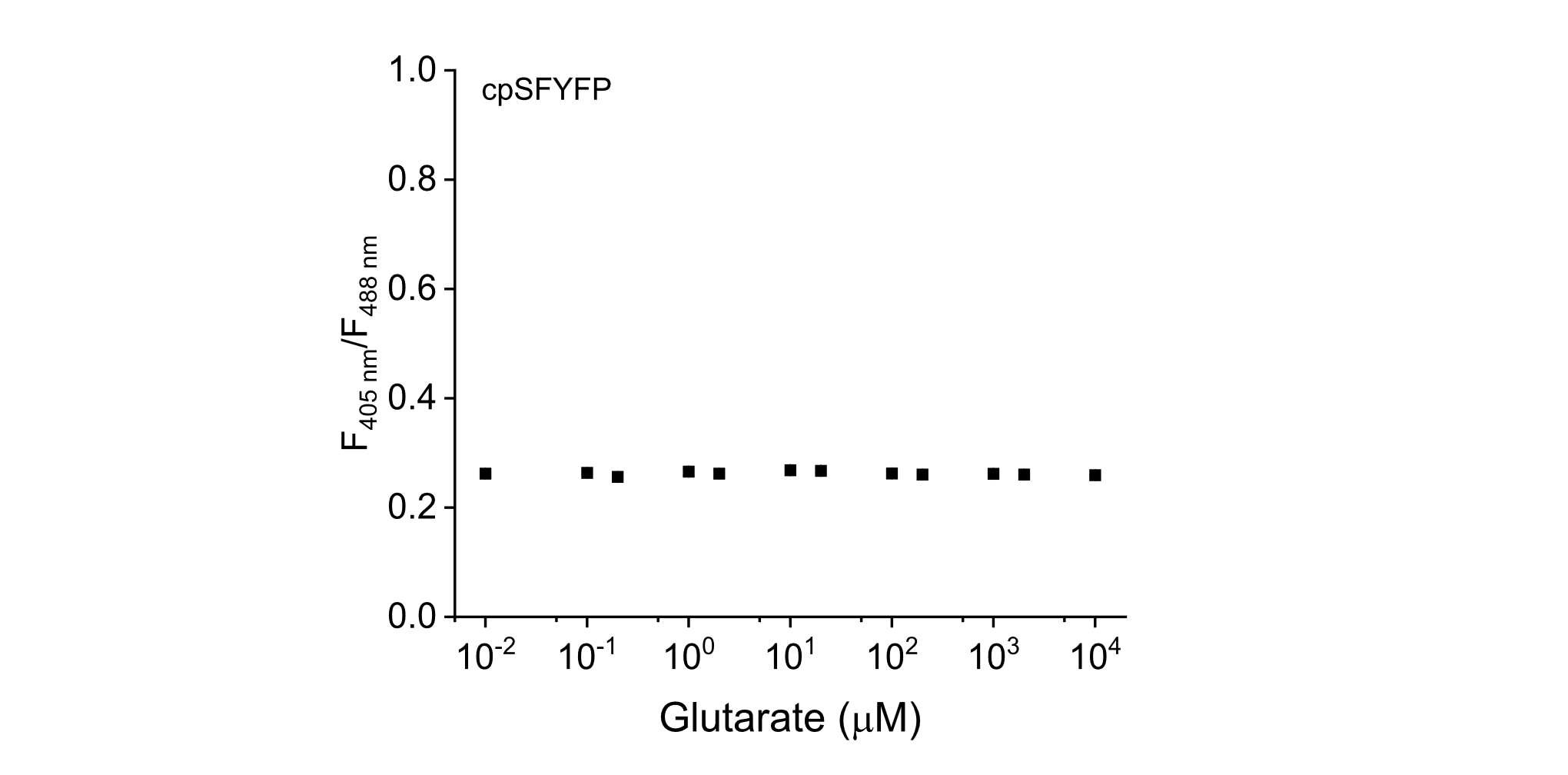


**Figure S11 Response of cpSFYFP for increasing concentrations of glutarate (10 nM to 10 mM).** All data shown are means ± s.d. (n = 3 independent experiments).


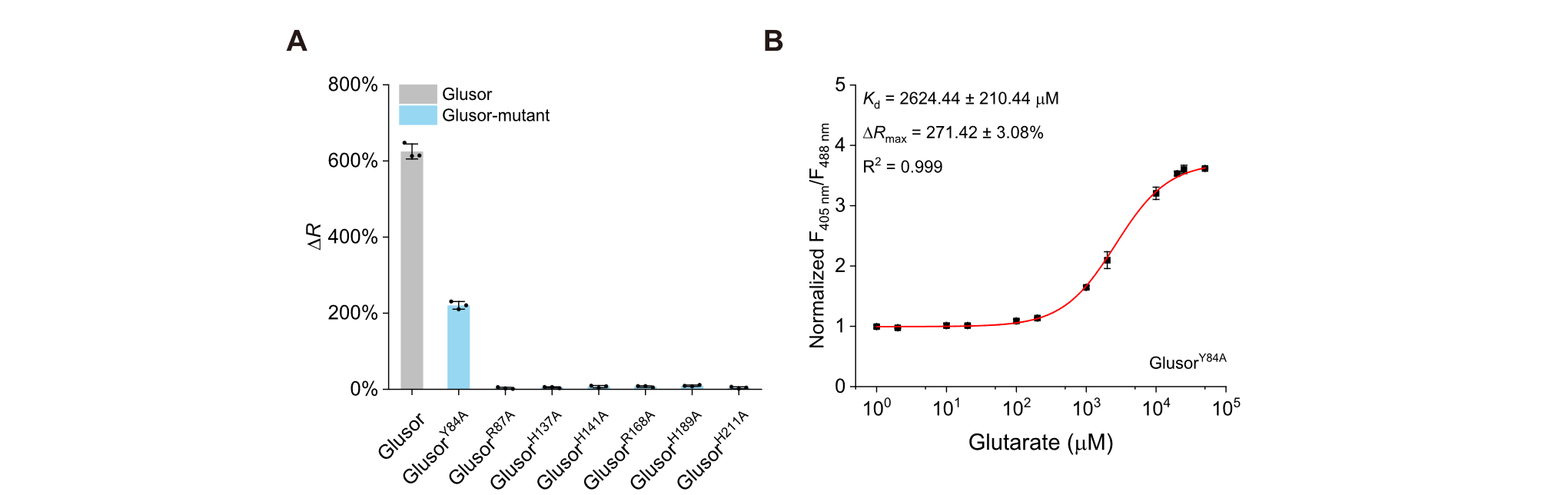


**Figure S12** **Effects of mutations in the glutarate binding sites of Glusor on response to glutarate. (A)** Comparison of the response magnitudes of Glusor and its mutants to 10 mM glutarate. **(B)** Normalized dose-response curve of Glusor^Y84A^ for glutarate (1 μM to 50 mM). Data were normalized to the initial ratio. All data shown are means ± s.d. (n = 3 independent experiments).


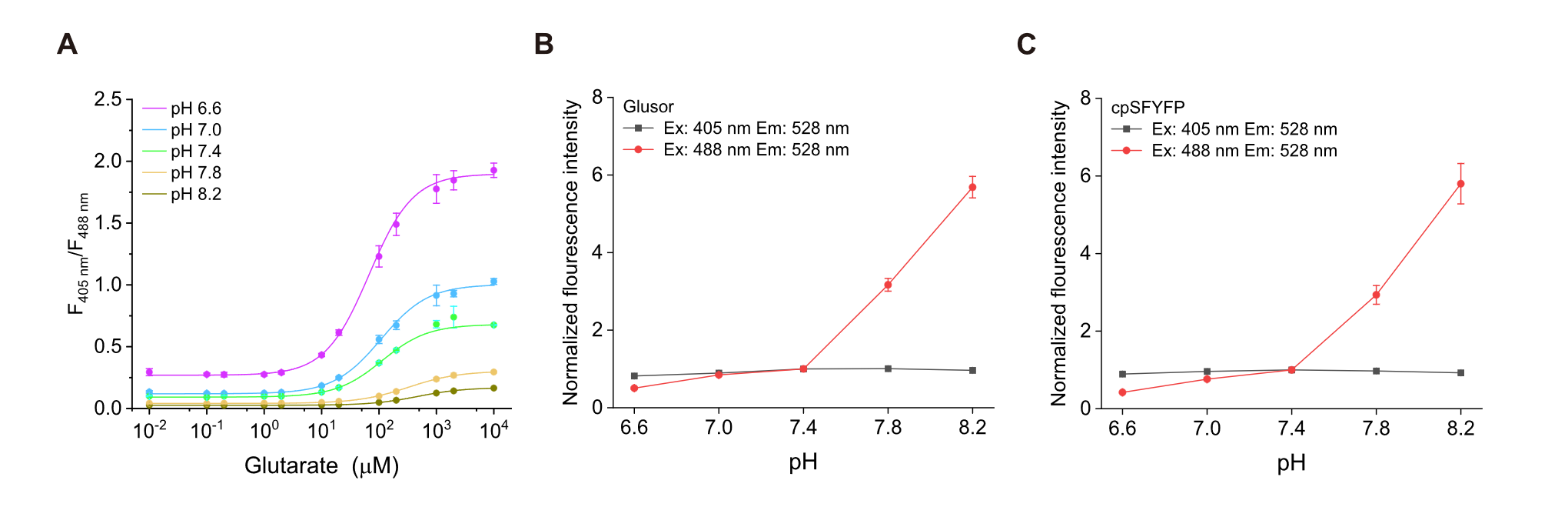


**Figure S13 pH-stability analysis of Glusor. (A)** Dose-response curves of Glusor for increasing concentrations (10 nM to 10 mM) of glutarate at the indicated pH values. **(B and C)** Fluorescence intensities of Glusor **(B)** and cpSFYFP **(C)** at the indicated pH values. Data were measured in the presence of 1 mM glutarate and normalized to the fluorescence intensity at pH 7.4. All data shown are means ± s.d. (n = 3 independent experiments).


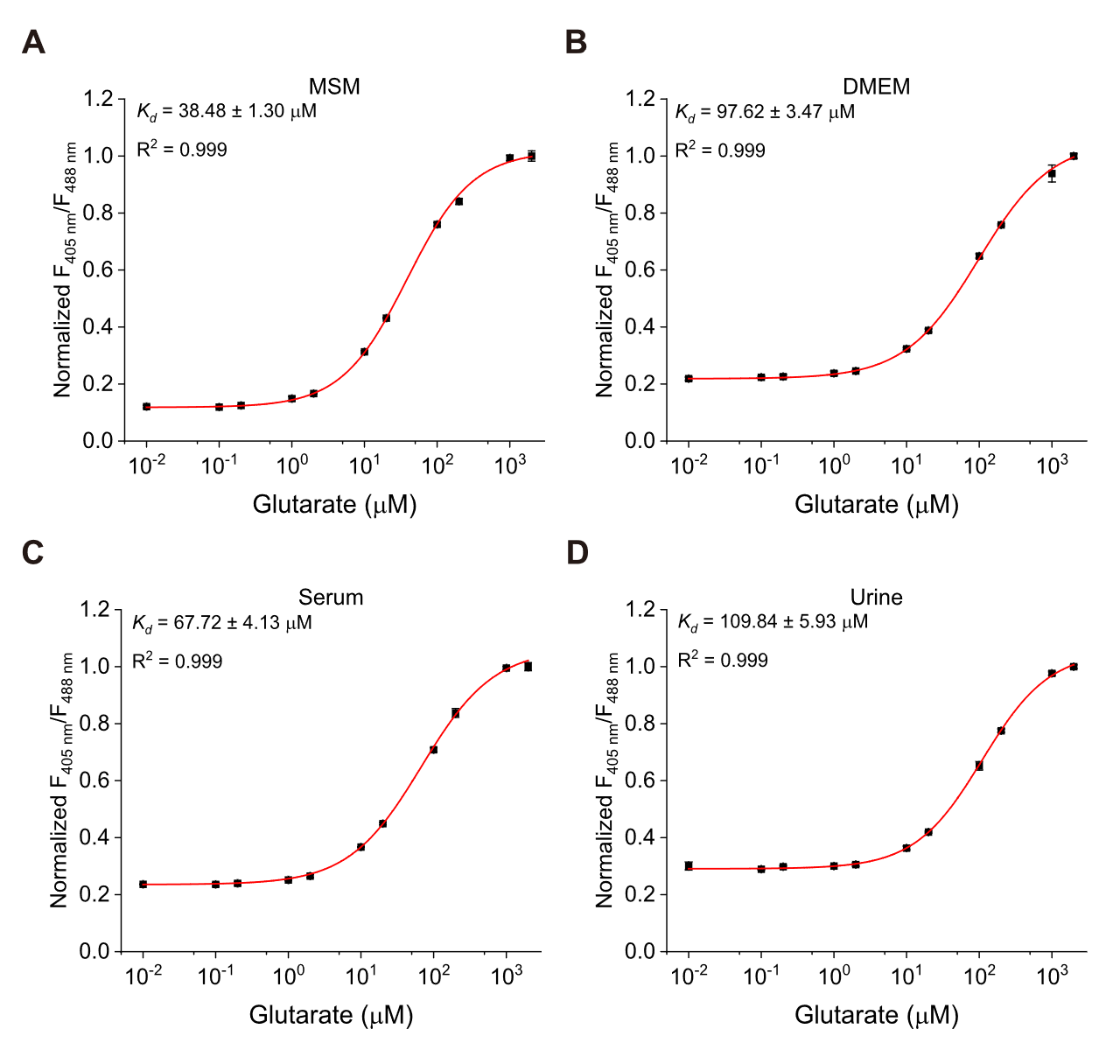


**Figure S14** **Dose-response curves of Glusor for glutarate detection in different biological samples.** **(A-D)** Dose-response curves were made by glutarate (10 nM to 2 mM) diluted in MSM **(A)**, DMEM **(B)**, serum **(C)**, and urine **(D)**. Data were normalized to the final ratio. All data shown are means ± s.d. (n = 3 independent experiments).


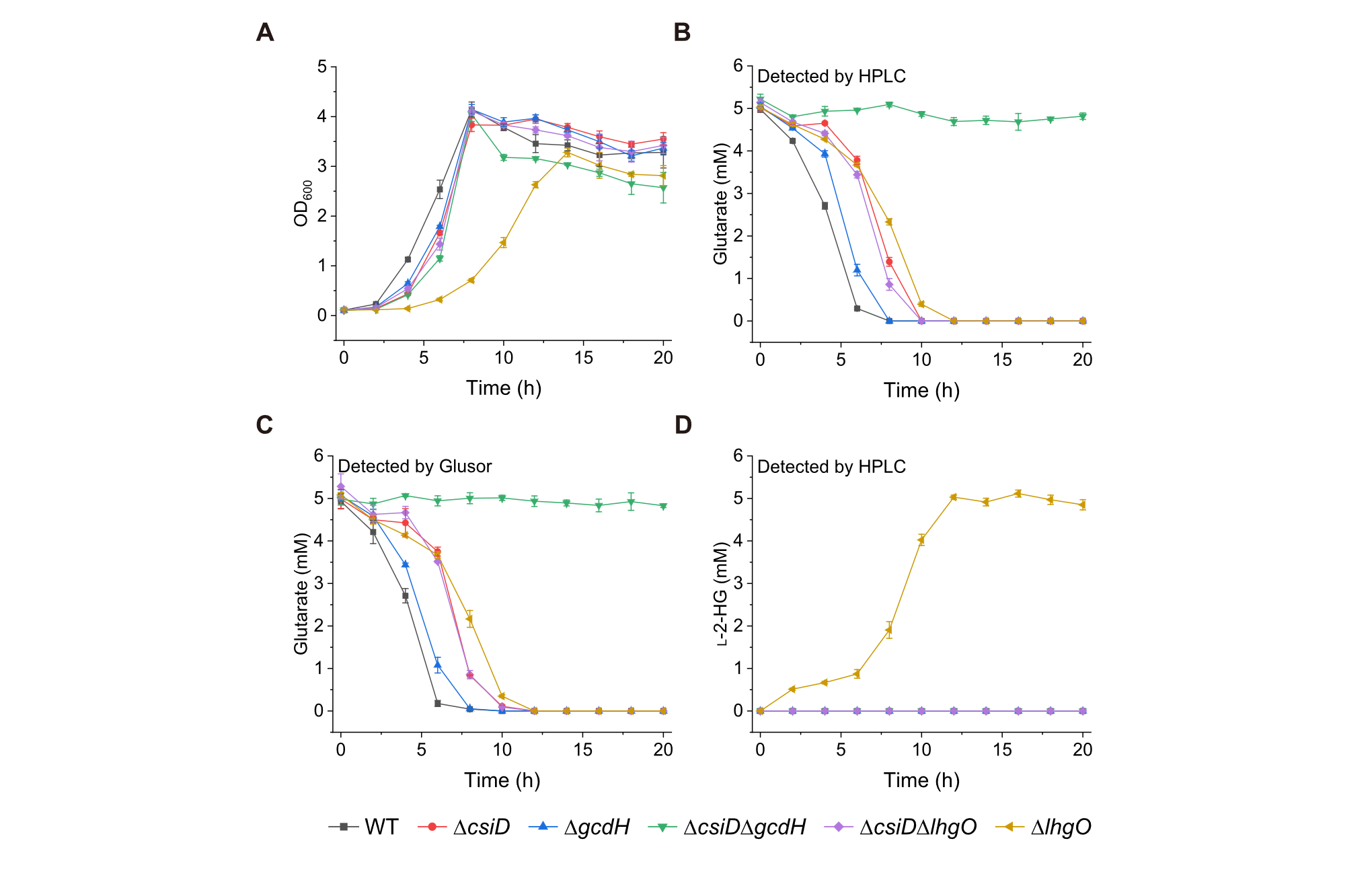


**Figure S15** **Quantification of glutarate in cultures of *P. putida* KT2440 and its derivatives using Glusor. (A)** Growth of *P. putida* KT2440 and its derivatives in MSM medium containing 20 mM glucose and 5 mM glutarate. **(B and C)** Determination of glutarate consumption of *P. putida* KT2440 and its derivatives by HPLC **(B)** and Glusor **(C)**. **(D)** Determination of extracellular l-2-HG accumulation in *P. putida* KT2440 and its derivatives by HPLC. All data shown are means ± s.d. (n = 3 independent experiments).


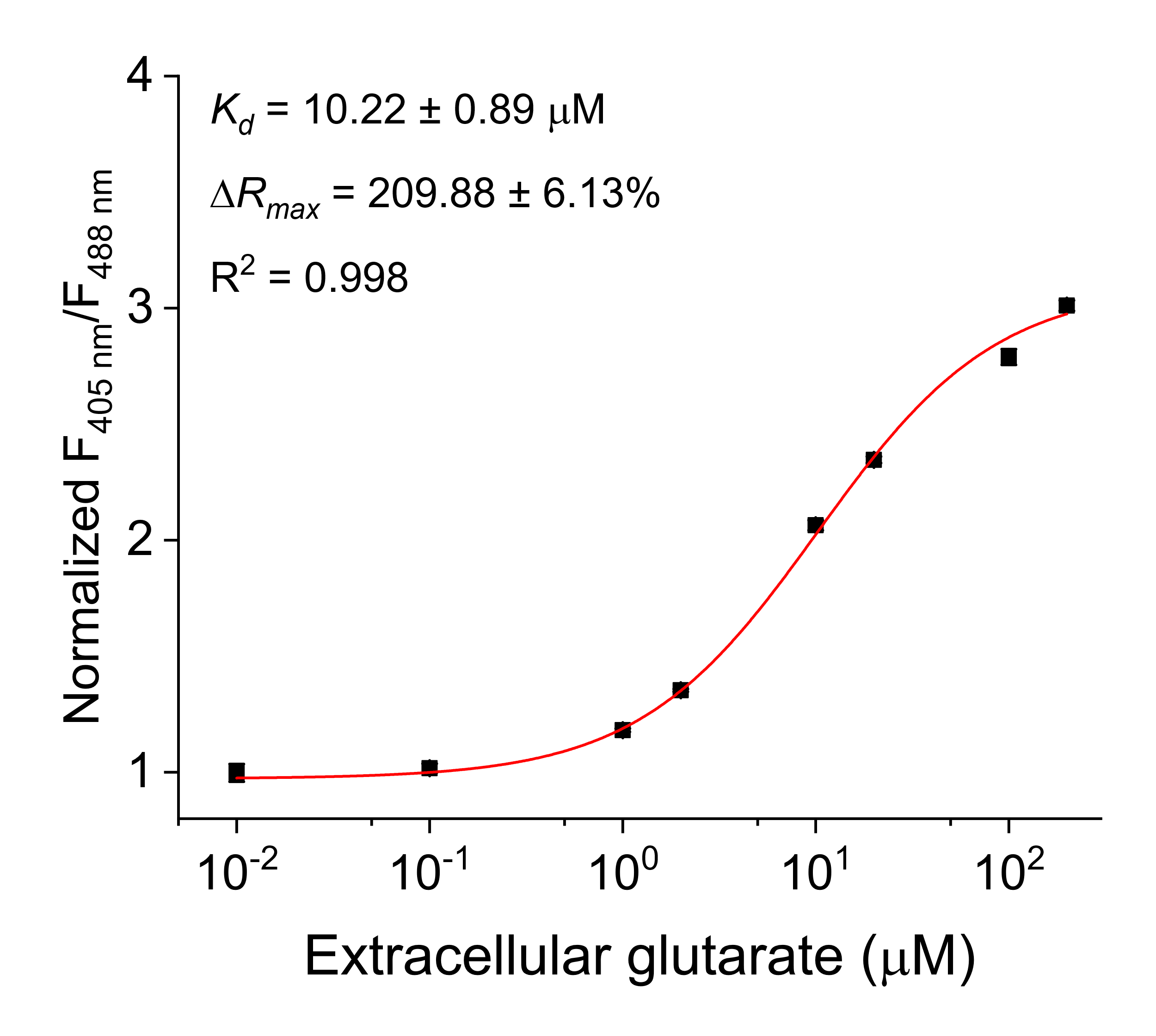


**Figure S16** **Dose-response curve of Glusor expressed in *E. coli* BL21(DE3) for increasing concentrations (10 nM to 2 mM) of exogenous glutarate.** Data were normalized to the initial ratio. All data shown are means ± s.d. (n = 3 independent experiments).


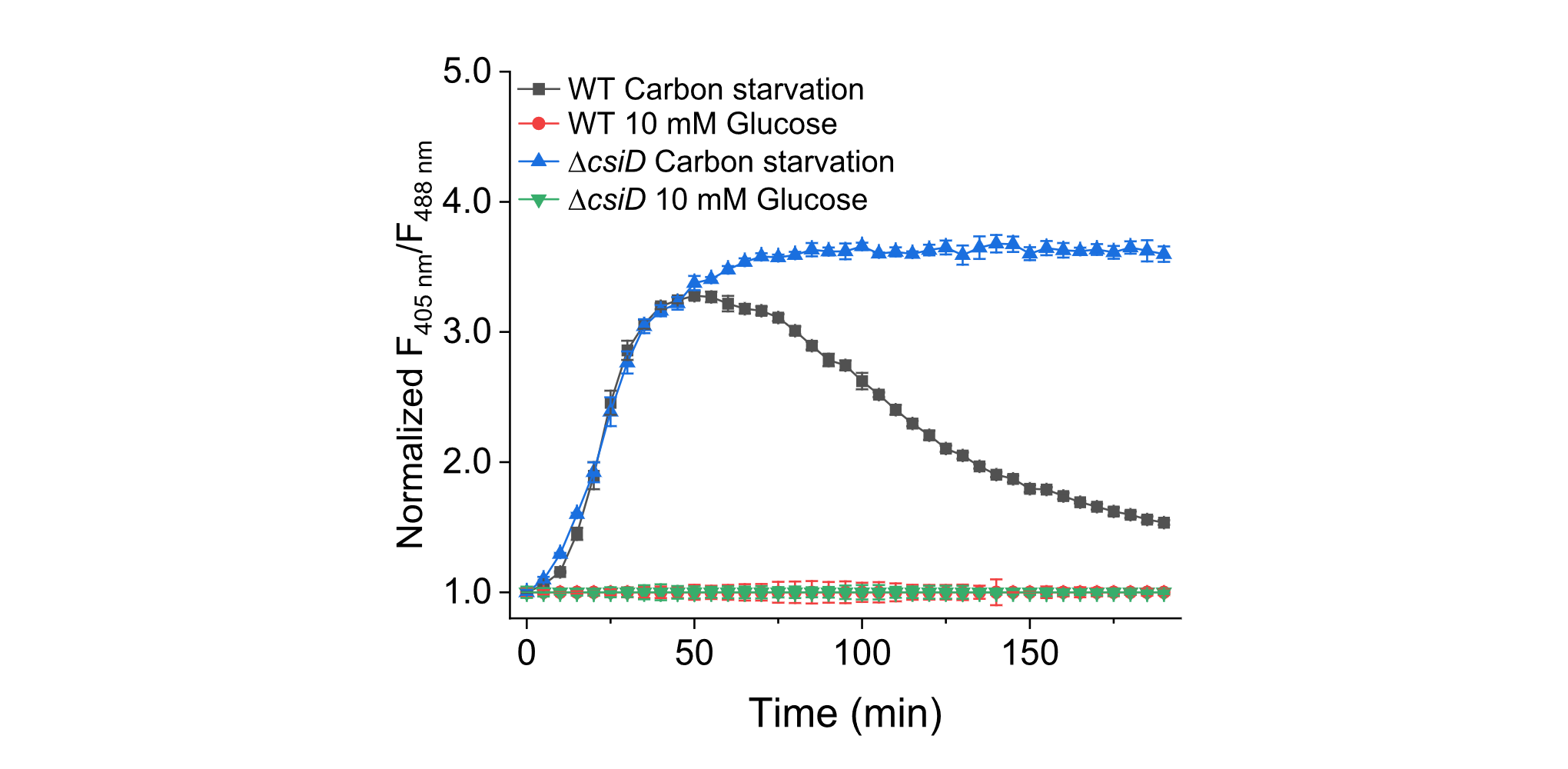


**Figure S17** **Determination of carbon starvation-induced glutarate fluctuations by using Glusor.** Data were corrected by cpSFYFP and normalized to ratios in MSM with 10 mM glucose. All data shown are means ± s.d. (n ≥ 3 independent experiments).


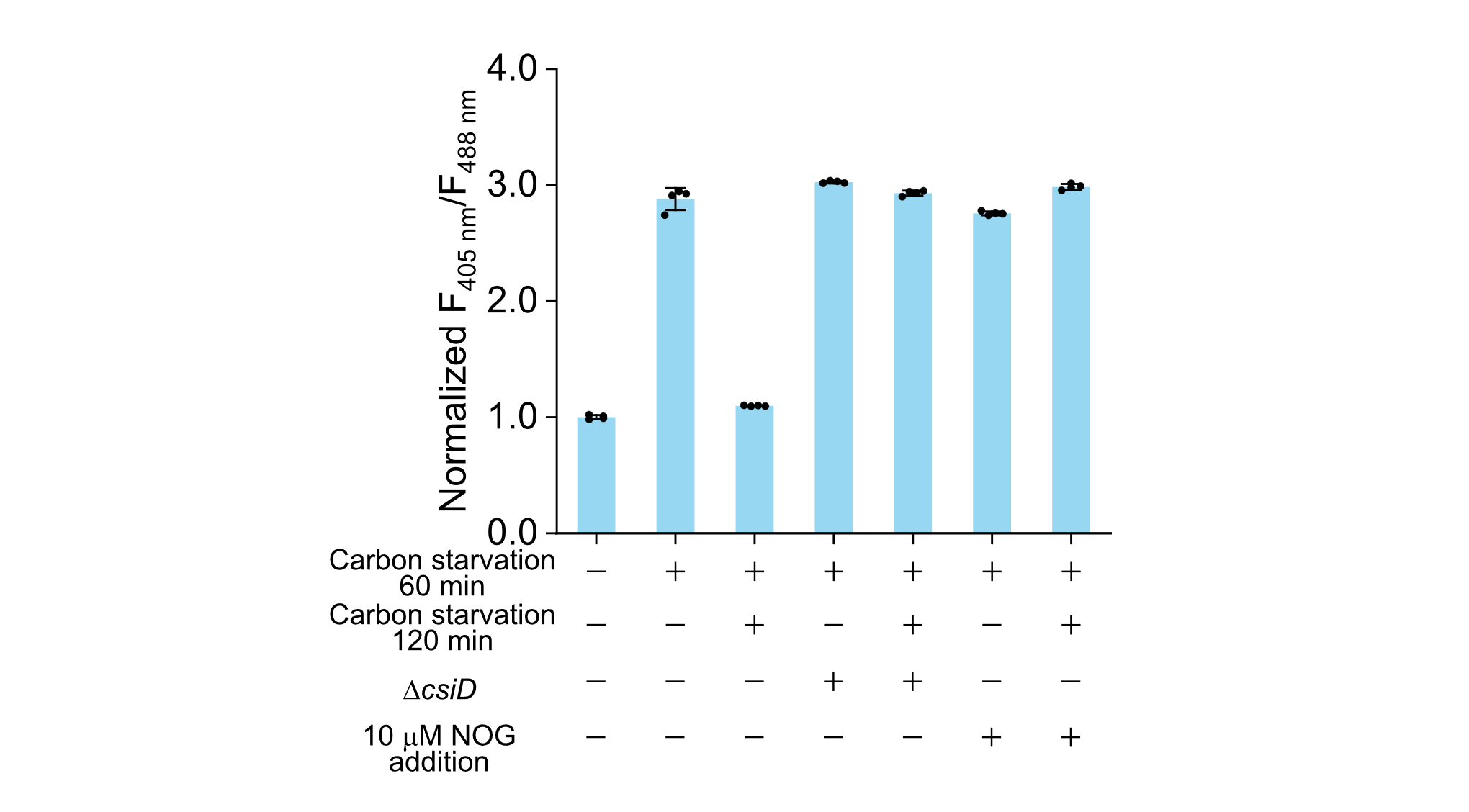


**Figure S1****8 Investigating the role of NOG in glutarate degradation mediated by CsiD in *E. coli* BL21(DE3) using Glusor.** Data were corrected by cpSFYFP and normalized to ratio of *E. coli* BL21(DE3) in MSM without NOG. All data shown are means ± s.d. (n ≥ 3 independent experiments).


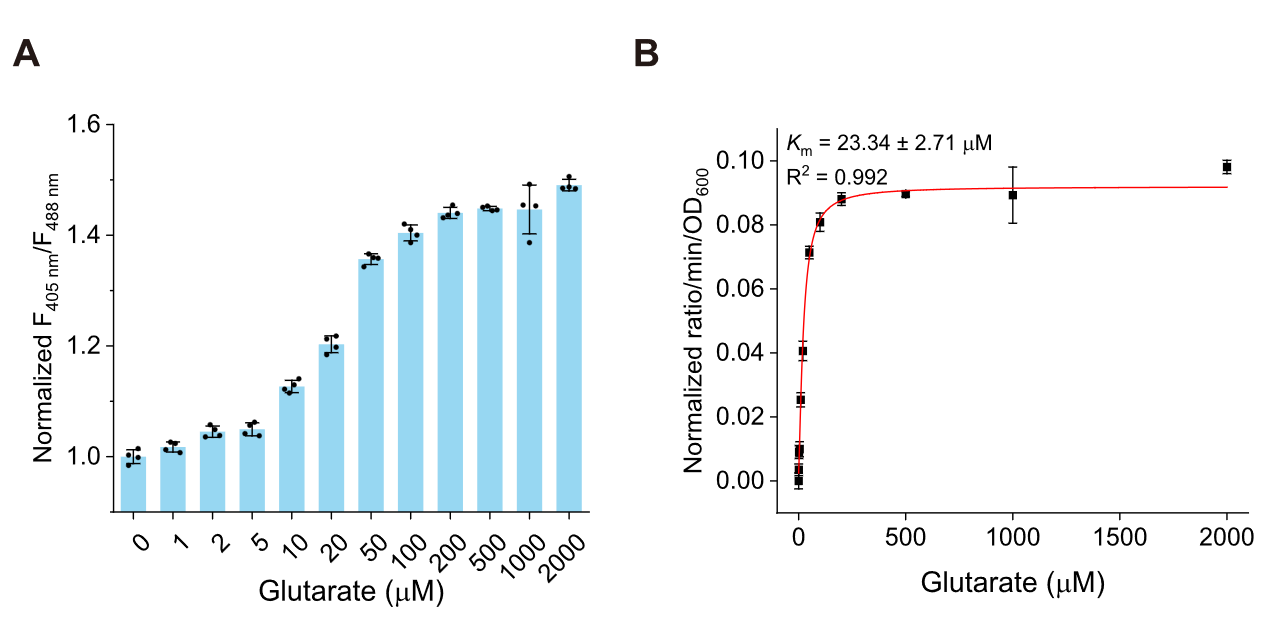


**Figure S19** **Determination of the kinetic parameters of KgtP for glutarate uptake.** Changes in fluorescence ratio of Glusor expressed in *E. coli* BL21(DE3) 1 min after addition of different concentrations of glutarate. Data were corrected by cpSFYFP and normalized to data in the absence of glutarate. All data shown are means ± s.d. (n ≥ 3 independent experiments).


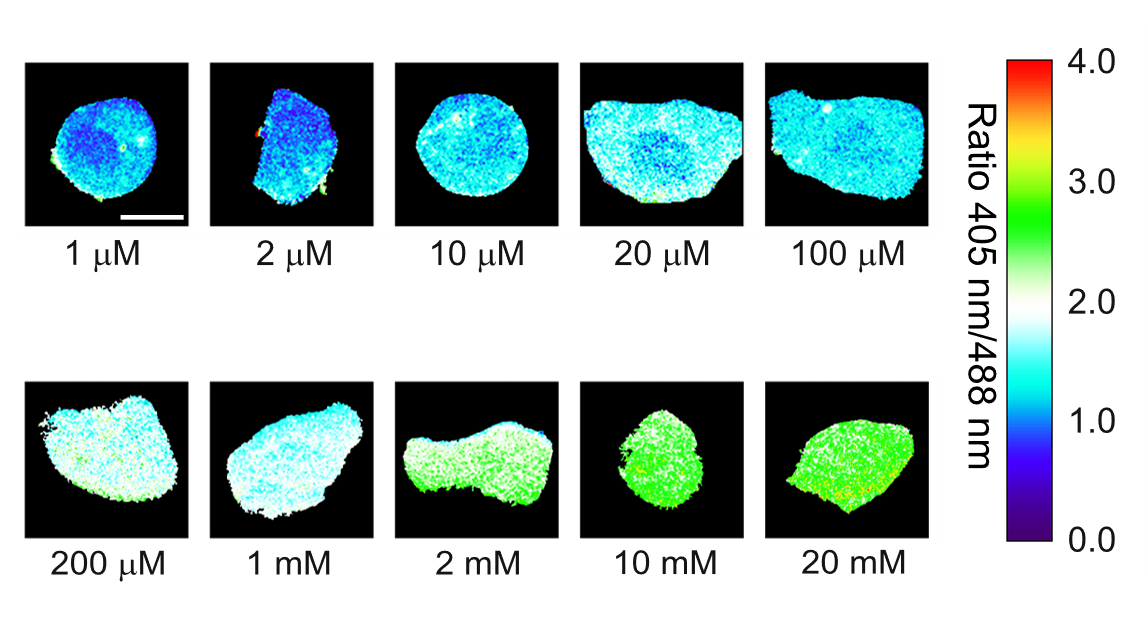


**Figure S20** **Fluorescence ratio images of Glusor expressed in HEK293FT cells in response to different concentrations of glutarate.** Cells were permeabilized with 80 μM digitonin before glutarate addition. Pseudo-colour images show pixel-by-pixel ratio of F_405 nm_/F_488 nm_. Scale bar, 10 μm.


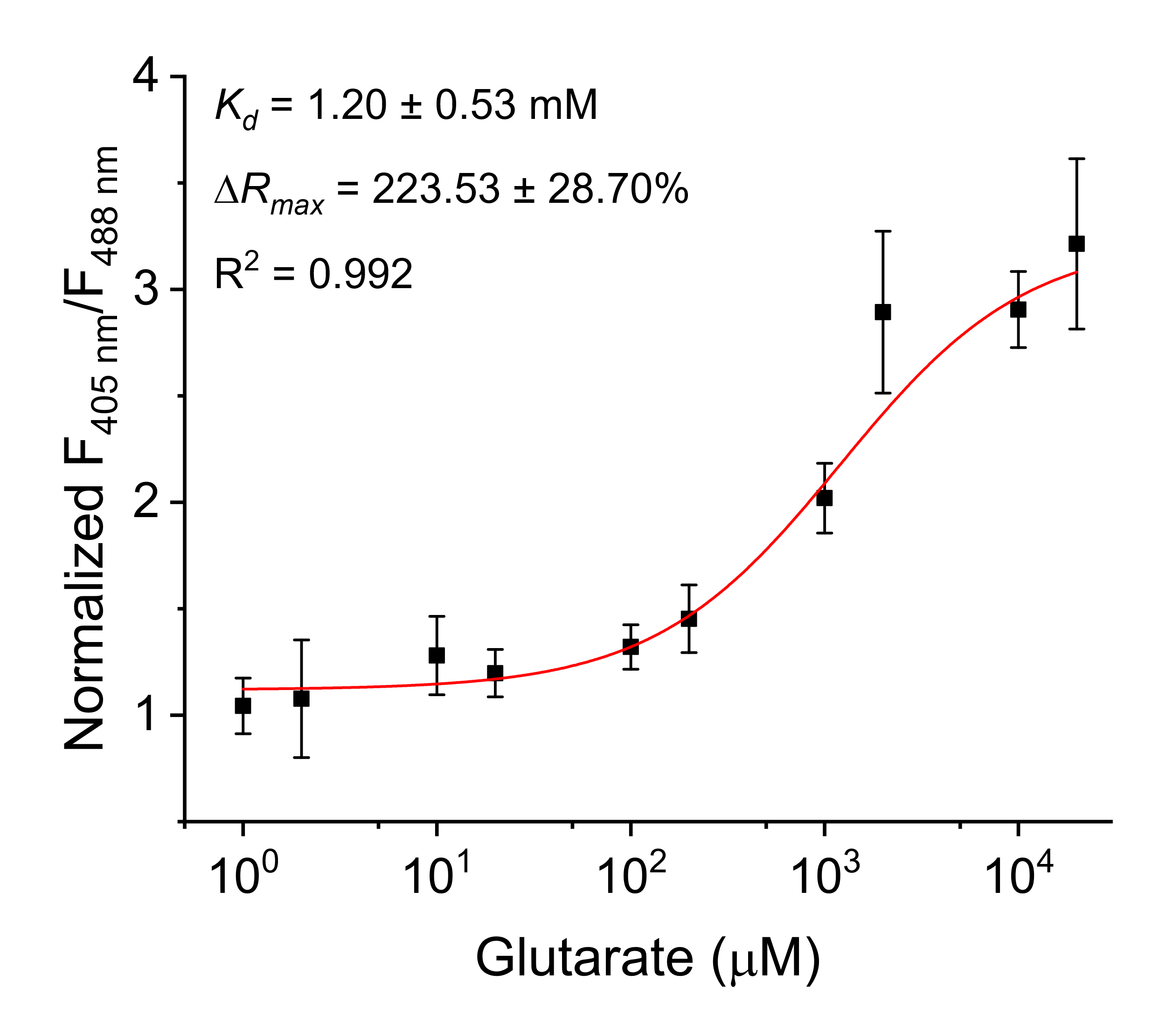


**Figure S21** **Normalized dose-response curve of Glusor expressed in HEK293FT cells for increasing concentrations (1 μM to 20 mM) of glutarate.** All data shown are means ± s.d. (n ≥ 3 independent experiments).


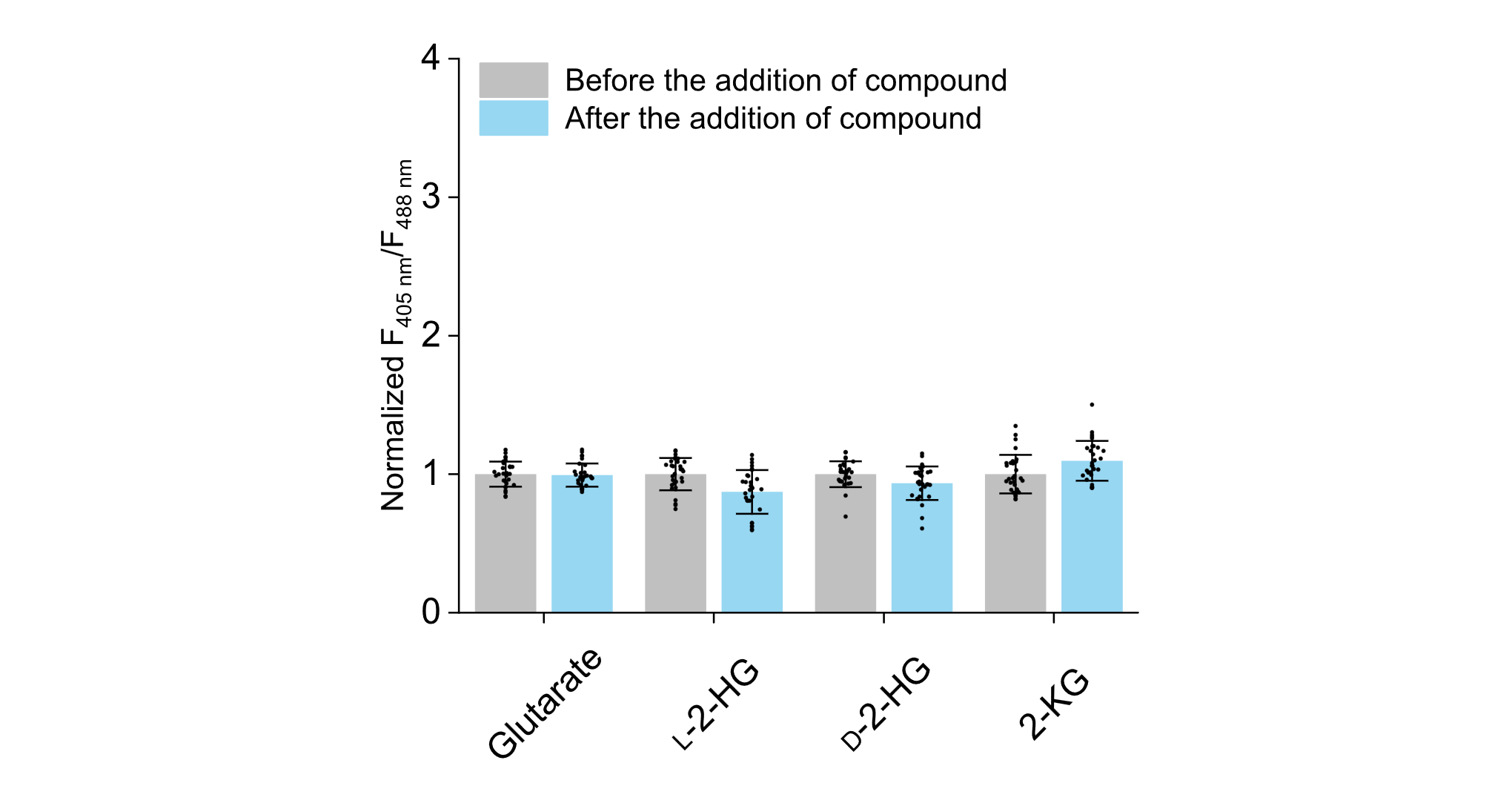


**Figure S22** **Response of cpSFYFP expressed in HEK293FT cells to glutarate, l-2-HG, d-2-HG, and 2-KG.** The fluorescence ratios of cpSFYFP before (grey) and after (blue) the addition of indicated compounds (1 mM) were recorded. Data were normalized to the ratio before the addition of any compounds (n = 30 cells).


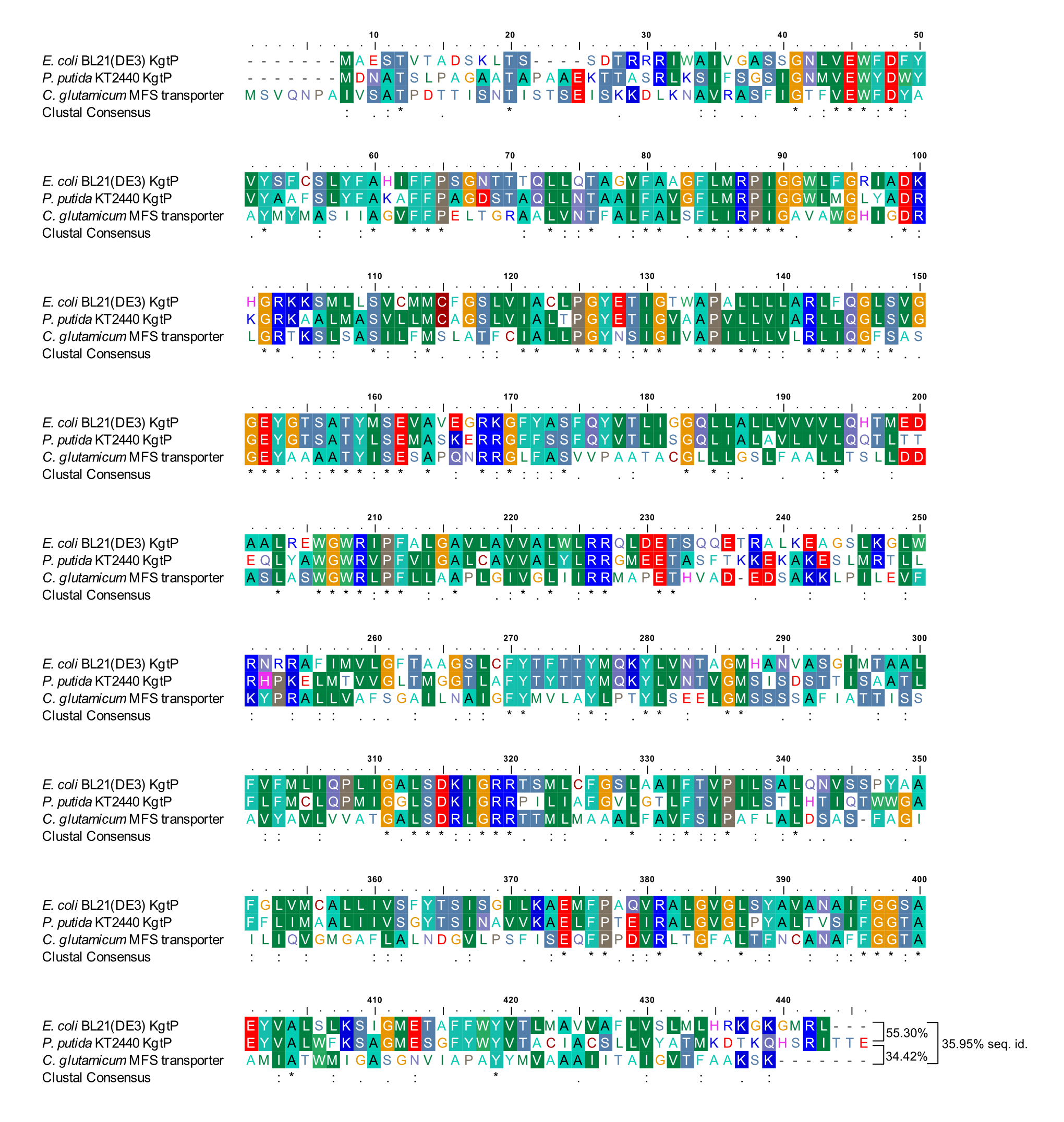


**Figure S23 Sequence alignment of KgtP homologs.** BioEdit was used to generate a multiple sequence alignment of homologs of KgtP in different glutarate producing strains. Sequence conservation is indicated as follows: (*) fully conserved, (:) strongly conserved, (.) weakly conserve.

# Supplementary Tables

#### Table S1 Evaluation of the performance of Glusor for quantification of glutarate in various biological samples.

| Condition | Concentration (μM) | | | | Accuracy (%)^a^ | | | | Precision (RSD%)^b^ |
| --- | --- | --- | --- | --- | --- | --- | --- | --- | --- |
| Standard | Sample 1 | Sample 2 | Sample 3 | Sample 4 | Sample 1 | Sample 2 | Sample 3 | Sample 4 |  |
|  | 10 | 20 | 100 | 200 | 10 | 20 | 100 | 200 |  |
| MSM | 10.11 | 19.75 | 94.95 | 222.34 | 101.10 | 98.75 | 94.95 | 111.17 | 6.82 |
| DMEM | 10.40 | 19.27 | 98.87 | 196.03 | 104.00 | 96.35 | 98.87 | 98.02 | 3.32 |
| Serum | 10.13 | 19.33 | 100.52 | 205.18 | 101.30 | 96.65 | 100.52 | 102.59 | 2.54 |
| Urine | 9.46 | 21.51 | 104.84 | 197.86 | 94.60 | 107.55 | 104.84 | 98.93 | 5.74 |

^a^Accuracy% = $\frac{\text{Concentration determined by different methods}}{\text{Defined concentration}}$

^b^Precision% = $\frac{\text{Standard derivation of accuracy}}{\text{Mean value of accuracy}}$

#### Table S2 Comparison of different glutarate detection methods.

| Method | Pretreatment | Equipment cost | Cost per test | LOD | Detection time | High-throughput |
| --- | --- | --- | --- | --- | --- | --- |
| HPLC | Deproteinization | $50,000 | $5 | 50 μM | 40 min | − |
| LC-MS/MS | Deproteinization | $150,000 | $30 | 1 μM | 40 min | − |
| Glusor | − | $50,000 | $0.01 | 1.62 μM | 10 min | Support |

#### Table S3 Strains and plasmids used in this study.

| **Strain or plasmid^a^** | **Relevant characteristics** |
| --- | --- |
| **Strain** | |
| *Escherichia coli* DH5α | F^–^ φ80*lac*Z∆M15 ∆(*lacZYA-argF*)U169 *deoR* *recA*1 *endA*1 *hsdR*17(r_K_^–^, m_K_^+^) *phoA* *supE*44 λ^–^ *thi-*1 *gyrA*96 *relA*1 |
| *E. coli* BL21(DE3) | F^–^ *ompT* *hsdSB*(*rB- mB-*) *gal*(λ *c I* 857 *ind1* *Sam*7 *nin*5 *lac*UV5-T7gene1) *dcm* (DE3) |
| *E. coli* BL21(DE3)-pETDuet-CsiR | *E. coli* BL21(DE3) carrying plasmid pETDuet-CsiR |
| *E. coli* BL21(DE3)-pETDuet-CsiR-LBD | *E. coli* BL21(DE3) carrying plasmid pETDuet-CsiR-LBD |
| *E. coli* BL21(DE3)-pETDuet-CsiR-LBD^Y84A^ | *E. coli* BL21(DE3) carrying plasmid pETDuet-CsiR-LBD^Y84A^ |
| *E. coli* BL21(DE3)-pETDuet-CsiR-LBD^R87A^ | *E. coli* BL21(DE3) carrying plasmid pETDuet-CsiR-LBD^R87A^ |
| *E. coli* BL21(DE3)-pETDuet-CsiR-LBD^H137A^ | *E. coli* BL21(DE3) carrying plasmid pETDuet-CsiR-LBD^H137A^ |
| *E. coli* BL21(DE3)-pETDuet-CsiR-LBD^H141A^ | *E. coli* BL21(DE3) carrying plasmid pETDuet-CsiR-LBD^H141A^ |
| *E. coli* BL21(DE3)-pETDuet-CsiR-LBD^R168A^ | *E. coli* BL21(DE3) carrying plasmid pETDuet-CsiR-LBD^R168A^ |
| *E. coli* BL21(DE3)-pETDuet-CsiR-LBD^H189A^ | *E. coli* BL21(DE3) carrying plasmid pETDuet-CsiR-LBD^H189A^ |
| *E. coli* BL21(DE3)-pETDuet-CsiR-LBD^H211A^ | *E. coli* BL21(DE3) carrying plasmid pETDuet-CsiR-LBD^H211A^ |
| *E. coli* BL21(DE3)-SF-CsiR-0 | *E. coli* BL21(DE3) carrying plasmid pETDuet-SF-CsiR_231R/_ |
| *E. coli* BL21(DE3)-SF-CsiR-1 | *E. coli* BL21(DE3) carrying plasmid pETDuet-SF-CsiR_226E/227G_ |
| *E. coli* BL21(DE3)-SF-CsiR-2 (Glusor-1) | *E. coli* BL21(DE3) carrying plasmid pETDuet-SF-CsiR_225A/226E_ (Glusor-1) |
| *E. coli* BL21(DE3)-SF-CsiR-3 | *E. coli* BL21(DE3) carrying plasmid pETDuet-SF-CsiR_215P/215V_ |
| *E. coli* BL21(DE3)-SF-CsiR-4 | *E. coli* BL21(DE3) carrying plasmid pETDuet-SF-CsiR_214T/215P_ |
| *E. coli* BL21(DE3)-SF-CsiR-5 | *E. coli* BL21(DE3) carrying plasmid pETDuet-SF-CsiR_213M/214T_ |
| *E. coli* BL21(DE3)-SF-CsiR-6 | *E. coli* BL21(DE3) carrying plasmid pETDuet-SF-CsiR_212L/213M_ |
| *E. coli* BL21(DE3)-SF-CsiR-7 | *E. coli* BL21(DE3) carrying plasmid pETDuet-SF-CsiR_211H/212L_ |
| *E. coli* BL21(DE3)-SF-CsiR-8 | *E. coli* BL21(DE3) carrying plasmid pETDuet-SF-CsiR_210S/211H_ |
| *E. coli* BL21(DE3)-SF-CsiR-9 | *E. coli* BL21(DE3) carrying plasmid pETDuet-SF-CsiR_200D/201A_ |
| *E. coli* BL21(DE3)-SF-CsiR-10 | *E. coli* BL21(DE3) carrying plasmid pETDuet-SF-CsiR_199R/200D_ |
| *E. coli* BL21(DE3)-SF-CsiR-11 | *E. coli* BL21(DE3) carrying plasmid pETDuet-SF-CsiR_198A/199R_ |
| *E. coli* BL21(DE3)-SF-CsiR-12 | *E. coli* BL21(DE3) carrying plasmid pETDuet-SF-CsiR_197L/198A_ |
| *E. coli* BL21(DE3)-SF-CsiR-13 | *E. coli* BL21(DE3) carrying plasmid pETDuet-SF-CsiR_178S/179E_ |
| *E. coli* BL21(DE3)-SF-CsiR-14 | *E. coli* BL21(DE3) carrying plasmid pETDuet-SF-CsiR_177F/178S_ |
| *E. coli* BL21(DE3)-SF-CsiR-15 (Glusor-2) | *E. coli* BL21(DE3) carrying plasmid pETDuet-SF-CsiR_176V/177F_ (Glusor-2) |
| *E. coli* BL21(DE3)-SF-CsiR-16 | *E. coli* BL21(DE3) carrying plasmid pETDuet-SF-CsiR_175T/176V_ |
| *E. coli* BL21(DE3)-SF-CsiR-17 | *E. coli* BL21(DE3) carrying plasmid pETDuet-SF-CsiR_174Q/175T_ |
| *E. coli* BL21(DE3)-SF-CsiR-18 | *E. coli* BL21(DE3) carrying plasmid pETDuet-SF-CsiR_173T/174Q_ |
| *E. coli* BL21(DE3)-SF-CsiR-19 | *E. coli* BL21(DE3) carrying plasmid pETDuet-SF-CsiR_150S/151K_ |
| *E. coli* BL21(DE3)-SF-CsiR-20 | *E. coli* BL21(DE3) carrying plasmid pETDuet-SF-CsiR_149G/150S_ |
| *E. coli* BL21(DE3)-SF-CsiR-21 | *E. coli* BL21(DE3) carrying plasmid pETDuet-SF-CsiR_148C/149G_ |
| *E. coli* BL21(DE3)-SF-CsiR-22 | *E. coli* BL21(DE3) carrying plasmid pETDuet-SF-CsiR_147G/148C_ |
| *E. coli* BL21(DE3)-SF-CsiR-23 | *E. coli* BL21(DE3) carrying plasmid pETDuet-SF-CsiR_146S/147G_ |
| *E. coli* BL21(DE3)-SF-CsiR-24 | *E. coli* BL21(DE3) carrying plasmid pETDuet-SF-CsiR_145A/146S_ |
| *E. coli* BL21(DE3)-SF-CsiR-25 | *E. coli* BL21(DE3) carrying plasmid pETDuet-SF-CsiR_129R/130L_ |
| *E. coli* BL21(DE3)-SF-CsiR-26 | *E. coli* BL21(DE3) carrying plasmid pETDuet-SF-CsiR_128Q/129R_ |
| *E. coli* BL21(DE3)-SF-CsiR-27 | *E. coli* BL21(DE3) carrying plasmid pETDuet-SF-CsiR_127E/128Q_ |
| *E. coli* BL21(DE3)-SF-CsiR-28 | *E. coli* BL21(DE3) carrying plasmid pETDuet-SF-CsiR_126R/127E_ |
| *E. coli* BL21(DE3)-SF-CsiR-29 | *E. coli* BL21(DE3) carrying plasmid pETDuet-SF-CsiR_125T/126R_ |
| *E. coli* BL21(DE3)-SF-CsiR-30 | *E. coli* BL21(DE3) carrying plasmid pETDuet-SF-CsiR_124K/125T_ |
| *E. coli* BL21(DE3)-SF-CsiR-31 | *E. coli* BL21(DE3) carrying plasmid pETDuet-SF-CsiR_123V/124K_ |
| *E. coli* BL21(DE3)-SF-CsiR-32 | *E. coli* BL21(DE3) carrying plasmid pETDuet-SF-CsiR_122E/123V_ |
| *E. coli* BL21(DE3)-SF-CsiR-33 | *E. coli* BL21(DE3) carrying plasmid pETDuet-SF-CsiR_121V/122E_ |
| *E. coli* BL21(DE3)-SF-CsiR-34 | *E. coli* BL21(DE3) carrying plasmid pETDuet-SF-CsiR_120V/121V_ |
| *E. coli* BL21(DE3)-SF-CsiR-35 | *E. coli* BL21(DE3) carrying plasmid pETDuet-SF-CsiR_119K/120V_ |
| *E. coli* BL21(DE3)-SF-CsiR-36 | *E. coli* BL21(DE3) carrying plasmid pETDuet-SF-CsiR_118A/119K_ |
| *E. coli* BL21(DE3)-SF-CsiR-37 | *E. coli* BL21(DE3) carrying plasmid pETDuet-SF-CsiR_113H/114S_ |
| *E. coli* BL21(DE3)-SF-CsiR-38 | *E. coli* BL21(DE3) carrying plasmid pETDuet-SF-CsiR_112A/113H_ |
| *E. coli* BL21(DE3)-SF-CsiR-39 | *E. coli* BL21(DE3) carrying plasmid pETDuet-SF-CsiR_104D/105A_ |
| *E. coli* BL21(DE3)-SF-CsiR-40 | *E. coli* BL21(DE3) carrying plasmid pETDuet-SF-CsiR_103D/104D_ |
| *E. coli* BL21(DE3)-SF-CsiR-41 | *E. coli* BL21(DE3) carrying plasmid pETDuet-SF-CsiR_102G/103D_ |
| *E. coli* BL21(DE3)-SF-CsiR-42 | *E. coli* BL21(DE3) carrying plasmid pETDuet-SF-CsiR_101R/102G_ |
| *E. coli* BL21(DE3)-SF-CsiR-43 | *E. coli* BL21(DE3) carrying plasmid pETDuet-SF-CsiR_100E/101R_ |
| *E. coli* BL21(DE3)-SF-CsiR-44 | *E. coli* BL21(DE3) carrying plasmid pETDuet-SF-CsiR_76S/77L_ |
| *E. coli* BL21(DE3)-SF-CsiR-45 | *E. coli* BL21(DE3) carrying plasmid pETDuet-SF-CsiR_75M/76S_ |
| *E. coli* BL21(DE3)-SF-CsiR-46 | *E. coli* BL21(DE3) carrying plasmid pETDuet-SF-CsiR_74P/75M_ |
| *E. coli* BL21(DE3)-SF-CsiR-47 | *E. coli* BL21(DE3) carrying plasmid pETDuet-SF-CsiR_73A/74P_ |
| *E. coli* BL21(DE3)-SF-CsiR-48 | *E. coli* BL21(DE3) carrying plasmid pETDuet-SF-CsiR_72V/73A_ |
| *E. coli* BL21(DE3)-SF-CsiR-49 | *E. coli* BL21(DE3) carrying plasmid pETDuet-SF-CsiR_71R/72V_ |
| *E. coli* BL21(DE3)-SF-CsiR-50 | *E. coli* BL21(DE3) carrying plasmid pETDuet-SF-CsiR_/1M_ |
| *E. coli* BL21(DE3)-SF-CsiR-15-nodbd (Glusor-3) | *E. coli* BL21(DE3) carrying plasmid pETDuet-SF-CsiR-15-nodbd (Glusor-3) |
| *E. coli* BL21(DE3)-Glusor-3-S101A | *E. coli* BL21(DE3) carrying plasmid pETDuet-muta-S101A |
| *E. coli* BL21(DE3)-Glusor-3-S101C | *E. coli* BL21(DE3) carrying plasmid pETDuet-muta-S101C |
| *E. coli* BL21(DE3)-Glusor-3-S101D | *E. coli* BL21(DE3) carrying plasmid pETDuet-muta-S101D |
| *E. coli* BL21(DE3)-Glusor-3-S101E | *E. coli* BL21(DE3) carrying plasmid pETDuet-muta-S101E |
| *E. coli* BL21(DE3)-Glusor-3-S101F | *E. coli* BL21(DE3) carrying plasmid pETDuet-muta-S101F |
| *E. coli* BL21(DE3)-Glusor-3-S101G | *E. coli* BL21(DE3) carrying plasmid pETDuet-muta-S101G |
| *E. coli* BL21(DE3)-Glusor-3-S101H | *E. coli* BL21(DE3) carrying plasmid pETDuet-muta-S101H |
| *E. coli* BL21(DE3)-Glusor-3-S101I | *E. coli* BL21(DE3) carrying plasmid pETDuet-muta-S101I |
| *E. coli* BL21(DE3)-Glusor-3-S101K | *E. coli* BL21(DE3) carrying plasmid pETDuet-muta-S101K |
| *E. coli* BL21(DE3)-Glusor-3-S101L | *E. coli* BL21(DE3) carrying plasmid pETDuet-muta-S101L |
| *E. coli* BL21(DE3)-Glusor-3-S101M | *E. coli* BL21(DE3) carrying plasmid pETDuet-muta-S101M |
| *E. coli* BL21(DE3)-Glusor-3-S101N | *E. coli* BL21(DE3) carrying plasmid pETDuet-muta-S101N |
| *E. coli* BL21(DE3)-Glusor-3-S101P | *E. coli* BL21(DE3) carrying plasmid pETDuet-muta-S101P |
| *E. coli* BL21(DE3)-Glusor-3-S101Q | *E. coli* BL21(DE3) carrying plasmid pETDuet-muta-S101Q |
| *E. coli* BL21(DE3)-Glusor-3-S101R | *E. coli* BL21(DE3) carrying plasmid pETDuet-muta-S101R |
| *E. coli* BL21(DE3)-Glusor-3-S101T | *E. coli* BL21(DE3) carrying plasmid pETDuet-muta-S101T |
| *E. coli* BL21(DE3)-Glusor-3-S101V | *E. coli* BL21(DE3) carrying plasmid pETDuet-muta-S101V |
| *E. coli* BL21(DE3)-Glusor-3-S101W | *E. coli* BL21(DE3) carrying plasmid pETDuet-muta-S101W |
| *E. coli* BL21(DE3)-Glusor-3-S101Y | *E. coli* BL21(DE3) carrying plasmid pETDuet-muta-S101Y |
| *E. coli* BL21(DE3)-Glusor-3-A102C | *E. coli* BL21(DE3) carrying plasmid pETDuet-muta-A102C |
| *E. coli* BL21(DE3)-Glusor-3-A102D | *E. coli* BL21(DE3) carrying plasmid pETDuet-muta-A102D |
| *E. coli* BL21(DE3)-Glusor-3-A102E | *E. coli* BL21(DE3) carrying plasmid pETDuet-muta-A102E |
| *E. coli* BL21(DE3)-Glusor-3-A102F | *E. coli* BL21(DE3) carrying plasmid pETDuet-muta-A102F |
| *E. coli* BL21(DE3)-Glusor-3-A102G | *E. coli* BL21(DE3) carrying plasmid pETDuet-muta-A102G |
| *E. coli* BL21(DE3)-Glusor-3-A102H | *E. coli* BL21(DE3) carrying plasmid pETDuet-muta-A102H |
| *E. coli* BL21(DE3)-Glusor-3-A102I | *E. coli* BL21(DE3) carrying plasmid pETDuet-muta-A102I |
| *E. coli* BL21(DE3)-Glusor-3-A102K | *E. coli* BL21(DE3) carrying plasmid pETDuet-muta-A102K |
| *E. coli* BL21(DE3)-Glusor-3-A102L | *E. coli* BL21(DE3) carrying plasmid pETDuet-muta-A102L |
| *E. coli* BL21(DE3)-Glusor-3-A102M | *E. coli* BL21(DE3) carrying plasmid pETDuet-muta-A102M |
| *E. coli* BL21(DE3)-Glusor-3-A102N | *E. coli* BL21(DE3) carrying plasmid pETDuet-muta-A102N |
| *E. coli* BL21(DE3)-Glusor-3-A102P | *E. coli* BL21(DE3) carrying plasmid pETDuet-muta-A102P |
| *E. coli* BL21(DE3)-Glusor-3-A102Q | *E. coli* BL21(DE3) carrying plasmid pETDuet-muta-A102Q |
| *E. coli* BL21(DE3)-Glusor-3-A102R | *E. coli* BL21(DE3) carrying plasmid pETDuet-muta-A102R |
| *E. coli* BL21(DE3)-Glusor-3-A102S | *E. coli* BL21(DE3) carrying plasmid pETDuet-muta-A102S |
| *E. coli* BL21(DE3)-Glusor-3-A102T | *E. coli* BL21(DE3) carrying plasmid pETDuet-muta-A102T |
| *E. coli* BL21(DE3)-Glusor-3-A102V | *E. coli* BL21(DE3) carrying plasmid pETDuet-muta-A102V |
| *E. coli* BL21(DE3)-Glusor-3-A102W | *E. coli* BL21(DE3) carrying plasmid pETDuet-muta-A102W |
| *E. coli* BL21(DE3)-Glusor-3-A102Y | *E. coli* BL21(DE3) carrying plasmid pETDuet-muta-A102Y |
| *E. coli* BL21(DE3)-Glusor-3-G103A | *E. coli* BL21(DE3) carrying plasmid pETDuet-muta-G103A |
| *E. coli* BL21(DE3)-Glusor-3-G103C | *E. coli* BL21(DE3) carrying plasmid pETDuet-muta-G103C |
| *E. coli* BL21(DE3)-Glusor-3-G103D | *E. coli* BL21(DE3) carrying plasmid pETDuet-muta-G103D |
| *E. coli* BL21(DE3)-Glusor-3-G103E | *E. coli* BL21(DE3) carrying plasmid pETDuet-muta-G103E |
| *E. coli* BL21(DE3)-Glusor-3-G103F | *E. coli* BL21(DE3) carrying plasmid pETDuet-muta-G103F |
| *E. coli* BL21(DE3)-Glusor-3-G103H | *E. coli* BL21(DE3) carrying plasmid pETDuet-muta-G103H |
| *E. coli* BL21(DE3)-Glusor-3-G103I | *E. coli* BL21(DE3) carrying plasmid pETDuet-muta-G103I |
| *E. coli* BL21(DE3)-Glusor-3-G103K | *E. coli* BL21(DE3) carrying plasmid pETDuet-muta-G103K |
| *E. coli* BL21(DE3)-Glusor-3-G103L | *E. coli* BL21(DE3) carrying plasmid pETDuet-muta-G103L |
| *E. coli* BL21(DE3)-Glusor-3-G103M | *E. coli* BL21(DE3) carrying plasmid pETDuet-muta-G103M |
| *E. coli* BL21(DE3)-Glusor-3-G103N | *E. coli* BL21(DE3) carrying plasmid pETDuet-muta-G103N |
| *E. coli* BL21(DE3)-Glusor-3-G103P | *E. coli* BL21(DE3) carrying plasmid pETDuet-muta-G103P |
| *E. coli* BL21(DE3)-Glusor-3-G103Q | *E. coli* BL21(DE3) carrying plasmid pETDuet-muta-G103Q |
| *E. coli* BL21(DE3)-Glusor-3-G103R | *E. coli* BL21(DE3) carrying plasmid pETDuet-muta-G103R |
| *E. coli* BL21(DE3)-Glusor-3-G103S | *E. coli* BL21(DE3) carrying plasmid pETDuet-muta-G103S |
| *E. coli* BL21(DE3)-Glusor-3-G103T | *E. coli* BL21(DE3) carrying plasmid pETDuet-muta-G103T |
| *E. coli* BL21(DE3)-Glusor-3-G103V | *E. coli* BL21(DE3) carrying plasmid pETDuet-muta-G103V |
| *E. coli* BL21(DE3)-Glusor-3-G103W | *E. coli* BL21(DE3) carrying plasmid pETDuet-muta-G103W |
| *E. coli* BL21(DE3)-Glusor-3-G103Y | *E. coli* BL21(DE3) carrying plasmid pETDuet-muta-G103Y |
| *E. coli* BL21(DE3)-Glusor-3-G350A | *E. coli* BL21(DE3) carrying plasmid pETDuet-muta-G350A |
| *E. coli* BL21(DE3)-Glusor-3-G350C | *E. coli* BL21(DE3) carrying plasmid pETDuet-muta-G350C |
| *E. coli* BL21(DE3)-Glusor-3-G350D | *E. coli* BL21(DE3) carrying plasmid pETDuet-muta-G350D |
| *E. coli* BL21(DE3)-Glusor-3-G350E | *E. coli* BL21(DE3) carrying plasmid pETDuet-muta-G350E |
| *E. coli* BL21(DE3)-Glusor-3-G350F | *E. coli* BL21(DE3) carrying plasmid pETDuet-muta-G350F |
| *E. coli* BL21(DE3)-Glusor-3-G350H | *E. coli* BL21(DE3) carrying plasmid pETDuet-muta-G350H |
| *E. coli* BL21(DE3)-Glusor-3-G350I | *E. coli* BL21(DE3) carrying plasmid pETDuet-muta-G350I |
| *E. coli* BL21(DE3)-Glusor-3-G350K | *E. coli* BL21(DE3) carrying plasmid pETDuet-muta-G350K |
| *E. coli* BL21(DE3)-Glusor-3-G350L | *E. coli* BL21(DE3) carrying plasmid pETDuet-muta-G350L |
| *E. coli* BL21(DE3)-Glusor-3-G350M | *E. coli* BL21(DE3) carrying plasmid pETDuet-muta-G350M |
| *E. coli* BL21(DE3)-Glusor-3-G350N | *E. coli* BL21(DE3) carrying plasmid pETDuet-muta-G350N |
| *E. coli* BL21(DE3)-Glusor-3-G350P | *E. coli* BL21(DE3) carrying plasmid pETDuet-muta-G350P |
| *E. coli* BL21(DE3)-Glusor-3-G350Q | *E. coli* BL21(DE3) carrying plasmid pETDuet-muta-G350Q |
| *E. coli* BL21(DE3)-Glusor-3-G350R | *E. coli* BL21(DE3) carrying plasmid pETDuet-muta-G350R |
| *E. coli* BL21(DE3)-Glusor-3-G350S | *E. coli* BL21(DE3) carrying plasmid pETDuet-muta-G350S |
| *E. coli* BL21(DE3)-Glusor-3-G350T | *E. coli* BL21(DE3) carrying plasmid pETDuet-muta-G350T |
| *E. coli* BL21(DE3)-Glusor-3-G350V | *E. coli* BL21(DE3) carrying plasmid pETDuet-muta-G350V |
| *E. coli* BL21(DE3)-Glusor-3-G350W | *E. coli* BL21(DE3) carrying plasmid pETDuet-muta-G350W |
| *E. coli* BL21(DE3)-Glusor-3-G350Y | *E. coli* BL21(DE3) carrying plasmid pETDuet-muta-G350Y |
| *E. coli* BL21(DE3)-Glusor-3-G351A | *E. coli* BL21(DE3) carrying plasmid pETDuet-muta-G351A |
| *E. coli* BL21(DE3)-Glusor-3-G351C | *E. coli* BL21(DE3) carrying plasmid pETDuet-muta-G351C |
| *E. coli* BL21(DE3)-Glusor-3-G351D | *E. coli* BL21(DE3) carrying plasmid pETDuet-muta-G351D |
| *E. coli* BL21(DE3)-Glusor-3-G351E | *E. coli* BL21(DE3) carrying plasmid pETDuet-muta-G351E |
| *E. coli* BL21(DE3)-Glusor-3-G351F | *E. coli* BL21(DE3) carrying plasmid pETDuet-muta-G351F |
| *E. coli* BL21(DE3)-Glusor-3-G351H | *E. coli* BL21(DE3) carrying plasmid pETDuet-muta-G351H |
| *E. coli* BL21(DE3)-Glusor-3-G351I | *E. coli* BL21(DE3) carrying plasmid pETDuet-muta-G351I |
| *E. coli* BL21(DE3)-Glusor-3-G351K | *E. coli* BL21(DE3) carrying plasmid pETDuet-muta-G351K |
| *E. coli* BL21(DE3)-Glusor-3-G351L | *E. coli* BL21(DE3) carrying plasmid pETDuet-muta-G351L |
| *E. coli* BL21(DE3)-Glusor-3-G351M | *E. coli* BL21(DE3) carrying plasmid pETDuet-muta-G351M |
| *E. coli* BL21(DE3)-Glusor-3-G351N | *E. coli* BL21(DE3) carrying plasmid pETDuet-muta-G351N |
| *E. coli* BL21(DE3)-Glusor-3-G351P | *E. coli* BL21(DE3) carrying plasmid pETDuet-muta-G351P |
| *E. coli* BL21(DE3)-Glusor-3-G351Q | *E. coli* BL21(DE3) carrying plasmid pETDuet-muta-G351Q |
| *E. coli* BL21(DE3)-Glusor-3-G351R | *E. coli* BL21(DE3) carrying plasmid pETDuet-muta-G351R |
| *E. coli* BL21(DE3)-Glusor-3-G351S | *E. coli* BL21(DE3) carrying plasmid pETDuet-muta-G351S |
| *E. coli* BL21(DE3)-Glusor-3-G351T | *E. coli* BL21(DE3) carrying plasmid pETDuet-muta-G351T |
| *E. coli* BL21(DE3)-Glusor-3-G351V | *E. coli* BL21(DE3) carrying plasmid pETDuet-muta-G351V |
| *E. coli* BL21(DE3)-Glusor-3-G351W | *E. coli* BL21(DE3) carrying plasmid pETDuet-muta-G351W |
| *E. coli* BL21(DE3)-Glusor-3-G351Y | *E. coli* BL21(DE3) carrying plasmid pETDuet-muta-G351Y |
| *E. coli* BL21(DE3)-Glusor-3-C352A | *E. coli* BL21(DE3) carrying plasmid pETDuet-muta-C352A |
| *E. coli* BL21(DE3)-Glusor-3-C352D | *E. coli* BL21(DE3) carrying plasmid pETDuet-muta-C352D |
| *E. coli* BL21(DE3)-Glusor-3-C352E | *E. coli* BL21(DE3) carrying plasmid pETDuet-muta-C352E |
| *E. coli* BL21(DE3)-Glusor-3-C352F | *E. coli* BL21(DE3) carrying plasmid pETDuet-muta-C352F |
| *E. coli* BL21(DE3)-Glusor-3-C352G | *E. coli* BL21(DE3) carrying plasmid pETDuet-muta-C352G |
| *E. coli* BL21(DE3)-Glusor-3-C352H | *E. coli* BL21(DE3) carrying plasmid pETDuet-muta-C352H |
| *E. coli* BL21(DE3)-Glusor-3-C352I | *E. coli* BL21(DE3) carrying plasmid pETDuet-muta-C352I |
| *E. coli* BL21(DE3)-Glusor-3-C352K | *E. coli* BL21(DE3) carrying plasmid pETDuet-muta-C352K |
| *E. coli* BL21(DE3)-Glusor-3-C352L | *E. coli* BL21(DE3) carrying plasmid pETDuet-muta-C352L |
| *E. coli* BL21(DE3)-Glusor-3-C352M | *E. coli* BL21(DE3) carrying plasmid pETDuet-muta-C352M |
| *E. coli* BL21(DE3)-Glusor-3-C352N | *E. coli* BL21(DE3) carrying plasmid pETDuet-muta-C352N |
| *E. coli* BL21(DE3)-Glusor-3-C352P | *E. coli* BL21(DE3) carrying plasmid pETDuet-muta-C352P |
| *E. coli* BL21(DE3)-Glusor-3-C352Q | *E. coli* BL21(DE3) carrying plasmid pETDuet-muta-C352Q |
| *E. coli* BL21(DE3)-Glusor-3-C352R | *E. coli* BL21(DE3) carrying plasmid pETDuet-muta-C352R |
| *E. coli* BL21(DE3)-Glusor-3-C352S | *E. coli* BL21(DE3) carrying plasmid pETDuet-muta-C352S |
| *E. coli* BL21(DE3)-Glusor-3-C352T | *E. coli* BL21(DE3) carrying plasmid pETDuet-muta-C352T |
| *E. coli* BL21(DE3)-Glusor-3-C352V | *E. coli* BL21(DE3) carrying plasmid pETDuet-muta-C352V |
| *E. coli* BL21(DE3)-Glusor-3-C352W | *E. coli* BL21(DE3) carrying plasmid pETDuet-muta-C352W |
| *E. coli* BL21(DE3)-Glusor-3-C352Y | *E. coli* BL21(DE3) carrying plasmid pETDuet-muta-C352Y |
| *E. coli* BL21(DE3)-Glusor-3-S101A, G350K | *E. coli* BL21(DE3) carrying plasmid pETDuet-muta-S101A, G350K |
| *E. coli* BL21(DE3)-Glusor-3-S101A, G350N (Glusor) | *E. coli* BL21(DE3) carrying plasmid pETDuet-muta-S101A, G350N (Glusor) |
| *E. coli* BL21(DE3)-Glusor-3-S101A, G350Q | *E. coli* BL21(DE3) carrying plasmid pETDuet-muta-S101A, G350Q |
| *E. coli* BL21(DE3)-Glusor-3-S101A, G350R | *E. coli* BL21(DE3) carrying plasmid pETDuet-muta-S101A, G350R |
| *E. coli* BL21(DE3)-Glusor-3-S101A, G351A | *E. coli* BL21(DE3) carrying plasmid pETDuet-muta-S101A, G351A |
| *E. coli* BL21(DE3)-Glusor-3-S101A, G351C | *E. coli* BL21(DE3) carrying plasmid pETDuet-muta-S101A, G351C |
| *E. coli* BL21(DE3)-Glusor-3-S101A, C352A | *E. coli* BL21(DE3) carrying plasmid pETDuet-muta-S101A, C352A |
| *E. coli* BL21(DE3)-Glusor-3-S101A, C352E | *E. coli* BL21(DE3) carrying plasmid pETDuet-muta-S101A, C352E |
| *E. coli* BL21(DE3)-Glusor-3-S101A, C352I | *E. coli* BL21(DE3) carrying plasmid pETDuet-muta-S101A, C352I |
| *E. coli* BL21(DE3)-Glusor-3-S101A, C352N | *E. coli* BL21(DE3) carrying plasmid pETDuet-muta-S101A, C352N |
| *E. coli* BL21(DE3)-Glusor-3-S101A, C352P | *E. coli* BL21(DE3) carrying plasmid pETDuet-muta-S101A, C352P |
| *E. coli* BL21(DE3)-Glusor-3-S101A, C352S | *E. coli* BL21(DE3) carrying plasmid pETDuet-muta-S101A, C352S |
| *E. coli* BL21(DE3)-Glusor-3-S101A, C352V | *E. coli* BL21(DE3) carrying plasmid pETDuet-muta-S101A, C352V |
| *E. coli* BL21(DE3)-Glusor-3-S101A, C352Y | *E. coli* BL21(DE3) carrying plasmid pETDuet-muta-S101A, C352Y |
| *E. coli* BL21(DE3)-Glusor-3-S101G, G350K | *E. coli* BL21(DE3) carrying plasmid pETDuet-muta-S101G, G350K |
| *E. coli* BL21(DE3)-Glusor-3-S101G, G350N | *E. coli* BL21(DE3) carrying plasmid pETDuet-muta-S101G, G350N |
| *E. coli* BL21(DE3)-Glusor-3-S101G, G350Q | *E. coli* BL21(DE3) carrying plasmid pETDuet-muta-S101G, G350Q |
| *E. coli* BL21(DE3)-Glusor-3-S101G, G350R | *E. coli* BL21(DE3) carrying plasmid pETDuet-muta-S101G, G350R |
| *E. coli* BL21(DE3)-Glusor-3-S101G, G351A | *E. coli* BL21(DE3) carrying plasmid pETDuet-muta-S101G, G351A |
| *E. coli* BL21(DE3)-Glusor-3-S101G, G351C | *E. coli* BL21(DE3) carrying plasmid pETDuet-muta-S101G, G351C |
| *E. coli* BL21(DE3)-Glusor-3-S101G, C352A | *E. coli* BL21(DE3) carrying plasmid pETDuet-muta-S101G, C352A |
| *E. coli* BL21(DE3)-Glusor-3-S101G, C352E | *E. coli* BL21(DE3) carrying plasmid pETDuet-muta-S101G, C352E |
| *E. coli* BL21(DE3)-Glusor-3-S101G, C352I | *E. coli* BL21(DE3) carrying plasmid pETDuet-muta-S101G, C352I |
| *E. coli* BL21(DE3)-Glusor-3-S101G, C352N | *E. coli* BL21(DE3) carrying plasmid pETDuet-muta-S101G, C352N |
| *E. coli* BL21(DE3)-Glusor-3-S101G, C352P | *E. coli* BL21(DE3) carrying plasmid pETDuet-muta-S101G, C352P |
| *E. coli* BL21(DE3)-Glusor-3-S101G, C352S | *E. coli* BL21(DE3) carrying plasmid pETDuet-muta-S101G, C352S |
| *E. coli* BL21(DE3)-Glusor-3-S101G, C352V | *E. coli* BL21(DE3) carrying plasmid pETDuet-muta-S101G, C352V |
| *E. coli* BL21(DE3)-Glusor-3-S101G, C352Y | *E. coli* BL21(DE3) carrying plasmid pETDuet-muta-S101G, C352Y |
| *E. coli* BL21(DE3)-Glusor-3-A102D, G350K | *E. coli* BL21(DE3) carrying plasmid pETDuet-muta-A102D, G350K |
| *E. coli* BL21(DE3)-Glusor-3-A102D, G350N | *E. coli* BL21(DE3) carrying plasmid pETDuet-muta-A102D, G350N |
| *E. coli* BL21(DE3)-Glusor-3-A102D, G350Q | *E. coli* BL21(DE3) carrying plasmid pETDuet-muta-A102D, G350Q |
| *E. coli* BL21(DE3)-Glusor-3-A102D, G350R | *E. coli* BL21(DE3) carrying plasmid pETDuet-muta-A102D, G350R |
| *E. coli* BL21(DE3)-Glusor-3-A102D, G351A | *E. coli* BL21(DE3) carrying plasmid pETDuet-muta-A102D, G351A |
| *E. coli* BL21(DE3)-Glusor-3-A102D, G351C | *E. coli* BL21(DE3) carrying plasmid pETDuet-muta-A102D, G351C |
| *E. coli* BL21(DE3)-Glusor-3-A102D, C352A | *E. coli* BL21(DE3) carrying plasmid pETDuet-muta-A102D, C352A |
| *E. coli* BL21(DE3)-Glusor-3-A102D, C352E | *E. coli* BL21(DE3) carrying plasmid pETDuet-muta-A102D, C352E |
| *E. coli* BL21(DE3)-Glusor-3-A102D, C352I | *E. coli* BL21(DE3) carrying plasmid pETDuet-muta-A102D, C352I |
| *E. coli* BL21(DE3)-Glusor-3-A102D, C352N | *E. coli* BL21(DE3) carrying plasmid pETDuet-muta-A102D, C352N |
| *E. coli* BL21(DE3)-Glusor-3-A102D, C352P | *E. coli* BL21(DE3) carrying plasmid pETDuet-muta-A102D, C352P |
| *E. coli* BL21(DE3)-Glusor-3-A102D, C352S | *E. coli* BL21(DE3) carrying plasmid pETDuet-muta-A102D, C352S |
| *E. coli* BL21(DE3)-Glusor-3-A102D, C352V | *E. coli* BL21(DE3) carrying plasmid pETDuet-muta-A102D, C352V |
| *E. coli* BL21(DE3)-Glusor-3-A102D, C352Y | *E. coli* BL21(DE3) carrying plasmid pETDuet-muta-A102D, C352Y |
| *E. coli* BL21(DE3)-Glusor-3-A102I, G350K | *E. coli* BL21(DE3) carrying plasmid pETDuet-muta-A102I, G350K |
| *E. coli* BL21(DE3)-Glusor-3-A102I, G350N | *E. coli* BL21(DE3) carrying plasmid pETDuet-muta-A102I, G350N |
| *E. coli* BL21(DE3)-Glusor-3-A102I, G350Q | *E. coli* BL21(DE3) carrying plasmid pETDuet-muta-A102I, G350Q |
| *E. coli* BL21(DE3)-Glusor-3-A102I, G350R | *E. coli* BL21(DE3) carrying plasmid pETDuet-muta-A102I, G350R |
| *E. coli* BL21(DE3)-Glusor-3-A102I, G351A | *E. coli* BL21(DE3) carrying plasmid pETDuet-muta-A102I, G351A |
| *E. coli* BL21(DE3)-Glusor-3-A102I, G351C | *E. coli* BL21(DE3) carrying plasmid pETDuet-muta-A102I, G351C |
| *E. coli* BL21(DE3)-Glusor-3-A102I, C352A | *E. coli* BL21(DE3) carrying plasmid pETDuet-muta-A102I, C352A |
| *E. coli* BL21(DE3)-Glusor-3-A102I, C352E | *E. coli* BL21(DE3) carrying plasmid pETDuet-muta-A102I, C352E |
| *E. coli* BL21(DE3)-Glusor-3-A102I, C352I | *E. coli* BL21(DE3) carrying plasmid pETDuet-muta-A102I, C352I |
| *E. coli* BL21(DE3)-Glusor-3-A102I, C352N | *E. coli* BL21(DE3) carrying plasmid pETDuet-muta-A102I, C352N |
| *E. coli* BL21(DE3)-Glusor-3-A102I, C352P | *E. coli* BL21(DE3) carrying plasmid pETDuet-muta-A102I, C352P |
| *E. coli* BL21(DE3)-Glusor-3-A102I, C352S | *E. coli* BL21(DE3) carrying plasmid pETDuet-muta-A102I, C352S |
| *E. coli* BL21(DE3)-Glusor-3-A102I, C352V | *E. coli* BL21(DE3) carrying plasmid pETDuet-muta-A102I, C352V |
| *E. coli* BL21(DE3)-Glusor-3-A102I, C352Y | *E. coli* BL21(DE3) carrying plasmid pETDuet-muta-A102I, C352Y |
| *E. coli* BL21(DE3)-Glusor-3-A102M, G350K | *E. coli* BL21(DE3) carrying plasmid pETDuet-muta-A102M, G350K |
| *E. coli* BL21(DE3)-Glusor-3-A102M, G350N | *E. coli* BL21(DE3) carrying plasmid pETDuet-muta-A102M, G350N |
| *E. coli* BL21(DE3)-Glusor-3-A102M, G350Q | *E. coli* BL21(DE3) carrying plasmid pETDuet-muta-A102M, G350Q |
| *E. coli* BL21(DE3)-Glusor-3-A102M, G350R | *E. coli* BL21(DE3) carrying plasmid pETDuet-muta-A102M, G350R |
| *E. coli* BL21(DE3)-Glusor-3-A102M, G351A | *E. coli* BL21(DE3) carrying plasmid pETDuet-muta-A102M, G351A |
| *E. coli* BL21(DE3)-Glusor-3-A102M, G351C | *E. coli* BL21(DE3) carrying plasmid pETDuet-muta-A102M, G351C |
| *E. coli* BL21(DE3)-Glusor-3-A102M, C352A | *E. coli* BL21(DE3) carrying plasmid pETDuet-muta-A102M, C352A |
| *E. coli* BL21(DE3)-Glusor-3-A102M, C352E | *E. coli* BL21(DE3) carrying plasmid pETDuet-muta-A102M, C352E |
| *E. coli* BL21(DE3)-Glusor-3-A102M, C352I | *E. coli* BL21(DE3) carrying plasmid pETDuet-muta-A102M, C352I |
| *E. coli* BL21(DE3)-Glusor-3-A102M, C352N | *E. coli* BL21(DE3) carrying plasmid pETDuet-muta-A102M, C352N |
| *E. coli* BL21(DE3)-Glusor-3-A102M, C352P | *E. coli* BL21(DE3) carrying plasmid pETDuet-muta-A102M, C352P |
| *E. coli* BL21(DE3)-Glusor-3-A102M, C352S | *E. coli* BL21(DE3) carrying plasmid pETDuet-muta-A102M, C352S |
| *E. coli* BL21(DE3)-Glusor-3-A102M, C352V | *E. coli* BL21(DE3) carrying plasmid pETDuet-muta-A102M, C352V |
| *E. coli* BL21(DE3)-Glusor-3-A102M, C352Y | *E. coli* BL21(DE3) carrying plasmid pETDuet-muta-A102M, C352Y |
| *E. coli* BL21(DE3)-Glusor-3-A102Q, G350K | *E. coli* BL21(DE3) carrying plasmid pETDuet-muta-A102Q, G350K |
| *E. coli* BL21(DE3)-Glusor-3-A102Q, G350N | *E. coli* BL21(DE3) carrying plasmid pETDuet-muta-A102Q, G350N |
| *E. coli* BL21(DE3)-Glusor-3-A102Q, G350Q | *E. coli* BL21(DE3) carrying plasmid pETDuet-muta-A102Q, G350Q |
| *E. coli* BL21(DE3)-Glusor-3-A102Q, G350R | *E. coli* BL21(DE3) carrying plasmid pETDuet-muta-A102Q, G350R |
| *E. coli* BL21(DE3)-Glusor-3-A102Q, G351A | *E. coli* BL21(DE3) carrying plasmid pETDuet-muta-A102Q, G351A |
| *E. coli* BL21(DE3)-Glusor-3-A102Q, G351C | *E. coli* BL21(DE3) carrying plasmid pETDuet-muta-A102Q, G351C |
| *E. coli* BL21(DE3)-Glusor-3-A102Q, C352A | *E. coli* BL21(DE3) carrying plasmid pETDuet-muta-A102Q, C352A |
| *E. coli* BL21(DE3)-Glusor-3-A102Q, C352E | *E. coli* BL21(DE3) carrying plasmid pETDuet-muta-A102Q, C352E |
| *E. coli* BL21(DE3)-Glusor-3-A102Q, C352I | *E. coli* BL21(DE3) carrying plasmid pETDuet-muta-A102Q, C352I |
| *E. coli* BL21(DE3)-Glusor-3-A102Q, C352N | *E. coli* BL21(DE3) carrying plasmid pETDuet-muta-A102Q, C352N |
| *E. coli* BL21(DE3)-Glusor-3-A102Q, C352P | *E. coli* BL21(DE3) carrying plasmid pETDuet-muta-A102Q, C352P |
| *E. coli* BL21(DE3)-Glusor-3-A102Q, C352S | *E. coli* BL21(DE3) carrying plasmid pETDuet-muta-A102Q, C352S |
| *E. coli* BL21(DE3)-Glusor-3-A102Q, C352V | *E. coli* BL21(DE3) carrying plasmid pETDuet-muta-A102Q, C352V |
| *E. coli* BL21(DE3)-Glusor-3-A102Q, C352Y | *E. coli* BL21(DE3) carrying plasmid pETDuet-muta-A102Q, C352Y |
| *E. coli* BL21(DE3)-Glusor-3-A102S, G350K | *E. coli* BL21(DE3) carrying plasmid pETDuet-muta-A102S, G350K |
| *E. coli* BL21(DE3)-Glusor-3-A102S, G350N | *E. coli* BL21(DE3) carrying plasmid pETDuet-muta-A102S, G350N |
| *E. coli* BL21(DE3)-Glusor-3-A102S, G350Q | *E. coli* BL21(DE3) carrying plasmid pETDuet-muta-A102S, G350Q |
| *E. coli* BL21(DE3)-Glusor-3-A102S, G350R | *E. coli* BL21(DE3) carrying plasmid pETDuet-muta-A102S, G350R |
| *E. coli* BL21(DE3)-Glusor-3-A102S, G351A | *E. coli* BL21(DE3) carrying plasmid pETDuet-muta-A102S, G351A |
| *E. coli* BL21(DE3)-Glusor-3-A102S, G351C | *E. coli* BL21(DE3) carrying plasmid pETDuet-muta-A102S, G351C |
| *E. coli* BL21(DE3)-Glusor-3-A102S, C352A | *E. coli* BL21(DE3) carrying plasmid pETDuet-muta-A102S, C352A |
| *E. coli* BL21(DE3)-Glusor-3-A102S, C352E | *E. coli* BL21(DE3) carrying plasmid pETDuet-muta-A102S, C352E |
| *E. coli* BL21(DE3)-Glusor-3-A102S, C352I | *E. coli* BL21(DE3) carrying plasmid pETDuet-muta-A102S, C352I |
| *E. coli* BL21(DE3)-Glusor-3-A102S, C352N | *E. coli* BL21(DE3) carrying plasmid pETDuet-muta-A102S, C352N |
| *E. coli* BL21(DE3)-Glusor-3-A102S, C352P | *E. coli* BL21(DE3) carrying plasmid pETDuet-muta-A102S, C352P |
| *E. coli* BL21(DE3)-Glusor-3-A102S, C352S | *E. coli* BL21(DE3) carrying plasmid pETDuet-muta-A102S, C352S |
| *E. coli* BL21(DE3)-Glusor-3-A102S, C352V | *E. coli* BL21(DE3) carrying plasmid pETDuet-muta-A102S, C352V |
| *E. coli* BL21(DE3)-Glusor-3-A102S, C352Y | *E. coli* BL21(DE3) carrying plasmid pETDuet-muta-A102S, C352Y |
| *E. coli* BL21(DE3)-Glusor-3-A102T, G350K | *E. coli* BL21(DE3) carrying plasmid pETDuet-muta-A102T, G350K |
| *E. coli* BL21(DE3)-Glusor-3-A102T, G350N | *E. coli* BL21(DE3) carrying plasmid pETDuet-muta-A102T, G350N |
| *E. coli* BL21(DE3)-Glusor-3-A102T, G350Q | *E. coli* BL21(DE3) carrying plasmid pETDuet-muta-A102T, G350Q |
| *E. coli* BL21(DE3)-Glusor-3-A102T, G350R | *E. coli* BL21(DE3) carrying plasmid pETDuet-muta-A102T, G350R |
| *E. coli* BL21(DE3)-Glusor-3-A102T, G351A | *E. coli* BL21(DE3) carrying plasmid pETDuet-muta-A102T, G351A |
| *E. coli* BL21(DE3)-Glusor-3-A102T, G351C | *E. coli* BL21(DE3) carrying plasmid pETDuet-muta-A102T, G351C |
| *E. coli* BL21(DE3)-Glusor-3-A102T, C352A | *E. coli* BL21(DE3) carrying plasmid pETDuet-muta-A102T, C352A |
| *E. coli* BL21(DE3)-Glusor-3-A102T, C352E | *E. coli* BL21(DE3) carrying plasmid pETDuet-muta-A102T, C352E |
| *E. coli* BL21(DE3)-Glusor-3-A102T, C352I | *E. coli* BL21(DE3) carrying plasmid pETDuet-muta-A102T, C352I |
| *E. coli* BL21(DE3)-Glusor-3-A102T, C352N | *E. coli* BL21(DE3) carrying plasmid pETDuet-muta-A102T, C352N |
| *E. coli* BL21(DE3)-Glusor-3-A102T, C352P | *E. coli* BL21(DE3) carrying plasmid pETDuet-muta-A102T, C352P |
| *E. coli* BL21(DE3)-Glusor-3-A102T, C352S | *E. coli* BL21(DE3) carrying plasmid pETDuet-muta-A102T, C352S |
| *E. coli* BL21(DE3)-Glusor-3-A102T, C352V | *E. coli* BL21(DE3) carrying plasmid pETDuet-muta-A102T, C352V |
| *E. coli* BL21(DE3)-Glusor-3-A102T, C352Y | *E. coli* BL21(DE3) carrying plasmid pETDuet-muta-A102T, C352Y |
| *E. coli* BL21(DE3)-Glusor-3-A102V, G350K | *E. coli* BL21(DE3) carrying plasmid pETDuet-muta-A102V, G350K |
| *E. coli* BL21(DE3)-Glusor-3-A102V, G350N | *E. coli* BL21(DE3) carrying plasmid pETDuet-muta-A102V, G350N |
| *E. coli* BL21(DE3)-Glusor-3-A102V, G350Q | *E. coli* BL21(DE3) carrying plasmid pETDuet-muta-A102V, G350Q |
| *E. coli* BL21(DE3)-Glusor-3-A102V, G350R | *E. coli* BL21(DE3) carrying plasmid pETDuet-muta-A102V, G350R |
| *E. coli* BL21(DE3)-Glusor-3-A102V, G351A | *E. coli* BL21(DE3) carrying plasmid pETDuet-muta-A102V, G351A |
| *E. coli* BL21(DE3)-Glusor-3-A102V, G351C | *E. coli* BL21(DE3) carrying plasmid pETDuet-muta-A102V, G351C |
| *E. coli* BL21(DE3)-Glusor-3-A102V, C352A | *E. coli* BL21(DE3) carrying plasmid pETDuet-muta-A102V, C352A |
| *E. coli* BL21(DE3)-Glusor-3-A102V, C352E | *E. coli* BL21(DE3) carrying plasmid pETDuet-muta-A102V, C352E |
| *E. coli* BL21(DE3)-Glusor-3-A102V, C352I | *E. coli* BL21(DE3) carrying plasmid pETDuet-muta-A102V, C352I |
| *E. coli* BL21(DE3)-Glusor-3-A102V, C352N | *E. coli* BL21(DE3) carrying plasmid pETDuet-muta-A102V, C352N |
| *E. coli* BL21(DE3)-Glusor-3-A102V, C352P | *E. coli* BL21(DE3) carrying plasmid pETDuet-muta-A102V, C352P |
| *E. coli* BL21(DE3)-Glusor-3-A102V, C352S | *E. coli* BL21(DE3) carrying plasmid pETDuet-muta-A102V, C352S |
| *E. coli* BL21(DE3)-Glusor-3-A102V, C352V | *E. coli* BL21(DE3) carrying plasmid pETDuet-muta-A102V, C352V |
| *E. coli* BL21(DE3)-Glusor-3-A102V, C352Y | *E. coli* BL21(DE3) carrying plasmid pETDuet-muta-A102V, C352Y |
| *E. coli* BL21(DE3)-Glusor-3-G103Q, G350K | *E. coli* BL21(DE3) carrying plasmid pETDuet-muta-G103Q, G350K |
| *E. coli* BL21(DE3)-Glusor-3-G103Q, G350N | *E. coli* BL21(DE3) carrying plasmid pETDuet-muta-G103Q, G350N |
| *E. coli* BL21(DE3)-Glusor-3-G103Q, G350Q | *E. coli* BL21(DE3) carrying plasmid pETDuet-muta-G103Q, G350Q |
| *E. coli* BL21(DE3)-Glusor-3-G103Q, G350R | *E. coli* BL21(DE3) carrying plasmid pETDuet-muta-G103Q, G350R |
| *E. coli* BL21(DE3)-Glusor-3-G103Q, G351A | *E. coli* BL21(DE3) carrying plasmid pETDuet-muta-G103Q, G351A |
| *E. coli* BL21(DE3)-Glusor-3-G103Q, G351C | *E. coli* BL21(DE3) carrying plasmid pETDuet-muta-G103Q, G351C |
| *E. coli* BL21(DE3)-Glusor-3-G103Q, C352A | *E. coli* BL21(DE3) carrying plasmid pETDuet-muta-G103Q, C352A |
| *E. coli* BL21(DE3)-Glusor-3-G103Q, C352E | *E. coli* BL21(DE3) carrying plasmid pETDuet-muta-G103Q, C352E |
| *E. coli* BL21(DE3)-Glusor-3-G103Q, C352I | *E. coli* BL21(DE3) carrying plasmid pETDuet-muta-G103Q, C352I |
| *E. coli* BL21(DE3)-Glusor-3-G103Q, C352N | *E. coli* BL21(DE3) carrying plasmid pETDuet-muta-G103Q, C352N |
| *E. coli* BL21(DE3)-Glusor-3-G103Q, C352P | *E. coli* BL21(DE3) carrying plasmid pETDuet-muta-G103Q, C352P |
| *E. coli* BL21(DE3)-Glusor-3-G103Q, C352S | *E. coli* BL21(DE3) carrying plasmid pETDuet-muta-G103Q, C352S |
| *E. coli* BL21(DE3)-Glusor-3-G103Q, C352V | *E. coli* BL21(DE3) carrying plasmid pETDuet-muta-G103Q, C352V |
| *E. coli* BL21(DE3)-Glusor-3-G103Q, C352Y | *E. coli* BL21(DE3) carrying plasmid pETDuet-muta-G103Q, C352Y |
| *E. coli* BL21(DE3)-Glusor-3-G103S, G350K | *E. coli* BL21(DE3) carrying plasmid pETDuet-muta-G103S, G350K |
| *E. coli* BL21(DE3)-Glusor-3-G103S, G350N | *E. coli* BL21(DE3) carrying plasmid pETDuet-muta-G103S, G350N |
| *E. coli* BL21(DE3)-Glusor-3-G103S, G350Q | *E. coli* BL21(DE3) carrying plasmid pETDuet-muta-G103S, G350Q |
| *E. coli* BL21(DE3)-Glusor-3-G103S, G350R | *E. coli* BL21(DE3) carrying plasmid pETDuet-muta-G103S, G350R |
| *E. coli* BL21(DE3)-Glusor-3-G103S, G351A | *E. coli* BL21(DE3) carrying plasmid pETDuet-muta-G103S, G351A |
| *E. coli* BL21(DE3)-Glusor-3-G103S, G351C | *E. coli* BL21(DE3) carrying plasmid pETDuet-muta-G103S, G351C |
| *E. coli* BL21(DE3)-Glusor-3-G103S, C352A | *E. coli* BL21(DE3) carrying plasmid pETDuet-muta-G103S, C352A |
| *E. coli* BL21(DE3)-Glusor-3-G103S, C352E | *E. coli* BL21(DE3) carrying plasmid pETDuet-muta-G103S, C352E |
| *E. coli* BL21(DE3)-Glusor-3-G103S, C352I | *E. coli* BL21(DE3) carrying plasmid pETDuet-muta-G103S, C352I |
| *E. coli* BL21(DE3)-Glusor-3-G103S, C352N | *E. coli* BL21(DE3) carrying plasmid pETDuet-muta-G103S, C352N |
| *E. coli* BL21(DE3)-Glusor-3-G103S, C352P | *E. coli* BL21(DE3) carrying plasmid pETDuet-muta-G103S, C352P |
| *E. coli* BL21(DE3)-Glusor-3-G103S, C352S | *E. coli* BL21(DE3) carrying plasmid pETDuet-muta-G103S, C352S |
| *E. coli* BL21(DE3)-Glusor-3-G103S, C352V | *E. coli* BL21(DE3) carrying plasmid pETDuet-muta-G103S, C352V |
| *E. coli* BL21(DE3)-Glusor-3-G103S, C352Y | *E. coli* BL21(DE3) carrying plasmid pETDuet-muta-G103S, C352Y |
| *E. coli* BL21(DE3)-Glusor | *E. coli* BL21(DE3) carrying plasmid pETDuet-Glusor |
| *E. coli* BL21(DE3)-cpSFYFP | *E. coli* BL21(DE3) carrying plasmid pETDuet-cpSFYFP |
| *E. coli* BL21(DE3)-Glusor^Y84A^ | *E. coli* BL21(DE3) carrying plasmid pETDuet-Glusor^Y84A^ |
| *E. coli* BL21(DE3)-Glusor^R87A^ | *E. coli* BL21(DE3) carrying plasmid pETDuet-Glusor^R87A^ |
| *E. coli* BL21(DE3)-Glusor^H137A^ | *E. coli* BL21(DE3) carrying plasmid pETDuet-Glusor^H137A^ |
| *E. coli* BL21(DE3)-Glusor^H141A^ | *E. coli* BL21(DE3) carrying plasmid pETDuet-Glusor^H141A^ |
| *E. coli* BL21(DE3)-Glusor^R168A^ | *E. coli* BL21(DE3) carrying plasmid pETDuet-Glusor^R168A^ |
| *E. coli* BL21(DE3)-Glusor^H189A^ | *E. coli* BL21(DE3) carrying plasmid pETDuet-Glusor^H189A^ |
| *E. coli* BL21(DE3)-Glusor^H211A^ | *E. coli* BL21(DE3) carrying plasmid pETDuet-Glusor^H211A^ |
| *P. putida* KT2440 | Wild-type |
| *P. putida* KT2440 (∆*csiD*) | *P. putida* KT2440 lacking gene *csiD* |
| *P. putida* KT2440 (∆*gcdH*) | *P. putida* KT2440 lacking gene *gcdH* |
| *P. putida* KT2440 (∆*csiD*∆*gcdH*) | *P. putida* KT2440 lacking genes *csiD* and *gcdH* |
| *P. putida* KT2440 (∆*csiD*∆*gcdH*∆*alr*) | *P. putida* KT2440 lacking genes *csiD*, *gcdH*, and *alr* |
| *P. putida* KT2440 (∆*lhgO*) | *P. putida* KT2440 lacking gene *lhgO* |
| *P. putida* KT2440 (∆*csiD*∆*lhgO*) | *P. putida* KT2440 lacking genes *csiD* and *lhgO* |
| *E. coli* BL21(DE3)-pEcCas | *E. coli* BL21(DE3) carrying plasmid pEcCas |
| *E. coli* BL21(DE3)-pEcgRNA | *E. coli* BL21(DE3) carrying plasmid pEcgRNA |
| *E. coli* BL21(DE3)-pEcgRNA-N20-*csiD* | *E. coli* BL21(DE3) carrying plasmid pEcgRNA-N20-*csiD* |
| *E. coli* BL21(DE3)-pEcgRNA-N20-*kgtP* | *E. coli* BL21(DE3) carrying plasmid pEcgRNA-N20-*kgtP* |
| *E. coli* BL21(DE3)-pEcgRNA-N20-*dctA* | *E. coli* BL21(DE3) carrying plasmid pEcgRNA-N20-*dctA* |
| *E. coli* BL21(DE3)-pEcgRNA-N20-*dauA* | *E. coli* BL21(DE3) carrying plasmid pEcgRNA-N20-*dauA* |
| *E. coli* BL21(DE3)-pEcgRNA-N20-*dcuA* | *E. coli* BL21(DE3) carrying plasmid pEcgRNA-N20-*dcuA* |
| *E. coli* BL21(DE3)-pEcgRNA-N20-*ynfM* | *E. coli* BL21(DE3) carrying plasmid pEcgRNA-N20-*ynfM* |
| *E. coli* BL21(DE3)-pEcCas-pEcgRNA-N20-*csiD* | *E. coli* BL21(DE3) carrying plasmids pEcCas and pEcgRNA-N20-*csiD* |
| *E. coli* BL21(DE3)-pEcCas-pEcgRNA-N20-*kgtP* | *E. coli* BL21(DE3) carrying plasmids pEcCas and pEcgRNA-N20-*kgtP* |
| *E. coli* BL21(DE3)-pEcCas-pEcgRNA-N20-*dctA* | *E. coli* BL21(DE3) carrying plasmids pEcCas and pEcgRNA-N20-*dctA* |
| *E. coli* BL21(DE3)-pEcCas-pEcgRNA-N20-*dauA* | *E. coli* BL21(DE3) carrying plasmids pEcCas and pEcgRNA-N20-*dauA* |
| *E. coli* BL21(DE3)-pEcCas-pEcgRNA-N20-*dcuA* | *E. coli* BL21(DE3) carrying plasmids pEcCas and pEcgRNA-N20-*dcuA* |
| *E. coli* BL21(DE3)-pEcCas-pEcgRNA-N20-*ynfM* | *E. coli* BL21(DE3) carrying plasmids pEcCas and pEcgRNA-N20-*ynfM* |
| *E. coli* BL21(DE3) (∆*csiD*) | *E. coli* BL21(DE3) lacking gene *csiD* |
| *E. coli* BL21(DE3) (∆*kgtP*) | *E. coli* BL21(DE3) lacking gene *kgtP* |
| *E. coli* BL21(DE3) (∆*dctA*) | *E. coli* BL21(DE3) lacking gene *dctA* |
| *E. coli* BL21(DE3) (∆*dauA*) | *E. coli* BL21(DE3) lacking gene *dauA* |
| *E. coli* BL21(DE3) (∆*dcuA*) | *E. coli* BL21(DE3) lacking gene *dcuA* |
| *E. coli* BL21(DE3) (∆*ynfM*) | *E. coli* BL21(DE3) lacking gene *ynfM* |
| *E. coli* BL21(DE3) (∆*csiD*)-pETDuet-Glusor | *E. coli* BL21(DE3) (∆*csiD*) carrying plasmid pETDuet-Glusor |
| *E. coli* BL21(DE3) (∆*csiD*)-pETDuet-cpSFYFP | *E. coli* BL21(DE3) (∆*csiD*) carrying plasmid pETDuet-cpSFYFP |
| *E. coli* BL21(DE3) (∆*kgtP*)-pETDuet-Glusor | *E. coli* BL21(DE3) (∆*kgtP*) carrying plasmid pETDuet-Glusor |
| *E. coli* BL21(DE3) (∆*kgtP*)-pETDuet-cpSFYFP | *E. coli* BL21(DE3) (∆*kgtP*) carrying plasmid pETDuet-cpSFYFP |
| *E. coli* BL21(DE3) (∆*dctA*)-pETDuet-Glusor | *E. coli* BL21(DE3) (∆*dctA*) carrying plasmid pETDuet-Glusor |
| *E. coli* BL21(DE3) (∆*dctA*)-pETDuet-cpSFYFP | *E. coli* BL21(DE3) (∆*dctA*) carrying plasmid pETDuet-cpSFYFP |
| *E. coli* BL21(DE3) (∆*dauA*)-pETDuet-Glusor | *E. coli* BL21(DE3) (∆*dauA*) carrying plasmid pETDuet-Glusor |
| *E. coli* BL21(DE3) (∆*dauA*)-pETDuet-cpSFYFP | *E. coli* BL21(DE3) (∆*dauA*) carrying plasmid pETDuet-cpSFYFP |
| *E. coli* BL21(DE3) (∆*dcuA*)-pETDuet-Glusor | *E. coli* BL21(DE3) (∆*dcuA*) carrying plasmid pETDuet-Glusor |
| *E. coli* BL21(DE3) (∆*dcuA*)-pETDuet-cpSFYFP | *E. coli* BL21(DE3) (∆*dcuA*) carrying plasmid pETDuet-cpSFYFP |
| *E. coli* BL21(DE3) (∆*ynfM*)-pETDuet-Glusor | *E. coli* BL21(DE3) (∆*ynfM*) carrying plasmid pETDuet-Glusor |
| *E. coli* BL21(DE3) (∆*ynfM*)-pETDuet-cpSFYFP | *E. coli* BL21(DE3) (∆*ynfM*) carrying plasmid pETDuet-cpSFYFP |
| *E. coli* DH5α*-*pcDNA3.1^(+)^-Glusor | *E. coli* DH5α carrying mammalian expression plasmid pcDNA3.1^(+)^-Glusor |
| *E. coli* DH5α*-*pcDNA3.1^(+)^-Glusor-Mito | *E. coli* DH5α carrying mammalian expression plasmid pcDNA3.1^(+)^-Glusor-Mito |
| *E. coli* DH5α*-*pcDNA3.1^(+)^-Glusor-Cyto | *E. coli* DH5α carrying mammalian expression plasmid pcDNA3.1^(+)^-Glusor-Cyto |
| *E. coli* DH5α*-*pcDNA3.1^(+)^-Glusor-Nuc | *E. coli* DH5α carrying mammalian expression plasmid pcDNA3.1^(+)^-Glusor-Nuc |
| *E. coli* DH5α*-*pcDNA3.1^(+)^-cpSFYFP | *E. coli* DH5α carrying mammalian expression plasmid pcDNA3.1^(+)^-cpSFYFP |
| *E. coli* DH5α*-*pcDNA3.1^(+)^-cpSFYFP-Mito | *E. coli* DH5α carrying mammalian expression plasmid pcDNA3.1^(+)^-cpSFYFP-Mito |
| *E. coli* DH5α*-*pcDNA3.1^(+)^-cpSFYFP-Cyto | *E. coli* DH5α carrying mammalian expression plasmid pcDNA3.1^(+)^-cpSFYFP-Cyto |
| *E. coli* DH5α*-*pcDNA3.1^(+)^-cpSFYFP-Nuc | *E. coli* DH5α carrying mammalian expression plasmid pcDNA3.1^(+)^-cpSFYFP-Nuc |
| *E. coli* DH5α*-*pcDNA3.1^(+)^-SLC22A6 | *E. coli* DH5α carrying mammalian expression plasmid pcDNA3.1^(+)^-SLC22A6 |
| **Plasmid** | |
| pETDuet-1 | Vector for protein expression in bacteria; Ap^r^ |
| pETDuet-CsiR | pETDuet-1 contained the gene of CsiR |
| pETDuet-CsiR-LBD | pETDuet-1 contained the gene of CsiR-LBD |
| pETDuet-CsiR-LBD^Y84A^ | pETDuet-1 contained the gene of CsiR-LBD^Y84A^ |
| pETDuet-CsiR-LBD^R87A^ | pETDuet-1 contained the gene of CsiR-LBD^R87A^ |
| pETDuet-CsiR-LBD^H137A^ | pETDuet-1 contained the gene of CsiR-LBD^H137A^ |
| pETDuet-CsiR-LBD^H141A^ | pETDuet-1 contained the gene of CsiR-LBD^H141A^ |
| pETDuet-CsiR-LBD^R168A^ | pETDuet-1 contained the gene of CsiR-LBD^R168A^ |
| pETDuet-CsiR-LBD^H189A^ | pETDuet-1 contained the gene of CsiR-LBD^H189A^ |
| pETDuet-CsiR-LBD^H211A^ | pETDuet-1 contained the gene of CsiR-LBD^H211A^ |
| pETDuet-SF-CsiR_231R/_ | pETDuet-1 contained the gene of SF-CsiR_231R/_ |
| pETDuet-SF-CsiR_226E/227G_ | pETDuet-1 contained the gene of SF-CsiR_226E/227G_ |
| pETDuet-SF-CsiR_225A/226E_ (Glusor-1) | pETDuet-1 contained the gene of SF-CsiR_225A/226E_ (Glusor-1) |
| pETDuet-SF-CsiR_215P/215V_ | pETDuet-1 contained the gene of SF-CsiR_215P/215V_ |
| pETDuet-SF-CsiR_214T/215P_ | pETDuet-1 contained the gene of SF-CsiR_214T/215P_ |
| pETDuet-SF-CsiR_213M/214T_ | pETDuet-1 contained the gene of SF-CsiR_213M/214T_ |
| pETDuet-SF-CsiR_212L/213M_ | pETDuet-1 contained the gene of SF-CsiR_212L/213M_ |
| pETDuet-SF-CsiR_211H/212L_ | pETDuet-1 contained the gene of SF-CsiR_211H/212L_ |
| pETDuet-SF-CsiR_210S/211H_ | pETDuet-1 contained the gene of SF-CsiR_210S/211H_ |
| pETDuet-SF-CsiR_200D/201A_ | pETDuet-1 contained the gene of SF-CsiR_200D/201A_ |
| pETDuet-SF-CsiR_199R/200D_ | pETDuet-1 contained the gene of SF-CsiR_199R/200D_ |
| pETDuet-SF-CsiR_198A/199R_ | pETDuet-1 contained the gene of SF-CsiR_198A/199R_ |
| pETDuet-SF-CsiR_197L/198A_ | pETDuet-1 contained the gene of SF-CsiR_197L/198A_ |
| pETDuet-SF-CsiR_178S/179E_ | pETDuet-1 contained the gene of SF-CsiR_178S/179E_ |
| pETDuet-SF-CsiR_177F/178S_ | pETDuet-1 contained the gene of SF-CsiR_177F/178S_ |
| pETDuet-SF-CsiR_176V/177F_ (Glusor-2) | pETDuet-1 contained the gene of SF-CsiR_176V/177F_ (Glusor-2) |
| pETDuet-SF-CsiR_175T/176V_ | pETDuet-1 contained the gene of SF-CsiR_175T/176V_ |
| pETDuet-SF-CsiR_174Q/175T_ | pETDuet-1 contained the gene of SF-CsiR_174Q/175T_ |
| pETDuet-SF-CsiR_173T/174Q_ | pETDuet-1 contained the gene of SF-CsiR_173T/174Q_ |
| pETDuet-SF-CsiR_150S/151K_ | pETDuet-1 contained the gene of SF-CsiR_150S/151K_ |
| pETDuet-SF-CsiR_149G/150S_ | pETDuet-1 contained the gene of SF-CsiR_149G/150S_ |
| pETDuet-SF-CsiR_148C/149G_ | pETDuet-1 contained the gene of SF-CsiR_148C/149G_ |
| pETDuet-SF-CsiR_147G/148C_ | pETDuet-1 contained the gene of SF-CsiR_147G/148C_ |
| pETDuet-SF-CsiR_146S/147G_ | pETDuet-1 contained the gene of SF-CsiR_146S/147G_ |
| pETDuet-SF-CsiR_145A/146S_ | pETDuet-1 contained the gene of SF-CsiR_145A/146S_ |
| pETDuet-SF-CsiR_129R/130L_ | pETDuet-1 contained the gene of SF-CsiR_129R/130L_ |
| pETDuet-SF-CsiR_128Q/129R_ | pETDuet-1 contained the gene of SF-CsiR_128Q/129R_ |
| pETDuet-SF-CsiR_127E/128Q_ | pETDuet-1 contained the gene of SF-CsiR_127E/128Q_ |
| pETDuet-SF-CsiR_126R/127E_ | pETDuet-1 contained the gene of SF-CsiR_126R/127E_ |
| pETDuet-SF-CsiR_125T/126R_ | pETDuet-1 contained the gene of SF-CsiR_125T/126R_ |
| pETDuet-SF-CsiR_124K/125T_ | pETDuet-1 contained the gene of SF-CsiR_124K/125T_ |
| pETDuet-SF-CsiR_123V/124K_ | pETDuet-1 contained the gene of SF-CsiR_123V/124K_ |
| pETDuet-SF-CsiR_122E/123V_ | pETDuet-1 contained the gene of SF-CsiR_122E/123V_ |
| pETDuet-SF-CsiR_121V/122E_ | pETDuet-1 contained the gene of SF-CsiR_121V/122E_ |
| pETDuet-SF-CsiR_120V/121V_ | pETDuet-1 contained the gene of SF-CsiR_120V/121V_ |
| pETDuet-SF-CsiR_119K/120V_ | pETDuet-1 contained the gene of SF-CsiR_119K/120V_ |
| pETDuet-SF-CsiR_118A/119K_ | pETDuet-1 contained the gene of SF-CsiR_118A/119K_ |
| pETDuet-SF-CsiR_113H/114S_ | pETDuet-1 contained the gene of SF-CsiR_113H/114S_ |
| pETDuet-SF-CsiR_112A/113H_ | pETDuet-1 contained the gene of SF-CsiR_112A/113H_ |
| pETDuet-SF-CsiR_104D/105A_ | pETDuet-1 contained the gene of SF-CsiR_104D/105A_ |
| pETDuet-SF-CsiR_103D/104D_ | pETDuet-1 contained the gene of SF-CsiR_103D/104D_ |
| pETDuet-SF-CsiR_102G/103D_ | pETDuet-1 contained the gene of SF-CsiR_102G/103D_ |
| pETDuet-SF-CsiR_101R/102G_ | pETDuet-1 contained the gene of SF-CsiR_101R/102G_ |
| pETDuet-SF-CsiR_100E/101R_ | pETDuet-1 contained the gene of SF-CsiR_100E/101R_ |
| pETDuet-SF-CsiR_76S/77L_ | pETDuet-1 contained the gene of SF-CsiR_76S/77L_ |
| pETDuet-SF-CsiR_75M/76S_ | pETDuet-1 contained the gene of SF-CsiR_75M/76S_ |
| pETDuet-SF-CsiR_74P/75M_ | pETDuet-1 contained the gene of SF-CsiR_74P/75M_ |
| pETDuet-SF-CsiR_73A/74P_ | pETDuet-1 contained the gene of SF-CsiR_73A/74P_ |
| pETDuet-SF-CsiR_72V/73A_ | pETDuet-1 contained the gene of SF-CsiR_72V/73A_ |
| pETDuet-SF-CsiR_71R/72V_ | pETDuet-1 contained the gene of SF-CsiR_71R/72V_ |
| pETDuet-SF-CsiR_/1M_ | pETDuet-1 contained the gene of SF-CsiR_/1M_ |
| pETDuet-SF-CsiR-15-nodbd (Glusor-3) | pETDuet-1 contained the gene of SF-CsiR-15-nodbd (Glusor-3) |
| pETDuet-muta-S101A | pETDuet-1 contained the gene of Glusor-3-S101A |
| pETDuet-muta-S101C | pETDuet-1 contained the gene of Glusor-3-S101C |
| pETDuet-muta-S101D | pETDuet-1 contained the gene of Glusor-3-S101D |
| pETDuet-muta-S101E | pETDuet-1 contained the gene of Glusor-3-S101E |
| pETDuet-muta-S101F | pETDuet-1 contained the gene of Glusor-3-S101F |
| pETDuet-muta-S101G | pETDuet-1 contained the gene of Glusor-3-S101G |
| pETDuet-muta-S101H | pETDuet-1 contained the gene of Glusor-3-S101H |
| pETDuet-muta-S101I | pETDuet-1 contained the gene of Glusor-3-S101I |
| pETDuet-muta-S101K | pETDuet-1 contained the gene of Glusor-3-S101K |
| pETDuet-muta-S101L | pETDuet-1 contained the gene of Glusor-3-S101L |
| pETDuet-muta-S101M | pETDuet-1 contained the gene of Glusor-3-S101M |
| pETDuet-muta-S101N | pETDuet-1 contained the gene of Glusor-3-S101N |
| pETDuet-muta-S101P | pETDuet-1 contained the gene of Glusor-3-S101P |
| pETDuet-muta-S101Q | pETDuet-1 contained the gene of Glusor-3-S101Q |
| pETDuet-muta-S101R | pETDuet-1 contained the gene of Glusor-3-S101R |
| pETDuet-muta-S101T | pETDuet-1 contained the gene of Glusor-3-S101T |
| pETDuet-muta-S101V | pETDuet-1 contained the gene of Glusor-3-S101V |
| pETDuet-muta-S101W | pETDuet-1 contained the gene of Glusor-3-S101W |
| pETDuet-muta-S101Y | pETDuet-1 contained the gene of Glusor-3-S101Y |
| pETDuet-muta-A102C | pETDuet-1 contained the gene of Glusor-3-A102C |
| pETDuet-muta-A102D | pETDuet-1 contained the gene of Glusor-3-A102D |
| pETDuet-muta-A102E | pETDuet-1 contained the gene of Glusor-3-A102E |
| pETDuet-muta-A102F | pETDuet-1 contained the gene of Glusor-3-A102F |
| pETDuet-muta-A102G | pETDuet-1 contained the gene of Glusor-3-A102G |
| pETDuet-muta-A102H | pETDuet-1 contained the gene of Glusor-3-A102H |
| pETDuet-muta-A102I | pETDuet-1 contained the gene of Glusor-3-A102I |
| pETDuet-muta-A102K | pETDuet-1 contained the gene of Glusor-3-A102K |
| pETDuet-muta-A102L | pETDuet-1 contained the gene of Glusor-3-A102L |
| pETDuet-muta-A102M | pETDuet-1 contained the gene of Glusor-3-A102M |
| pETDuet-muta-A102N | pETDuet-1 contained the gene of Glusor-3-A102N |
| pETDuet-muta-A102P | pETDuet-1 contained the gene of Glusor-3-A102P |
| pETDuet-muta-A102Q | pETDuet-1 contained the gene of Glusor-3-A102Q |
| pETDuet-muta-A102R | pETDuet-1 contained the gene of Glusor-3-A102R |
| pETDuet-muta-A102S | pETDuet-1 contained the gene of Glusor-3-A102S |
| pETDuet-muta-A102T | pETDuet-1 contained the gene of Glusor-3-A102T |
| pETDuet-muta-A102V | pETDuet-1 contained the gene of Glusor-3-A102V |
| pETDuet-muta-A102W | pETDuet-1 contained the gene of Glusor-3-A102W |
| pETDuet-muta-A102Y | pETDuet-1 contained the gene of Glusor-3-A102Y |
| pETDuet-muta-G103A | pETDuet-1 contained the gene of Glusor-3-G103A |
| pETDuet-muta-G103C | pETDuet-1 contained the gene of Glusor-3-G103C |
| pETDuet-muta-G103D | pETDuet-1 contained the gene of Glusor-3-G103D |
| pETDuet-muta-G103E | pETDuet-1 contained the gene of Glusor-3-G103E |
| pETDuet-muta-G103F | pETDuet-1 contained the gene of Glusor-3-G103F |
| pETDuet-muta-G103H | pETDuet-1 contained the gene of Glusor-3-G103H |
| pETDuet-muta-G103I | pETDuet-1 contained the gene of Glusor-3-G103I |
| pETDuet-muta-G103K | pETDuet-1 contained the gene of Glusor-3-G103K |
| pETDuet-muta-G103L | pETDuet-1 contained the gene of Glusor-3-G103L |
| pETDuet-muta-G103M | pETDuet-1 contained the gene of Glusor-3-G103M |
| pETDuet-muta-G103N | pETDuet-1 contained the gene of Glusor-3-G103N |
| pETDuet-muta-G103P | pETDuet-1 contained the gene of Glusor-3-G103P |
| pETDuet-muta-G103Q | pETDuet-1 contained the gene of Glusor-3-G103Q |
| pETDuet-muta-G103R | pETDuet-1 contained the gene of Glusor-3-G103R |
| pETDuet-muta-G103S | pETDuet-1 contained the gene of Glusor-3-G103S |
| pETDuet-muta-G103T | pETDuet-1 contained the gene of Glusor-3-G103T |
| pETDuet-muta-G103V | pETDuet-1 contained the gene of Glusor-3-G103V |
| pETDuet-muta-G103W | pETDuet-1 contained the gene of Glusor-3-G103W |
| pETDuet-muta-G103Y | pETDuet-1 contained the gene of Glusor-3-G103Y |
| pETDuet-muta-G350A | pETDuet-1 contained the gene of Glusor-3-G350A |
| pETDuet-muta-G350C | pETDuet-1 contained the gene of Glusor-3-G350C |
| pETDuet-muta-G350D | pETDuet-1 contained the gene of Glusor-3-G350D |
| pETDuet-muta-G350E | pETDuet-1 contained the gene of Glusor-3-G350E |
| pETDuet-muta-G350F | pETDuet-1 contained the gene of Glusor-3-G350F |
| pETDuet-muta-G350H | pETDuet-1 contained the gene of Glusor-3-G350H |
| pETDuet-muta-G350I | pETDuet-1 contained the gene of Glusor-3-G350I |
| pETDuet-muta-G350K | pETDuet-1 contained the gene of Glusor-3-G350K |
| pETDuet-muta-G350L | pETDuet-1 contained the gene of Glusor-3-G350L |
| pETDuet-muta-G350M | pETDuet-1 contained the gene of Glusor-3-G350M |
| pETDuet-muta-G350N | pETDuet-1 contained the gene of Glusor-3-G350N |
| pETDuet-muta-G350P | pETDuet-1 contained the gene of Glusor-3-G350P |
| pETDuet-muta-G350Q | pETDuet-1 contained the gene of Glusor-3-G350Q |
| pETDuet-muta-G350R | pETDuet-1 contained the gene of Glusor-3-G350R |
| pETDuet-muta-G350S | pETDuet-1 contained the gene of Glusor-3-G350S |
| pETDuet-muta-G350T | pETDuet-1 contained the gene of Glusor-3-G350T |
| pETDuet-muta-G350V | pETDuet-1 contained the gene of Glusor-3-G350V |
| pETDuet-muta-G350W | pETDuet-1 contained the gene of Glusor-3-G350W |
| pETDuet-muta-G350Y | pETDuet-1 contained the gene of Glusor-3-G350Y |
| pETDuet-muta-G351A | pETDuet-1 contained the gene of Glusor-3-G351A |
| pETDuet-muta-G351C | pETDuet-1 contained the gene of Glusor-3-G351C |
| pETDuet-muta-G351D | pETDuet-1 contained the gene of Glusor-3-G351D |
| pETDuet-muta-G351E | pETDuet-1 contained the gene of Glusor-3-G351E |
| pETDuet-muta-G351F | pETDuet-1 contained the gene of Glusor-3-G351F |
| pETDuet-muta-G351H | pETDuet-1 contained the gene of Glusor-3-G351H |
| pETDuet-muta-G351I | pETDuet-1 contained the gene of Glusor-3-G351I |
| pETDuet-muta-G351K | pETDuet-1 contained the gene of Glusor-3-G351K |
| pETDuet-muta-G351L | pETDuet-1 contained the gene of Glusor-3-G351L |
| pETDuet-muta-G351M | pETDuet-1 contained the gene of Glusor-3-G351M |
| pETDuet-muta-G351N | pETDuet-1 contained the gene of Glusor-3-G351N |
| pETDuet-muta-G351P | pETDuet-1 contained the gene of Glusor-3-G351P |
| pETDuet-muta-G351Q | pETDuet-1 contained the gene of Glusor-3-G351Q |
| pETDuet-muta-G351R | pETDuet-1 contained the gene of Glusor-3-G351R |
| pETDuet-muta-G351S | pETDuet-1 contained the gene of Glusor-3-G351S |
| pETDuet-muta-G351T | pETDuet-1 contained the gene of Glusor-3-G351T |
| pETDuet-muta-G351V | pETDuet-1 contained the gene of Glusor-3-G351V |
| pETDuet-muta-G351W | pETDuet-1 contained the gene of Glusor-3-G351W |
| pETDuet-muta-G351Y | pETDuet-1 contained the gene of Glusor-3-G351Y |
| pETDuet-muta-C352A | pETDuet-1 contained the gene of Glusor-3-C352A |
| pETDuet-muta-C352D | pETDuet-1 contained the gene of Glusor-3-C352D |
| pETDuet-muta-C352E | pETDuet-1 contained the gene of Glusor-3-C352E |
| pETDuet-muta-C352F | pETDuet-1 contained the gene of Glusor-3-C352F |
| pETDuet-muta-C352G | pETDuet-1 contained the gene of Glusor-3-C352G |
| pETDuet-muta-C352H | pETDuet-1 contained the gene of Glusor-3-C352H |
| pETDuet-muta-C352I | pETDuet-1 contained the gene of Glusor-3-C352I |
| pETDuet-muta-C352K | pETDuet-1 contained the gene of Glusor-3-C352K |
| pETDuet-muta-C352L | pETDuet-1 contained the gene of Glusor-3-C352L |
| pETDuet-muta-C352M | pETDuet-1 contained the gene of Glusor-3-C352M |
| pETDuet-muta-C352N | pETDuet-1 contained the gene of Glusor-3-C352N |
| pETDuet-muta-C352P | pETDuet-1 contained the gene of Glusor-3-C352P |
| pETDuet-muta-C352Q | pETDuet-1 contained the gene of Glusor-3-C352Q |
| pETDuet-muta-C352R | pETDuet-1 contained the gene of Glusor-3-C352R |
| pETDuet-muta-C352S | pETDuet-1 contained the gene of Glusor-3-C352S |
| pETDuet-muta-C352T | pETDuet-1 contained the gene of Glusor-3-C352T |
| pETDuet-muta-C352V | pETDuet-1 contained the gene of Glusor-3-C352V |
| pETDuet-muta-C352W | pETDuet-1 contained the gene of Glusor-3-C352W |
| pETDuet-muta-C352Y | pETDuet-1 contained the gene of Glusor-3-C352Y |
| pETDuet-muta-S101A, G350K | pETDuet-1 contained the gene of Glusor-3-S101A, G350K |
| pETDuet-muta-S101A, G350N (Glusor) | pETDuet-1 contained the gene of Glusor-3-S101A, G350N (Glusor) |
| pETDuet-muta-S101A, G350Q | pETDuet-1 contained the gene of Glusor-3-S101A, G350Q |
| pETDuet-muta-S101A, G350R | pETDuet-1 contained the gene of Glusor-3-S101A, G350R |
| pETDuet-muta-S101A, G351A | pETDuet-1 contained the gene of Glusor-3-S101A, G351A |
| pETDuet-muta-S101A, G351C | pETDuet-1 contained the gene of Glusor-3-S101A, G351C |
| pETDuet-muta-S101A, C352A | pETDuet-1 contained the gene of Glusor-3-S101A, C352A |
| pETDuet-muta-S101A, C352E | pETDuet-1 contained the gene of Glusor-3-S101A, C352E |
| pETDuet-muta-S101A, C352I | pETDuet-1 contained the gene of Glusor-3-S101A, C352I |
| pETDuet-muta-S101A, C352N | pETDuet-1 contained the gene of Glusor-3-S101A, C352N |
| pETDuet-muta-S101A, C352P | pETDuet-1 contained the gene of Glusor-3-S101A, C352P |
| pETDuet-muta-S101A, C352S | pETDuet-1 contained the gene of Glusor-3-S101A, C352S |
| pETDuet-muta-S101A, C352V | pETDuet-1 contained the gene of Glusor-3-S101A, C352V |
| pETDuet-muta-S101A, C352Y | pETDuet-1 contained the gene of Glusor-3-S101A, C352Y |
| pETDuet-muta-S101G, G350K | pETDuet-1 contained the gene of Glusor-3-S101G, G350K |
| pETDuet-muta-S101G, G350N | pETDuet-1 contained the gene of Glusor-3-S101G, G350N |
| pETDuet-muta-S101G, G350Q | pETDuet-1 contained the gene of Glusor-3-S101G, G350Q |
| pETDuet-muta-S101G, G350R | pETDuet-1 contained the gene of Glusor-3-S101G, G350R |
| pETDuet-muta-S101G, G351A | pETDuet-1 contained the gene of Glusor-3-S101G, G351A |
| pETDuet-muta-S101G, G351C | pETDuet-1 contained the gene of Glusor-3-S101G, G351C |
| pETDuet-muta-S101G, C352A | pETDuet-1 contained the gene of Glusor-3-S101G, C352A |
| pETDuet-muta-S101G, C352E | pETDuet-1 contained the gene of Glusor-3-S101G, C352E |
| pETDuet-muta-S101G, C352I | pETDuet-1 contained the gene of Glusor-3-S101G, C352I |
| pETDuet-muta-S101G, C352N | pETDuet-1 contained the gene of Glusor-3-S101G, C352N |
| pETDuet-muta-S101G, C352P | pETDuet-1 contained the gene of Glusor-3-S101G, C352P |
| pETDuet-muta-S101G, C352S | pETDuet-1 contained the gene of Glusor-3-S101G, C352S |
| pETDuet-muta-S101G, C352V | pETDuet-1 contained the gene of Glusor-3-S101G, C352V |
| pETDuet-muta-S101G, C352Y | pETDuet-1 contained the gene of Glusor-3-S101G, C352Y |
| pETDuet-muta-A102D, G350K | pETDuet-1 contained the gene of Glusor-3-A102D, G350K |
| pETDuet-muta-A102D, G350N | pETDuet-1 contained the gene of Glusor-3-A102D, G350N |
| pETDuet-muta-A102D, G350Q | pETDuet-1 contained the gene of Glusor-3-A102D, G350Q |
| pETDuet-muta-A102D, G350R | pETDuet-1 contained the gene of Glusor-3-A102D, G350R |
| pETDuet-muta-A102D, G351A | pETDuet-1 contained the gene of Glusor-3-A102D, G351A |
| pETDuet-muta-A102D, G351C | pETDuet-1 contained the gene of Glusor-3-A102D, G351C |
| pETDuet-muta-A102D, C352A | pETDuet-1 contained the gene of Glusor-3-A102D, C352A |
| pETDuet-muta-A102D, C352E | pETDuet-1 contained the gene of Glusor-3-A102D, C352E |
| pETDuet-muta-A102D, C352I | pETDuet-1 contained the gene of Glusor-3-A102D, C352I |
| pETDuet-muta-A102D, C352N | pETDuet-1 contained the gene of Glusor-3-A102D, C352N |
| pETDuet-muta-A102D, C352P | pETDuet-1 contained the gene of Glusor-3-A102D, C352P |
| pETDuet-muta-A102D, C352S | pETDuet-1 contained the gene of Glusor-3-A102D, C352S |
| pETDuet-muta-A102D, C352V | pETDuet-1 contained the gene of Glusor-3-A102D, C352V |
| pETDuet-muta-A102D, C352Y | pETDuet-1 contained the gene of Glusor-3-A102D, C352Y |
| pETDuet-muta-A102I, G350K | pETDuet-1 contained the gene of Glusor-3-A102I, G350K |
| pETDuet-muta-A102I, G350N | pETDuet-1 contained the gene of Glusor-3-A102I, G350N |
| pETDuet-muta-A102I, G350Q | pETDuet-1 contained the gene of Glusor-3-A102I, G350Q |
| pETDuet-muta-A102I, G350R | pETDuet-1 contained the gene of Glusor-3-A102I, G350R |
| pETDuet-muta-A102I, G351A | pETDuet-1 contained the gene of Glusor-3-A102I, G351A |
| pETDuet-muta-A102I, G351C | pETDuet-1 contained the gene of Glusor-3-A102I, G351C |
| pETDuet-muta-A102I, C352A | pETDuet-1 contained the gene of Glusor-3-A102I, C352A |
| pETDuet-muta-A102I, C352E | pETDuet-1 contained the gene of Glusor-3-A102I, C352E |
| pETDuet-muta-A102I, C352I | pETDuet-1 contained the gene of Glusor-3-A102I, C352I |
| pETDuet-Glusor | pETDuet-1 contained the gene of Glusor |
| pETDuet-cpSFYFP | pETDuet-1 contained the gene of cpSFYFP |
| pETDuet-Glusor^Y84A^ | pETDuet-1 contained the gene of Glusor with Y84A in CsiR-LBD region |
| pETDuet-Glusor^R87A^ | pETDuet-1 contained the gene of Glusor with R87A in CsiR-LBD region |
| pETDuet-Glusor^H137A^ | pETDuet-1 contained the gene of Glusor with H137A in CsiR-LBD region |
| pETDuet-Glusor^H141A^ | pETDuet-1 contained the gene of Glusor with H141A in CsiR-LBD region |
| pETDuet-Glusor^R168A^ | pETDuet-1 contained the gene of Glusor with R168A in CsiR-LBD region |
| pETDuet-Glusor^H189A^ | pETDuet-1 contained the gene of Glusor with H189A in CsiR-LBD region |
| pETDuet-Glusor^H211A^ | pETDuet-1 contained the gene of Glusor with H211A in CsiR-LBD region |
| pEcCas | Vector for Cas9 expression in bacteria; Km^r^ |
| pEcgRNA | Vector for sgRNA expression in bacteria; Spe^r^ |
| pEcgRNA-N20-*csiD* | pEcgRNA contained sgRNA sequence target for gene of *csiD* |
| pEcgRNA-N20-*kgtP* | pEcgRNA contained sgRNA sequence target for gene of *kgtP* |
| pEcgRNA-N20-*dctA* | pEcgRNA contained sgRNA sequence target for gene of *dctA* |
| pEcgRNA-N20-*dauA* | pEcgRNA contained sgRNA sequence target for gene of *dauA* |
| pEcgRNA-N20-*dcuA* | pEcgRNA contained sgRNA sequence target for gene of *dcuA* |
| pEcgRNA-N20-*ynfM* | pEcgRNA contained sgRNA sequence target for gene of *ynfM* |
| pcDNA3.1^(+)^ | Vector for protein expression in mammalian cells; Ap^r^ |
| pcDNA3.1^(+)^-Glusor | pcDNA3.1^(+)^ contained Kozak sequence and the gene of Glusor |
| pcDNA3.1^(+)^-Glusor-Mito | pcDNA3.1^(+)^ contained Kozak sequence, mitochondrial targeting sequence, and the gene of Glusor |
| pcDNA3.1^(+)^-Glusor-Cyto | pcDNA3.1^(+)^ contained Kozak sequence, nuclear-excluded targeting sequence, and the gene of Glusor |
| pcDNA3.1^(+)^-Glusor-Nuc | pcDNA3.1^(+)^ contained Kozak sequence, the gene of Glusor, and nuclear targeting sequence |
| pcDNA3.1^(+)^-cpSFYFP | pcDNA3.1^(+)^ contained Kozak sequence and the gene of cpSFYFP |
| pcDNA3.1^(+)^-cpSFYFP-Mito | pcDNA3.1^(+)^ contained Kozak sequence, mitochondrial targeting sequence, and the gene of cpSFYFP |
| pcDNA3.1^(+)^-cpSFYFP-Cyto | pcDNA3.1^(+)^ contained Kozak sequence, nuclear-excluded targeting sequence, and the gene of cpSFYFP |
| pcDNA3.1^(+)^-cpSFYFP-Nuc | pcDNA3.1^(+)^ contained Kozak sequence, the gene of cpSFYFP, and nuclear targeting sequence |
| pcDNA3.1^(+)^-SLC22A6 | pcDNA3.1^(+)^ contained Kozak sequence, the gene of SLC22A6 |

aTetr, tetracycline resistant; Sper, spectinomycin resistant; Apr, ampicillin resistant; Kmr, kanamycin resistant.

#### Table S4 Oligonucleotides used in this study.

| **Primer** | **Sequence (5’-3’)** | **Use** |
| --- | --- | --- |
| **Construction of CsiR, CsiR-LBD, and mutations of CsiR-LBD** | | |
| CsiR-F1 | GCATCGGTGCCCGCTAGAAGCTTGCGGCCGCATAATGCT | Amplification of the downstream fragment of CsiR in pETDuet-1 (forward) |
| CsiR-R1 | TCGGGGGGCGAGCGCTTCCATCGGATCCTGGCTGTGGTG | Amplification of the upstream fragment of CsiR in pETDuet-1 (reverse) |
| CsiR-F2 | CACCACAGCCAGGATCCGATGGAAGCGCTCGCCCCCCGA | Amplification of CsiR (forward) |
| CsiR-R2 | ATTATGCGGCCGCAAGCTTCTAGCGGGCACCGATGCCTT | Amplification of CsiR (reverse) |
| CsiR-LBD-F1 | AGGCATCGGTGCCCGCTAGAAGCTTGCGGCCGCATAATG | Amplification of the downstream fragment of CsiR-LBD in pETDuet-1 (forward) |
| CsiR-LBD-R1 | TCATCTCATCCAGCGGATCCTGGCTGTGGTGATGAT | Amplification of the upstream fragment of CsiR-LBD in pETDuet-1 (reverse) |
| CsiR-LBD-F2 | CACCACAGCCAGGATCCGCTGGATGAGATGAACGACATC | Amplification of CsiR-LBD (forward) |
| CsiR-LBD-R2 | ATTATGCGGCCGCAAGCTTCTAGCGGGCACCGATGCCTT | Amplification of CsiR-LBD (reverse) |
| Duet-middle-F | GCAGCGAGTCAGTGAGCGAGGAAGCGGAAG | Amplification of the downstream fragment of plasmid backbone in pETDuet-1 (forward) |
| Duet-middle-R | CTTCCGCTTCCTCGCTCACTGACTCGCTGC | Amplification of the upstream fragment of plasmid backbone in pETDuet-1 (reverse) |
| Y84A-F | GATGAACGACATCgccGACGCCCGCGCCAACCTGGAAGC | Amplification of CsiR-LBD-Y84A (forward) |
| Y84A-R | TGGCGCGGGCGTCggcGATGTCGTTCATCTCATCCAGCG | Amplification of CsiR-LBD-Y84A (reverse) |
| R87A-F | CATCTACGACGCCgcaGCCAACCTGGAAGCGATGATCAT | Amplification of CsiR-LBD-R87A (forward) |
| R87A-R | TCCAGGTTGGCtgcGGCGTCGTAGATGTCGTTCATCTCA | Amplification of CsiR-LBD-R87A (reverse) |
| H137A-F | GTGTGGGACGAGCGGgccAAGGCGTTCCATACTGCCATT | Amplification of CsiR-LBD-H137A (forward) |
| H137A-R | TATGGAACGCCTTggcCCGCTCGTCCCACACATCCAGCC | Amplification of CsiR-LBD-H137A (reverse) |
| H141A-F | GCACAAGGCGTTCgccACTGCCATTGCCTCGGGCTGCGG | Amplification of CsiR-LBD-H141A (forward) |
| H141A-R | AGGCAATGGCAGTggcGAACGCCTTGTGCCGCTCGTCCC | Amplification of CsiR-LBD-H141A (reverse) |
| R168A-F | GGCCGAGCGCTACgccCACCTGTGGCTGACGCAGACGGT | Amplification of CsiR-LBD-R168A (forward) |
| R168A-R | CGTCAGCCACAGGTGggcGTAGCGCTCGGCCTGGTCGAA | Amplification of CsiR-LBD-R168A (reverse) |
| H189A-F | TCAAGCGCCAGGAGgcaGCGGCACTGGTCGAGGTGATCC | Amplification of CsiR-LBD-H189A (forward) |
| H189A-R | GACCAGTGCCGCtgcCTCCTGGCGCTTGAGCGCCAGCGC | Amplification of CsiR-LBD-H189A (reverse) |
| H211A-F | TGATGCGCTCGgccCTGATGACACCGGTACCGATCATTG | Amplification of CsiR-LBD-H211A (forward) |
| H211A-R | TACCGGTGTCATCAGggcCGAGCGCATCATGGCGCTGGC | Amplification of CsiR-LBD-H211A (reverse) |
| **Construction of Glusor-1 and Glusor-2 (Screening of insertion sites for cpSFYFP)** | | |
| cpSFYFP-F | AGCGCAGGCTTCaacagcgacaacgtctat | Amplification of cpSFYFP (forward) |
| cpSFYFP-R | ACAGCCACCgttgtactccagcttgtgccc | Amplification of cpSFYFP (reverse) |
| SF-0-231R-F | gtacaacGGTGGCTGTTAGAAGCTTGCGGCCGCATAATG | Amplification of the downstream fragment of CsiR (forward) |
| SF-0-231R-R | gctgttGAAGCCTGCGCTGCGGGCACCGATGCCTTC | Amplification of the upstream fragment and amino acids 1 to 231 of CsiR (reverse) |
| SF-1-226E/227G-F | gctggagtacaacGGTGGCTGTGGCATCGGTGCCCG | Amplification of the downstream fragment and amino acids 227 to 231 of CsiR (forward) |
| SF-1-226E/227G-R | tcgctgttGAAGCCTGCGCTTTCGGCGTGCATGATCT | Amplification of the upstream fragment and amino acids 1 to 226 of CsiR (reverse) |
| SF-2-225A/226E-F | tggagtacaacGGTGGCTGTGAAGGCATCGGTGCCC | Amplification of the downstream fragment and amino acids 226 to 231 of CsiR (forward) |
| SF-2-225A/226E-R | tcgctgttGAAGCCTGCGCTGGCGTGCATGATCTGCG | Amplification of the upstream fragment and amino acids 1 to 225 of CsiR (reverse) |
| SF-3-215P/216V-F | tacaacGGTGGCTGTGTACCGATCATTGCG | Amplification of the downstream fragment and amino acids 216 to 231 of CsiR (forward) |
| SF-3-215P/216V-R | tcgctgttGAAGCCTGCGCTCGGTGTCATCAGGTGCG | Amplification of the upstream fragment and amino acids 1 to 215 of CsiR (reverse) |
| SF-4-214T/215P-F | tacaacGGTGGCTGTCCGGTACCGATCATT | Amplification of the downstream fragment and amino acids 215 to 231 of CsiR (forward) |
| SF-4-214T/215P-R | tcgctgttGAAGCCTGCGCTTGTCATCAGGTGCGAGC | Amplification of the upstream fragment and amino acids 1 to 214 of CsiR (reverse) |
| SF-5-213M/214T-F | tacaacGGTGGCTGTACACCGGTACCGATC | Amplification of the downstream fragment and amino acids 214 to 231 of CsiR (forward) |
| SF-5-213M/214T-R | cgctgttGAAGCCTGCGCTCATCAGGTGCGAGCG | Amplification of the upstream fragment and amino acids 1 to 213 of CsiR (reverse) |
| SF-6-212L/213M-F | agtacaacGGTGGCTGTATGACACCGGTACCGATC | Amplification of the downstream fragment and amino acids 213 to 231 of CsiR (forward) |
| SF-6-212L/213M-R | cgctgttGAAGCCTGCGCTCAGGTGCGAGCGCAT | Amplification of the upstream fragment and amino acids 1 to 212 of CsiR (reverse) |
| SF-7-211H/212L-F | gagtacaacGGTGGCTGTCTGATGACACCGGTACC | Amplification of the downstream fragment and amino acids 212 to 231 of CsiR (forward) |
| SF-7-211H/212L-R | gctgttGAAGCCTGCGCTGTGCGAGCGCATCATG | Amplification of the upstream fragment and amino acids 1 to 211 of CsiR (reverse) |
| SF-8-210S/211H-F | gagtacaacGGTGGCTGTCACCTGATGACACCGGT | Amplification of the downstream fragment and amino acids 211 to 231 of CsiR (forward) |
| SF-8-210S/211H-R | ctgttGAAGCCTGCGCTCGAGCGCATCATGGC | Amplification of the upstream fragment and amino acids 1 to 210 of CsiR (reverse) |
| SF-9-200D/201A-F | gagtacaacGGTGGCTGTGCCAAAACCGCCAGCG | Amplification of the downstream fragment and amino acids 201 to 231 of CsiR (forward) |
| SF-9-200D/201A-R | ctgttGAAGCCTGCGCTATCCCGGGCGAGGAT | Amplification of the upstream fragment and amino acids 1 to 200 of CsiR (reverse) |
| SF-10-199R/200D-F | gagtacaacGGTGGCTGTGATGCCAAAACCGCCA | Amplification of the downstream fragment and amino acids 200 to 231 of CsiR (forward) |
| SF-10-199R/200D-R | ctgttGAAGCCTGCGCTCCGGGCGAGGATCACC | Amplification of the upstream fragment and amino acids 1 to 199 of CsiR (reverse) |
| SF-11-198A/199R-F | gagtacaacGGTGGCTGTCGGGATGCCAAAACCG | Amplification of the downstream fragment and amino acids 199 to 231 of CsiR (forward) |
| SF-11-198A/199R-R | gctgttGAAGCCTGCGCTGGCGAGGATCACCTCGA | Amplification of the upstream fragment and amino acids 1 to 198 of CsiR (reverse) |
| SF-12-197L/198A-F | gagtacaacGGTGGCTGTGCCCGGGATGCCAAAA | Amplification of the downstream fragment and amino acids 198 to 231 of CsiR (forward) |
| SF-12-197L/198A-R | gctgttGAAGCCTGCGCTGAGGATCACCTCGACCA | Amplification of the upstream fragment and amino acids 1 to 197 of CsiR (reverse) |
| SF-13-178S/179E-F | gagtacaacGGTGGCTGTGAAGAGGCGCTGGCGCTC | Amplification of the downstream fragment and amino acids 179 to 231 of CsiR (forward) |
| SF-13-178S/179E-R | ctgttGAAGCCTGCGCTGGAAAACACCGTCTGCG | Amplification of the upstream fragment and amino acids 1 to 178 of CsiR (reverse) |
| SF-14-177F/178S-F | gagtacaacGGTGGCTGTTCCGAAGAGGCGCTGGCGCT | Amplification of the downstream fragment and amino acids 178 to 231 of CsiR (forward) |
| SF-14-177F/178S-R | ctgttGAAGCCTGCGCTAAACACCGTCTGCGTCA | Amplification of the upstream fragment and amino acids 1 to 177 of CsiR (reverse) |
| SF-15-176V/177F-F | tggagtacaacGGTGGCTGTTTTTCCGAAGAGGCGC | Amplification of the downstream fragment and amino acids 177 to 231 of CsiR (forward) |
| SF-15-176V/177F-R | tcgctgttGAAGCCTGCGCTCACCGTCTGCGTCA | Amplification of the upstream fragment and amino acids 1 to 176 of CsiR (reverse) |
| SF-16-175T/176V-F | tggagtacaacGGTGGCTGTGTGTTTTCCGAAGAGG | Amplification of the downstream fragment and amino acids 176 to 231 of CsiR (forward) |
| SF-16-175T/176V-R | tcgctgttGAAGCCTGCGCTCGTCTGCGTCAGCCAC | Amplification of the upstream fragment and amino acids 1 to 175 of CsiR (reverse) |
| SF-17-174Q/175T-F | tggagtacaacGGTGGCTGTACGGTGTTTTCCGAAG | Amplification of the downstream fragment and amino acids 175 to 231 of CsiR (forward) |
| SF-17-174Q/175T-R | tcgctgttGAAGCCTGCGCTCTGCGTCAGCCACAGGT | Amplification of the upstream fragment and amino acids 1 to 174 of CsiR (reverse) |
| SF-18-173T/174Q-F | tggagtacaacGGTGGCTGTCAGACGGTGTTTTCCGA | Amplification of the downstream fragment and amino acids 174 to 231 of CsiR (forward) |
| SF-18-173T/174Q-R | tcgctgttGAAGCCTGCGCTCGTCAGCCACAGGTGG | Amplification of the upstream fragment and amino acids 1 to 173 of CsiR (reverse) |
| SF-19-150S/151K-F | tggagtacaacGGTGGCTGTAAGCACCTGCTGCAGGC | Amplification of the downstream fragment and amino acids 151 to 231 of CsiR (forward) |
| SF-19-150S/151K-R | tcgctgttGAAGCCTGCGCTGGAGCCGCAGCCCGAG | Amplification of the upstream fragment and amino acids 1 to 150 of CsiR (reverse) |
| SF-20-149G/150S-F | tggagtacaacGGTGGCTGTTCCAAGCACCTGCTGCA | Amplification of the downstream fragment and amino acids 150 to 231 of CsiR (forward) |
| SF-20-149G/150S-R | tcgctgttGAAGCCTGCGCTGCCGCAGCCCGAGGCAA | Amplification of the upstream fragment and amino acids 1 to 149 of CsiR (reverse) |
| SF-21-148C/149G-F | tggagtacaacGGTGGCTGTGGCTCCAAGCACCTGCT | Amplification of the downstream fragment and amino acids 149 to 231 of CsiR (forward) |
| SF-21-148C/149G-R | tcgctgttGAAGCCTGCGCTGCAGCCCGAGGCAATG | Amplification of the upstream fragment and amino acids 1 to 148 of CsiR (reverse) |
| SF-22-147G/148C-F | tggagtacaacGGTGGCTGTTGCGGCTCCAAGCACCT | Amplification of the downstream fragment and amino acids 148 to 231 of CsiR (forward) |
| SF-22-147G/148C-R | tcgctgttGAAGCCTGCGCTGCCCGAGGCAATGGCAGT | Amplification of the upstream fragment and amino acids 1 to 147 of CsiR (reverse) |
| SF-23-146S/147G-F | tggagtacaacGGTGGCTGTGGCTGCGGCTCCAAGCAC | Amplification of the downstream fragment and amino acids 147 to 231 of CsiR (forward) |
| SF-23-146S/147G-R | tcgctgttGAAGCCTGCGCTCGAGGCAATGGCAGTA | Amplification of the upstream fragment and amino acids 1 to 146 of CsiR (reverse) |
| SF-24-145A/146S-F | tggagtacaacGGTGGCTGTTCGGGCTGCGGCTCCAAG | Amplification of the downstream fragment and amino acids 146 to 231 of CsiR (forward) |
| SF-24-145A/146S-R | tcgctgttGAAGCCTGCGCTGGCAATGGCAGTATGG | Amplification of the upstream fragment and amino acids 1 to 145 of CsiR (reverse) |
| SF-25-129R/130L-F | tggagtacaacGGTGGCTGTCTGGATGTGTGGGACGAG | Amplification of the downstream fragment and amino acids 130 to 231 of CsiR (forward) |
| SF-25-129R/130L-R | tcgctgttGAAGCCTGCGCTCCGCTGCTCGCGGGTT | Amplification of the upstream fragment and amino acids 1 to 129 of CsiR (reverse) |
| SF-26-128Q/129R-F | tggagtacaacGGTGGCTGTCGGCTGGATGTGTGGGACG | Amplification of the downstream fragment and amino acids 129 to 231 of CsiR (forward) |
| SF-26-128Q/129R-R | tcgctgttGAAGCCTGCGCTCTGCTCGCGGGTTTTTAC | Amplification of the upstream fragment and amino acids 1 to 128 of CsiR (reverse) |
| SF-27-127E/128Q-F | ggagtacaacGGTGGCTGTCAGCGGCTGGATGTGTGGG | Amplification of the downstream fragment and amino acids 128 to 231 of CsiR (forward) |
| SF-27-127E/128Q-R | cgctgttGAAGCCTGCGCTCTCGCGGGTTTTTACTTCC | Amplification of the upstream fragment and amino acids 1 to 127 of CsiR (reverse) |
| SF-28-126R/127E-F | GAGTACAACGGTGGCTGTGAGCAGCGGCTGGATGTGTG | Amplification of the downstream fragment and amino acids 127 to 231 of CsiR (forward) |
| SF-28-126R/127E-R | gctgttGAAGCCTGCGCTGCGGGTTTTTACTTCCACCA | Amplification of the upstream fragment and amino acids 1 to 126 of CsiR (reverse) |
| SF-29-125T/126R-F | gagtacaacGGTGGCTGTCGCGAGCAGCGGCTGGATGT | Amplification of the downstream fragment and amino acids 126 to 231 of CsiR (forward) |
| SF-29-125T/126R-R | tcgctgttGAAGCCTGCGCTGGTTTTTACTTCCACCAC | Amplification of the upstream fragment and amino acids 1 to 125 of CsiR (reverse) |
| SF-30-124K/125T-F | gagtacaacGGTGGCTGTACCCGCGAGCAGCGGCTGG | Amplification of the downstream fragment and amino acids 125 to 231 of CsiR (forward) |
| SF-30-124K/125T-R | tcgctgttGAAGCCTGCGCTTTTTACTTCCACCACCTT | Amplification of the upstream fragment and amino acids 1 to 124 of CsiR (reverse) |
| SF-31-123V/124K-F | gagtacaacGGTGGCTGTAAAACCCGCGAGCAGCGGC | Amplification of the downstream fragment and amino acids 124 to 231 of CsiR (forward) |
| SF-31-123V/124K-R | tcgctgttGAAGCCTGCGCTTACTTCCACCACCTTGGC | Amplification of the upstream fragment and amino acids 1 to 123 of CsiR (reverse) |
| SF-32-122E/123V-F | gagtacaacGGTGGCTGTGTAAAAACCCGCGAGCAG | Amplification of the downstream fragment and amino acids 123 to 231 of CsiR (forward) |
| SF-32-122E/123V-R | tgttGAAGCCTGCGCTTTCCACCACCTTGGCC | Amplification of the upstream fragment and amino acids 1 to 122 of CsiR (reverse) |
| SF-33-121V/122E-F | agtacaacGGTGGCTGTGAAGTAAAAACCCGCGA | Amplification of the downstream fragment and amino acids 122 to 231 of CsiR (forward) |
| SF-33-121V/122E-R | tgttGAAGCCTGCGCTCACCACCTTGGCCAAT | Amplification of the upstream fragment and amino acids 1 to 121 of CsiR (reverse) |
| SF-34-120V/121V-F | agtacaacGGTGGCTGTGTGGAAGTAAAAACCCGC | Amplification of the downstream fragment and amino acids 121 to 231 of CsiR (forward) |
| SF-34-120V/121V-R | ctgttGAAGCCTGCGCTCACCTTGGCCAATGTAT | Amplification of the upstream fragment and amino acids 1 to 120 of CsiR (reverse) |
| SF-35-119K/120V-F | agtacaacGGTGGCTGTGTGGTGGAAGTAAAAAC | Amplification of the downstream fragment and amino acids 120 to 231 of CsiR (forward) |
| SF-35-119K/120V-R | ctgttGAAGCCTGCGCTCTTGGCCAATGTATGCGA | Amplification of the upstream fragment and amino acids 1 to 119 of CsiR (reverse) |
| SF-36-118A/119K-F | agtacaacGGTGGCTGTAAGGTGGTGGAAGTAAA | Amplification of the downstream fragment and amino acids 119 to 231 of CsiR (forward) |
| SF-36-118A/119K-R | ctgttGAAGCCTGCGCTGGCCAATGTATGCGAGT | Amplification of the upstream fragment and amino acids 1 to 118 of CsiR (reverse) |
| SF-37-113H/114S-F | agtacaacGGTGGCTGTTCGCATACATTGGCCAA | Amplification of the downstream fragment and amino acids 114 to 231 of CsiR (forward) |
| SF-37-113H/114S-R | ctgttGAAGCCTGCGCTGTGGGCCAGCACCGATG | Amplification of the upstream fragment and amino acids 1 to 113 of CsiR (reverse) |
| SF-38-112A/113H-F | GGCATCGGTGCTGGCCAGCGCAGGCTTCaaca | Amplification of the downstream fragment and amino acids 113 to 231 of CsiR (forward) |
| SF-38-112A/113H-R | tgttGAAGCCTGCGCTGGCCAGCACCGATGCCTCCCAGG | Amplification of the upstream fragment and amino acids 1 to 112 of CsiR (reverse) |
| SF-39-104D/105A-F | agtacaacGGTGGCTGTGCCTGGGAGGCATCGGTGCT | Amplification of the downstream fragment and amino acids 105 to 231 of CsiR (forward) |
| SF-39-104D/105A-R | ctgttGAAGCCTGCGCTGTCATCGCCGCGCTCGA | Amplification of the upstream fragment and amino acids 1 to 104 of CsiR (reverse) |
| SF-40-103D/104D-F | agtacaacGGTGGCTGTGACGCCTGGGAGGCATCG | Amplification of the downstream fragment and amino acids 104 to 231 of CsiR (forward) |
| SF-40-103D/104D-R | ctgttGAAGCCTGCGCTATCGCCGCGCTCGATGG | Amplification of the upstream fragment and amino acids 1 to 103 of CsiR (reverse) |
| SF-41-102G/103D-F | agtacaacGGTGGCTGTGATGACGCCTGGGAGGCA | Amplification of the downstream fragment and amino acids 103 to 231 of CsiR (forward) |
| SF-41-102G/103D-R | ctgttGAAGCCTGCGCTGCCGCGCTCGATGGCCA | Amplification of the upstream fragment and amino acids 1 to 102 of CsiR (reverse) |
| SF-42-101R/102G-F | agtacaacGGTGGCTGTGGCGATGACGCCTGGGA | Amplification of the downstream fragment and amino acids 102 to 231 of CsiR (forward) |
| SF-42-101R/102G-R | ctgttGAAGCCTGCGCTGCGCTCGATGGCCAAGGCAAT | Amplification of the upstream fragment and amino acids 1 to 101 of CsiR (reverse) |
| SF-43-100E/101R-F | agtacaacGGTGGCTGTCGCGGCGATGACGCCTG | Amplification of the downstream fragment and amino acids 101 to 231 of CsiR (forward) |
| SF-43-100E/101R-R | ctgttGAAGCCTGCGCTCTCGATGGCCAAGGCAA | Amplification of the upstream fragment and amino acids 1 to 100 of CsiR (reverse) |
| SF-44-76S/77L-F | agtacaacGGTGGCTGTCTGGATGAGATGAACGA | Amplification of the downstream fragment and amino acids 77 to 231 of CsiR (forward) |
| SF-44-76S/77L-R | ctgttGAAGCCTGCGCTCGACATGGGCGCTACCC | Amplification of the upstream fragment and amino acids 1 to 76 of CsiR (reverse) |
| SF-45-75M/76S-F | agtacaacGGTGGCTGTTCGCTGGATGAGATGAACG | Amplification of the downstream fragment and amino acids 76 to 231 of CsiR (forward) |
| SF-45-75M/76S-R | ctgttGAAGCCTGCGCTCATGGGCGCTACCCGGT | Amplification of the upstream fragment and amino acids 1 to 75 of CsiR (reverse) |
| SF-46-74P/75M-F | agtacaacGGTGGCTGTATGTCGCTGGATGAGAT | Amplification of the downstream fragment and amino acids 75 to 231 of CsiR (forward) |
| SF-46-74P/75M-R | ctgttGAAGCCTGCGCTGGGCGCTACCCGGTAGC | Amplification of the upstream fragment and amino acids 1 to 74 of CsiR (reverse) |
| SF-47-73A/74P-F | agtacaacGGTGGCTGTCCCATGTCGCTGGATGA | Amplification of the downstream fragment and amino acids 74 to 231 of CsiR (forward) |
| SF-47-73A/74P-R | ctgttGAAGCCTGCGCTCGCTACCCGGTAGCCTT | Amplification of the upstream fragment and amino acids 1 to 73 of CsiR (reverse) |
| SF-48-72V/73A-F | agtacaacGGTGGCTGTGCGCCCATGTCGCTGGA | Amplification of the downstream fragment and amino acids 73 to 231 of CsiR (forward) |
| SF-48-72V/73A-R | ctgttGAAGCCTGCGCTTACCCGGTAGCCTTTCT | Amplification of the upstream fragment and amino acids 1 to 72 of CsiR (reverse) |
| SF-49-71R/72V-F | agtacaacGGTGGCTGTGTAGCGCCCATGTCGCTGG | Amplification of the downstream fragment and amino acids 72 to 231 of CsiR (forward) |
| SF-49-71R/72V-R | ctgttGAAGCCTGCGCTCCGGTAGCCTTTCTGGCTG | Amplification of the upstream fragment and amino acids 1 to 71 of CsiR (reverse) |
| SF-50-1M-F | ggagtacaacGGTGGCTGTATGGAAGCGCTCGCCCCCCG | Amplification of the downstream fragment and amino acids 1 to 231 of CsiR (forward) |
| SF-50-1M-R | gctgttGAAGCCTGCGCTCGGATCCTGGCTGTGGTG | Amplification of the upstream fragment of CsiR (reverse) |
| **Construction of Glusor-3 and Glusor (DBD truncation and linker mutagenesis)** | | |
| SF-15-nodbd-F1 | GCCCGCTAGAAGCTTGCGGCCGCATAATGCTTAAGTCGA | Amplification of the downstream fragment of plasmid backbone in pETDuet-1 (forward) |
| SF-15-nodbd-R1 | GTCGTTCATCTCATCCAGCGGATCCTGGCTGTGGTG | Amplification of the downstream fragment of plasmid backbone in pETDuet-1 (reverse) |
| SF-15-nodbd-F2 | CACCACAGCCAGGATCCGCTGGATGAGATGAACGAC | Amplification of the SF-15-nodbd (Glusor-3) (forward) |
| SF-15-nodbd-R2 | TTATGCGGCCGCAAGCTTCTAGCGGGCACCGATGCCTTC | Amplification of the SF-15-nodbd (Glusor-3) (reverse) |
| pETDuet-muta-F1 | TTTTCCGAAGAGGCGCTGGCGCTCAAGCGCCA | Amplification of the downstream fragment of plasmid backbone in pETDuet-1 (forward) |
| pETDuet-muta-R1 | CACCGTCTGCGTCAGCCACAGGTGGCGGTA | Amplification of the downstream fragment of plasmid backbone in pETDuet-1 (forward) |
| R2_GGC | ACAGCCACCgttgtactccagcttgtgccc | Amplification of downstream linker (350G351G352C) with cpSFYFP (reverse) |
| F2_S101_A | ctgacgcagacggtgGCGGCAGGCttcaacagcgacaa | Amplification of upstream linker (S101A) with cpSFYFP (forward) |
| F2_S101_C | ctgacgcagacggtgTGCGCAGGCttcaacagcgacaa | Amplification of upstream linker (S101C) with cpSFYFP (forward) |
| F2_S101_D | ctgacgcagacggtgGATGCAGGCttcaacagcgacaa | Amplification of upstream linker (S101D) with cpSFYFP (forward) |
| F2_S101_E | ctgacgcagacggtgGAAGCAGGCttcaacagcgacaa | Amplification of upstream linker (S101E) with cpSFYFP (forward) |
| F2_S101_F | ctgacgcagacggtgTTTGCAGGCttcaacagcgacaa | Amplification of upstream linker (S101F) with cpSFYFP (forward) |
| F2_S101_G | ctgacgcagacggtgGGCGCAGGCttcaacagcgacaa | Amplification of upstream linker (S101G) with cpSFYFP (forward) |
| F2_S101_H | ctgacgcagacggtgCATGCAGGCttcaacagcgacaa | Amplification of upstream linker (S101H) with cpSFYFP (forward) |
| F2_S101_I | ctgacgcagacggtgATTGCAGGCttcaacagcgacaac | Amplification of upstream linker (S101I) with cpSFYFP (forward) |
| F2_S101_K | ctgacgcagacggtgAAAGCAGGCttcaacagcgacaa | Amplification of upstream linker (S101K) with cpSFYFP (forward) |
| F2_S101_L | ctgacgcagacggtgCTGGCAGGCttcaacagcgacaa | Amplification of upstream linker (S101L) with cpSFYFP (forward) |
| F2_S101_M | ctgacgcagacggtgATGGCAGGCttcaacagcgacaa | Amplification of upstream linker (S101M) with cpSFYFP (forward) |
| F2_S101_N | ctgacgcagacggtgAACGCAGGCttcaacagcgacaa | Amplification of upstream linker (S101N) with cpSFYFP (forward) |
| F2_S101_P | ctgacgcagacggtgCCGGCAGGCttcaacagcgacaa | Amplification of upstream linker (S101P) with cpSFYFP (forward) |
| F2_S101_Q | ctgacgcagacggtgCAGGCAGGCttcaacagcgacaa | Amplification of upstream linker (S101Q) with cpSFYFP (forward) |
| F2_S101_R | ctgacgcagacggtgCGCGCAGGCttcaacagcgacaa | Amplification of upstream linker (S101R) with cpSFYFP (forward) |
| F2_S101_T | ctgacgcagacggtgACCGCAGGCttcaacagcgacaa | Amplification of upstream linker (S101T) with cpSFYFP (forward) |
| F2_S101_V | ctgacgcagacggtgGTGGCAGGCttcaacagcgacaa | Amplification of upstream linker (S101V) with cpSFYFP (forward) |
| F2_S101_W | ctgacgcagacggtgTGGGCAGGCttcaacagcgacaa | Amplification of upstream linker (S101W) with cpSFYFP (forward) |
| F2_S101_Y | ctgacgcagacggtgTATGCAGGCttcaacagcgacaa | Amplification of upstream linker (S101Y) with cpSFYFP (forward) |
| F2_A102_C | ctgacgcagacggtgAGCTGCGGCttcaacagcgacaa | Amplification of upstream linker (A102C) with cpSFYFP (forward) |
| F2_A102_D | ctgacgcagacggtgAGCGATGGCttcaacagcgacaa | Amplification of upstream linker (A102D) with cpSFYFP (forward) |
| F2_A102_E | ctgacgcagacggtgAGCGAAGGCttcaacagcgacaa | Amplification of upstream linker (A102E) with cpSFYFP (forward) |
| F2_A102_F | ctgacgcagacggtgAGCTTTGGCttcaacagcgacaac | Amplification of upstream linker (A102F) with cpSFYFP (forward) |
| F2_A102_G | ctgacgcagacggtgAGCGGCGGCttcaacagcgacaa | Amplification of upstream linker (A102G) with cpSFYFP (forward) |
| F2_A102_H | ctgacgcagacggtgAGCCATGGCttcaacagcgacaa | Amplification of upstream linker (A102H) with cpSFYFP (forward) |
| F2_A102_I | ctgacgcagacggtgAGCATTGGCttcaacagcgacaa | Amplification of upstream linker (A102I) with cpSFYFP (forward) |
| F2_A102_K | ctgacgcagacggtgAGCAAAGGCttcaacagcgacaa | Amplification of upstream linker (A102K) with cpSFYFP (forward) |
| F2_A102_L | ctgacgcagacggtgAGCCTGGGCttcaacagcgacaa | Amplification of upstream linker (A102L) with cpSFYFP (forward) |
| F2_A102_M | ctgacgcagacggtgAGCATGGGCttcaacagcgacaa | Amplification of upstream linker (A102M) with cpSFYFP (forward) |
| F2_A102_N | ctgacgcagacggtgAGCAACGGCttcaacagcgacaa | Amplification of upstream linker (A102N) with cpSFYFP (forward) |
| F2_A102_P | ctgacgcagacggtgAGCCCGGGCttcaacagcgacaa | Amplification of upstream linker (A102P) with cpSFYFP (forward) |
| F2_A102_Q | ctgacgcagacggtgAGCCAGGGCttcaacagcgacaa | Amplification of upstream linker (A102Q) with cpSFYFP (forward) |
| F2_A102_R | ctgacgcagacggtgAGCCGCGGCttcaacagcgacaa | Amplification of upstream linker (A102R) with cpSFYFP (forward) |
| F2_A102_S | ctgacgcagacggtgAGCAGCGGCttcaacagcgacaa | Amplification of upstream linker (A102S) with cpSFYFP (forward) |
| F2_A102_T | ctgacgcagacggtgAGCACCGGCttcaacagcgacaa | Amplification of upstream linker (A102T) with cpSFYFP (forward) |
| F2_A102_V | ctgacgcagacggtgAGCGTGGGCttcaacagcgacaa | Amplification of upstream linker (A102V) with cpSFYFP (forward) |
| F2_A102_W | ctgacgcagacggtgAGCTGGGGCttcaacagcgacaa | Amplification of upstream linker (A102W) with cpSFYFP (forward) |
| F2_A102_Y | ctgacgcagacggtgAGCTATGGCttcaacagcgacaa | Amplification of upstream linker (A102Y) with cpSFYFP (forward) |
| F2_G103_A | ctgacgcagacggtgAGCGCAGCGttcaacagcgacaa | Amplification of upstream linker (G103A) with cpSFYFP (forward) |
| F2_G103_C | ctgacgcagacggtgAGCGCATGCttcaacagcgacaa | Amplification of upstream linker (G103C) with cpSFYFP (forward) |
| F2_G103_D | ctgacgcagacggtgAGCGCAGATttcaacagcgacaa | Amplification of upstream linker (G103D) with cpSFYFP (forward) |
| F2_G103_E | ctgacgcagacggtgAGCGCAGAAttcaacagcgacaa | Amplification of upstream linker (G103E) with cpSFYFP (forward) |
| F2_G103_F | ctgacgcagacggtgAGCGCATTTttcaacagcgacaa | Amplification of upstream linker (G103F) with cpSFYFP (forward) |
| F2_G103_H | ctgacgcagacggtgAGCGCACATttcaacagcgacaa | Amplification of upstream linker (G103H) with cpSFYFP (forward) |
| F2_G103_I | ctgacgcagacggtgAGCGCAATTttcaacagcgacaa | Amplification of upstream linker (G103I) with cpSFYFP (forward) |
| F2_G103_K | ctgacgcagacggtgAGCGCAAAAttcaacagcgacaa | Amplification of upstream linker (G103K) with cpSFYFP (forward) |
| F2_G103_L | ctgacgcagacggtgAGCGCACTGttcaacagcgacaa | Amplification of upstream linker (G103L) with cpSFYFP (forward) |
| F2_G103_M | ctgacgcagacggtgAGCGCAATGttcaacagcgacaa | Amplification of upstream linker (G103M) with cpSFYFP (forward) |
| F2_G103_N | ctgacgcagacggtgAGCGCAAACttcaacagcgacaa | Amplification of upstream linker (G103N) with cpSFYFP (forward) |
| F2_G103_P | ctgacgcagacggtgAGCGCACCGttcaacagcgacaa | Amplification of upstream linker (G103P) with cpSFYFP (forward) |
| F2_G103_Q | ctgacgcagacggtgAGCGCACAGttcaacagcgacaa | Amplification of upstream linker (G103Q) with cpSFYFP (forward) |
| F2_G103_R | ctgacgcagacggtgAGCGCACGCttcaacagcgacaa | Amplification of upstream linker (G103R) with cpSFYFP (forward) |
| F2_G103_S | ctgacgcagacggtgAGCGCAAGCttcaacagcgacaa | Amplification of upstream linker (G103S) with cpSFYFP (forward) |
| F2_G103_T | ctgacgcagacggtgAGCGCAACCttcaacagcgacaa | Amplification of upstream linker (G103T) with cpSFYFP (forward) |
| F2_G103_V | ctgacgcagacggtgAGCGCAGTGttcaacagcgacaa | Amplification of upstream linker (G103V) with cpSFYFP (forward) |
| F2_G103_W | ctgacgcagacggtgAGCGCATGGttcaacagcgacaa | Amplification of upstream linker (G103W) with cpSFYFP (forward) |
| F2_G103_Y | ctgacgcagacggtgAGCGCATATttcaacagcgacaa | Amplification of upstream linker (G103Y) with cpSFYFP (forward) |
| F2_SAG | AGCGCAGGCTTCaacagcgacaacgtctat | Amplification of upstream linker (101S102A103G) with cpSFYFP (forward) |
| R2_G350_A | cgcctcttcggaaaaACAGCCCGCgttgtactccagctt | Amplification of downstream linker (G350A) with cpSFYFP (reverse) |
| R2_G350_C | cgcctcttcggaaaaACAGCCGCAgttgtactccagctt | Amplification of downstream linker (G350C) with cpSFYFP (reverse) |
| R2_G350_D | cgcctcttcggaaaaACAGCCATCgttgtactccagctt | Amplification of downstream linker (G350D) with cpSFYFP (reverse) |
| R2_G350_E | cgcctcttcggaaaaACAGCCTTCgttgtactccagctt | Amplification of downstream linker (G350E) with cpSFYFP (reverse) |
| R2_G350_F | gcctcttcggaaaaACAGCCAAAgttgtactccagcttg | Amplification of downstream linker (G350F) with cpSFYFP (reverse) |
| R2_G350_H | cgcctcttcggaaaaACAGCCATGgttgtactccagctt | Amplification of downstream linker (G350H) with cpSFYFP (reverse) |
| R2_G350_I | cgcctcttcggaaaaACAGCCAATgttgtactccagctt | Amplification of downstream linker (G350I) with cpSFYFP (reverse) |
| R2_G350_K | cgcctcttcggaaaaACAGCCTTTgttgtactccagctt | Amplification of downstream linker (G350K) with cpSFYFP (reverse) |
| R2_G350_L | cgcctcttcggaaaaACAGCCCAGgttgtactccagctt | Amplification of downstream linker (G350L) with cpSFYFP (reverse) |
| R2_G350_M | cgcctcttcggaaaaACAGCCCATgttgtactccagctt | Amplification of downstream linker (G350M) with cpSFYFP (reverse) |
| R2_G350_N | cgcctcttcggaaaaACAGCCGTTgttgtactccagctt | Amplification of downstream linker (G350N) with cpSFYFP (reverse) |
| R2_G350_P | cgcctcttcggaaaaACAGCCCGGgttgtactccagctt | Amplification of downstream linker (G350P) with cpSFYFP (reverse) |
| R2_G350_Q | cgcctcttcggaaaaACAGCCCTGgttgtactccagctt | Amplification of downstream linker (G350Q) with cpSFYFP (reverse) |
| R2_G350_R | cgcctcttcggaaaaACAGCCGCGgttgtactccagctt | Amplification of downstream linker (G350R) with cpSFYFP (reverse) |
| R2_G350_S | cgcctcttcggaaaaACAGCCGCTgttgtactccagctt | Amplification of downstream linker (G350S) with cpSFYFP (reverse) |
| R2_G350_T | cgcctcttcggaaaaACAGCCGGTgttgtactccagctt | Amplification of downstream linker (G350T) with cpSFYFP (reverse) |
| R2_G350_V | cgcctcttcggaaaaACAGCCCACgttgtactccagctt | Amplification of downstream linker (G350V) with cpSFYFP (reverse) |
| R2_G350_W | cgcctcttcggaaaaACAGCCCCAgttgtactccagctt | Amplification of downstream linker (G350W) with cpSFYFP (reverse) |
| R2_G350_Y | cgcctcttcggaaaaACAGCCATAgttgtactccagctt | Amplification of downstream linker (G350Y) with cpSFYFP (reverse) |
| R2_G351_A | cgcctcttcggaaaaACACGCACCgttgtactccagctt | Amplification of downstream linker (G351A) with cpSFYFP (reverse) |
| R2_G351_C | cgcctcttcggaaaaACAGCAACCgttgtactccagctt | Amplification of downstream linker (G351C) with cpSFYFP (reverse) |
| R2_G351_D | cgcctcttcggaaaaACAATCACCgttgtactccagctt | Amplification of downstream linker (G351D) with cpSFYFP (reverse) |
| R2_G351_E | cgcctcttcggaaaaACATTCACCgttgtactccagctt | Amplification of downstream linker (G351E) with cpSFYFP (reverse) |
| R2_G351_F | cgcctcttcggaaaaACAAAAACCgttgtactccagctt | Amplification of downstream linker (G351F) with cpSFYFP (reverse) |
| R2_G351_H | cgcctcttcggaaaaACAATGACCgttgtactccagctt | Amplification of downstream linker (G351H) with cpSFYFP (reverse) |
| R2_G351_I | cgcctcttcggaaaaACAAATACCgttgtactccagctt | Amplification of downstream linker (G351I) with cpSFYFP (reverse) |
| R2_G351_K | cgcctcttcggaaaaACATTTACCgttgtactccagctt | Amplification of downstream linker (G351K) with cpSFYFP (reverse) |
| R2_G351_L | cgcctcttcggaaaaACACAGACCgttgtactccagctt | Amplification of downstream linker (G351L) with cpSFYFP (reverse) |
| R2_G351_M | cgcctcttcggaaaaACACATACCgttgtactccagctt | Amplification of downstream linker (G351M) with cpSFYFP (reverse) |
| R2_G351_N | cgcctcttcggaaaaACAGTTACCgttgtactccagctt | Amplification of downstream linker (G351N) with cpSFYFP (reverse) |
| R2_G351_P | cgcctcttcggaaaaACACGGACCgttgtactccagctt | Amplification of downstream linker (G351P) with cpSFYFP (reverse) |
| R2_G351_Q | cgcctcttcggaaaaACACTGACCgttgtactccagctt | Amplification of downstream linker (G351Q) with cpSFYFP (reverse) |
| R2_G351_R | cgcctcttcggaaaaACAGCGACCgttgtactccagctt | Amplification of downstream linker (G351R) with cpSFYFP (reverse) |
| R2_G351_S | cgcctcttcggaaaaACAGCTACCgttgtactccagctt | Amplification of downstream linker (G351S) with cpSFYFP (reverse) |
| R2_G351_T | cgcctcttcggaaaaACAGGTACCgttgtactccagctt | Amplification of downstream linker (G351T) with cpSFYFP (reverse) |
| R2_G351_V | cgcctcttcggaaaaACACACACCgttgtactccagctt | Amplification of downstream linker (G351V) with cpSFYFP (reverse) |
| R2_G351_W | cgcctcttcggaaaaACACCAACCgttgtactccagctt | Amplification of downstream linker (G351W) with cpSFYFP (reverse) |
| R2_G351_Y | cgcctcttcggaaaaACAATAACCgttgtactccagctt | Amplification of downstream linker (G351Y) with cpSFYFP (reverse) |
| R2_C352_A | cgcctcttcggaaaaCGCGCCACCgttgtactccagctt | Amplification of downstream linker (C352A) with cpSFYFP (reverse) |
| R2_C352_D | cgcctcttcggaaaaATCGCCACCgttgtactccagctt | Amplification of downstream linker (C352D) with cpSFYFP (reverse) |
| R2_C352_E | cgcctcttcggaaaaTTCGCCACCgttgtactccagctt | Amplification of downstream linker (C352E) with cpSFYFP (reverse) |
| R2_C352_F | gcctcttcggaaaaAAAGCCACCgttgtactccagcttG | Amplification of downstream linker (C352F) with cpSFYFP (reverse) |
| R2_C352_G | gcctcttcggaaaaGCCGCCACCgttgtactccagcttG | Amplification of downstream linker (C352G) with cpSFYFP (reverse) |
| R2_C352_H | cgcctcttcggaaaaATGGCCACCgttgtactccagctt | Amplification of downstream linker (C352H) with cpSFYFP (reverse) |
| R2_C352_I | cgcctcttcggaaaaAATGCCACCgttgtactccagctt | Amplification of downstream linker (C352I) with cpSFYFP (reverse) |
| R2_C352_K | cgcctcttcggaaaaTTTGCCACCgttgtactccagctt | Amplification of downstream linker (C352K) with cpSFYFP (reverse) |
| R2_C352_L | cgcctcttcggaaaaCAGGCCACCgttgtactccagctt | Amplification of downstream linker (C352L) with cpSFYFP (reverse) |
| R2_C352_M | cgcctcttcggaaaaCATGCCACCgttgtactccagctt | Amplification of downstream linker (C352M) with cpSFYFP (reverse) |
| R2_C352_N | gcctcttcggaaaaGTTGCCACCgttgtactccagcttG | Amplification of downstream linker (C352N) with cpSFYFP (reverse) |
| R2_C352_P | cgcctcttcggaaaaCGGGCCACCgttgtactccagctt | Amplification of downstream linker (C352P) with cpSFYFP (reverse) |
| R2_C352_Q | cgcctcttcggaaaaCTGGCCACCgttgtactccagctt | Amplification of downstream linker (C352Q) with cpSFYFP (reverse) |
| R2_C352_R | gcctcttcggaaaaGCGGCCACCgttgtactccagcttG | Amplification of downstream linker (C352R) with cpSFYFP (reverse) |
| R2_C352_S | gcctcttcggaaaaGCTGCCACCgttgtactccagcttG | Amplification of downstream linker (C352S) with cpSFYFP (reverse) |
| R2_C352_T | gcctcttcggaaaaGGTGCCACCgttgtactccagcttG | Amplification of downstream linker (C352T) with cpSFYFP (reverse) |
| R2_C352_V | cgcctcttcggaaaaCACGCCACCgttgtactccagctt | Amplification of downstream linker (C352V) with cpSFYFP (reverse) |
| R2_C352_W | cgcctcttcggaaaaCCAGCCACCgttgtactccagctt | Amplification of downstream linker (C352W) with cpSFYFP (reverse) |
| R2_C352_Y | cgcctcttcggaaaaATAGCCACCgttgtactccagctt | Amplification of downstream linker (C352Y) with cpSFYFP (reverse) |
| **Construction of Glusor mutant related to glutarate binding** | | |
| Glusor-muta-F1 | aacGGCTGTTTTTCCGAAGAGGCGCTGGCGCTCAA | Amplification of CsiR-LBD with mutations and downstream fragment of pETDuet-1 plasmid backbone (forward) |
| Glusor-muta-R1 | GAAGCCTGCggcCACCGTCTGCGTCAGCCACA | Amplification of CsiR-LBD with mutations and downstream fragment of pETDuet-1 plasmid backbone (reverse) |
| Glusor-muta-F2 | gccGCAGGCTTCaacagcgacaacgtctatatc | Amplification of cpSFYFP from Glusor (forward) |
| Glusor-muta-R2 | CTTCGGAAAAACAGCCgttgttgtactccagcttg | Amplification of cpSFYFP from Glusor (reverse) |
| **Expression in mammalian cells** | | |
| pcDNA3.1^(+)^-cpSFYFP-F1 | GCCACAAGCTGGAGTACAATTAATCTAGAGGGCCCGTTT | Inverse amplification of the plasmid backbone of pcDNA3.1^(+)^-cpSFYFP (forward) |
| pcDNA3.1^(+)^-cpSFYFP-R1 | TACACGTTATCGCTATTGAACATGGTGGCGGATCCGAGC | Inverse amplification of the plasmid backbone of pcDNA3.1^(+)^-cpSFYFP (reverse) |
| pcDNA3.1^(+)^-cpSFYFP-F2 | GCTCGGATCCGCCACCATGTTCAATAGCGATAACGTGTA | Amplification of cpSFYFP (forward) |
| pcDNA3.1^(+)^-cpSFYFP-R2 | AAACGGGCCCTCTAGATTAATTGTACTCCAGCTTGTGGC | Amplification of cpSFYFP (reverse) |
| **Expression inside mitochondria** | | |
| Mito-Glusor-reverse-F | tgataagcggccgctcgagtctaga | Inverse amplification of the plasmid backbone of pcDNA3.1^(+)^ construction (forward) |
| Mito-Glusor-reverse-R | CATGGTGGCGCTAGCCAGCTTGGGT | Inverse amplification of the plasmid backbone of pcDNA3.1^(+)^ construction (reverse) |
| Mito-Glusor-F1 | CCAAGCTGGCTAGCGCCACCATGCTATCACTGCGACAGT | Amplification of Mito target sequence for Mito-Glusor construction (forward) |
| Mito-Glusor-R1 | CATGAGTAGGTATCGCGATGAGCAC | Amplification of Mito target sequence for Mito-Glusor construction (reverse) |
| Mito-Glusor-F2 | GCTCATCGCGATACCTACTCatgctggatgagatgaacg | Amplification of Glusor for Mito-Glusor construction (forward) |
| Mito-Glusor-R2 | gactcgagcggccgcttatcacctggcgccgatgccctc | Amplification of Glusor for Mito-Glusor construction (reverse) |
| Mito-cpSFYFP-reverse-F | GCAGTGATAGCATGGTGGCGGATCCGAGCTCGGTACCAA | Inverse amplification of the plasmid backbone of pcDNA3.1^(+)^ construction (forward) |
| Mito-cpSFYFP-reverse-R | ACAAGCTGGAGTACAATTAATCTAGAGGGCCCGTTTAAA | Inverse amplification of the plasmid backbone of pcDNA3.1^(+)^ construction (reverse) |
| Mito-cpSFYFP-F1 | CGAGCTCGGATCCGCCACCATGCTATCACTGCGACAGTC | Amplification of Mito target sequence for Mito-cpSFYFP construction (forward) |
| Mito-cpSFYFP-R1 | ACACGTTATCGCTATTGAACATGAGTAGGTATCGCGATG | Amplification of Mito target sequence for Mito-cpSFYFP construction (reverse) |
| Mito-cpSFYFP-F2 | CATCGCGATACCTACTCatgTTCAATAGCGATAACGTGT | Amplification of cpSFYFP for Mito-cpSFYFP construction (forward) |
| Mito-cpSFYFP-R2 | TTTAAACGGGCCCTCTAGATTAATTGTACTCCAGCTTGT | Amplification of cpSFYFP for Mito-cpSFYFP construction (reverse) |
| **Expression inside cytosol** | | |
| Cyto-Glusor-reverse-F | gggcatcggcgccaggtgataagcggccgctcgagtcta | Inverse amplification of the plasmid backbone of pcDNA3.1^(+)^ construction (forward) |
| Cyto-Glusor-reverse-R | GCTTCTTCTGCAGAGCCATggtggcgctagccagcttgg | Inverse amplification of the plasmid backbone of pcDNA3.1^(+)^ construction (reverse) |
| Cyto-Glusor-F1 | caagctggctagcgccaccATGGCTCTGCAGAAGAAGCT | Amplification of Cyto target sequence for Cyto-Glusor construction (forward) |
| Cyto-Glusor-R1 | GGAAGAGACTGGAGGACCTGatgctggatgagatgaacg | Amplification of Cyto target sequence for Cyto-Glusor construction (reverse) |
| Cyto-Glusor-F2 | GAAGAGACTGGAGGACCTGatgctggatgagatgaacga | Amplification of Glusor for Cyto-Glusor construction (forward) |
| Cyto-Glusor-R2 | agggcatcggcgccaggtgataagcggccgctcgagtct | Amplification of Glusor for Cyto-Glusor construction (reverse) |
| Cyto-cpSFYFP-reverse-F | CCACAAGCTGGAGTACAATTAATCTAGAGGGCCCG | Inverse amplification of the plasmid backbone of pcDNA3.1^(+)^ construction (forward) |
| Cyto-cpSFYFP-reverse-R | CAGCTTCTTCTGCAAGGCCATGGTGGCGGATCCGAGCTC | Inverse amplification of the plasmid backbone of pcDNA3.1^(+)^ construction (reverse) |
| Cyto-cpSFYFP-F1 | ATCCGCCACCATGGCCTTGCAGAAGAAGCT | Amplification of Cyto target sequence for Cyto-cpSFYFP construction (forward) |
| Cyto-cpSFYFP-R1 | CGTTATCGCTATTGAAcatCAAATCCTCAAGGCGCTTTC | Amplification of Cyto target sequence for Cyto-cpSFYFP construction (reverse) |
| Cyto-cpSFYFP-F2 | CGCCTTGAGGATTTGatgTTCAATAGCGAT | Amplification of cpSFYFP for Cyto-cpSFYFP construction (forward) |
| Cyto-cpSFYFP-R2 | CGGGCCCTCTAGATTAATTGTACTCCAGCTTGTGG | Amplification of cpSFYFP for Cyto-cpSFYFP construction (reverse) |
| **Expression inside nucleus** | | |
| Nuc-Glusor-reverse-F | AAAGAAGAAGAGAAAGGTAtgataagcggccgctcgagt | Inverse amplification of the plasmid backbone of pcDNA3.1^(+)^ construction (forward) |
| Nuc-Glusor-reverse-R | cgttcatctcatccagcatggtggcgctagccagcttgg | Inverse amplification of the plasmid backbone of pcDNA3.1^(+)^ construction (reverse) |
| Nuc-Glusor-F1 | ccgagggcatcggcgccaggGATCCAAAAAAGAAGAGAA | Amplification of Nuc target sequence for Nuc-Glusor construction (forward) |
| Nuc-Glusor-R1 | actcgagcggccgcttatcaTACCTTTCTCTTCTTCTTT | Amplification of Nuc target sequence for Nuc-Glusor construction (reverse) |
| Nuc-Glusor-F2 | ccaagctggctagcgccaccatgctggatgagatgaacg | Amplification of Glusor for Nuc-Glusor construction (forward) |
| Nuc-Glusor-R2 | TTCTCTTCTTTTTTGGATCcctggcgccgatgccctcgg | Amplification of Glusor for Nuc-Glusor construction (reverse) |
| Nuc-cpSFYFP-reverse-F | AGAAGAAGAGAAAGGTATAATCTAGAGGGCCCGTTTAAA | Inverse amplification of the plasmid backbone of pcDNA3.1^(+)^ construction (forward) |
| Nuc-cpSFYFP-reverse-R | ACACGTTATCGCTATTGAAcatGGTGGCGGATCCGAGCT | Inverse amplification of the plasmid backbone of pcDNA3.1^(+)^ construction (reverse) |
| Nuc-cpSFYFP-F1 | GCCACAAGCTGGAGTACAATGATCCAAAAAAGAAGAGAA | Amplification of Nuc target sequence for Nuc-cpSFYFP construction (forward) |
| Nuc-cpSFYFP-R1 | TTTAAACGGGCCCTCTAGATTATACCTTTCTCTTCTTCT | Amplification of Nuc target sequence for Nuc-cpSFYFP construction (reverse) |
| Nuc-cpSFYFP-F2 | AGCTCGGATCCGCCACCatgTTCAATAGCGATAACGTGT | Amplification of cpSFYFP for Nuc-cpSFYFP construction (forward) |
| Nuc-cpSFYFP-R2 | TTCTCTTCTTTTTTGGATCATTGTACTCCAGCTTGTGGC | Amplification of cpSFYFP for Nuc-cpSFYFP construction (reverse) |
| **Guide sequence for CRISPR-Cas9 knockout** | | |
| N20-*csiD*-F | tagtGATGGTGAAGCTGGCAACGG | Guide sequence for targeting the *csiD* of *E. coli* BL21(DE3) (forward) |
| N20-*csiD*-R | aaacCCGTTGCCAGCTTCACCATC | Guide sequence for targeting the *csiD* of *E. coli* BL21(DE3) (reverse) |
| N20-*kgtP*-F | tagtTCCGACAGCGCGCCAATGAG | Guide sequence for targeting the *kgtP* of *E. coli* BL21(DE3) (forward) |
| N20-*kgtP*-R | aaacCTCATTGGCGCGCTGTCGGA | Guide sequence for targeting the *kgtP* of *E. coli* BL21(DE3) (reverse) |
| N20-*dctA*-F | tagtGCTGGCGGCGACGCTCTCTG | Guide sequence for targeting the *dctA* of *E. coli* BL21(DE3) (forward) |
| N20-*dctA*-R | aaacCAGAGAGCGTCGCCGCCAGC | Guide sequence for targeting the *dctA* of *E. coli* BL21(DE3) (reverse) |
| N20-*dauA*-F | tagtGGGATTGTTAACCTGCTCGG | Guide sequence for targeting the *dauA* of *E. coli* BL21(DE3) (forward) |
| N20-*dauA*-R | aaacCCGAGCAGGTTAACAATCCC | Guide sequence for targeting the *dauA* of *E. coli* BL21(DE3) (reverse) |
| N20-*dcuA*-F | tagtGAGGGCCTGGTTGAACTGCG | Guide sequence for targeting the *dcuA* of *E. coli* BL21(DE3) (forward) |
| N20-*dcuA*-R | aaacCGCAGTTCAACCAGGCCCTC | Guide sequence for targeting the *dcuA* of *E. coli* BL21(DE3) (reverse) |
| N20-*ynfM*-F | TAGTcctggcatgtcagtcaggcc | Guide sequence for targeting the *ynfM* of *E. coli* BL21(DE3) (forward) |
| N20-*ynfM*-R | aaacggcctgactgacatgccagg | Guide sequence for targeting the *ynfM* of *E. coli* BL21(DE3) (reverse) |
